# Direct Access to Substituted 4-CF_3_ β-Lactams at the C-3 Position

**DOI:** 10.3389/fchem.2019.00526

**Published:** 2019-08-06

**Authors:** Monika Skibińska, Marcin Kaźmierczak, Thierry Milcent, Tomasz Cytlak, Henryk Koroniak, Benoit Crousse

**Affiliations:** ^1^Faculty of Chemistry, Adam Mickiewicz University, Poznań, Poland; ^2^Faculté de Pharmacie, UMR 8076 CNRS, BioCIS, University of Paris-Sud, Univ. Paris-Saclay, Châtenay-Malabry, France; ^3^Centre for Advanced Technologies, Adam Mickiewicz University, Poznań, Poland

**Keywords:** lactams, fluorine, alkylation, deprotonation, functionalization

## Abstract

Mono- and disubstituted 4-CF_3_ β-lactams at the C-3 position have been obtained stereoselectively under basic conditions. A wide range of function such as alcohols, alkyls, aryls, esters, and double and triple bonds have been introduced.

**GRAPHICAL ABSTRACT:**
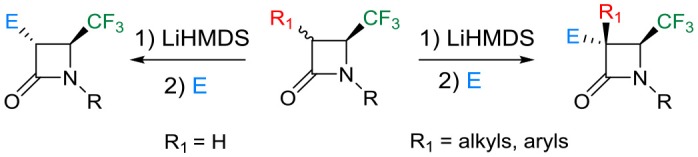
Stereoselective access to C-3 mono- and disubstituted 4-CF_3_ β-lactams under basic conditions.

## Introduction

β-Lactams (2-azetidinones) are of major interest not only for their biological properties, such as antibiotics (Georg, 1993) and enzyme inhibitors (Alcaide et al., 2007), but also for their usefulness as intermediates in organic chemistry, for example, in the synthesis of peptidomimetics and alkaloids (Ojima, 1995). 2-Azetidinones motivated the scientific community to study and exploit them. Thus, several methods of β-lactam synthesis have been developed (Pitts and Letcka, 2014; Hosseyni and Jarrahpour, 2018).

**Scheme 1:**
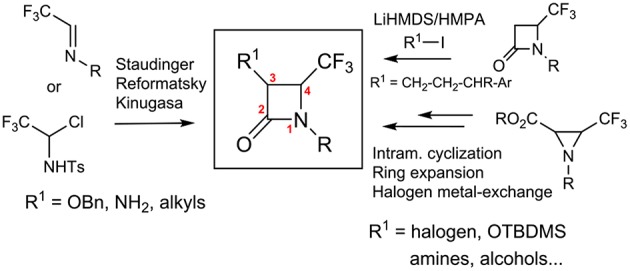
Approaches of C-3 monosubstituted 4-CF_3_ β-lactams.

Although fluorine-containing compounds have been widely used in the field of medicinal chemistry (Wang et al., 2014; Zhu et al., 2014; Zhou et al., 2016) due to their pharmacological properties, 4-CF_3_ monobactams functionalized at C-3 are slightly exploited. The synthesis of 4-CF_3_ monosubstituted β-lactams at the C-3 position are reported and prepared according to different ways: Staudinger reaction (Guanti et al., 1985; Abouabdellah et al., 1997; Petrik et al., 2011), ring expansion of aziridines via halogen-metal exchange (Decamps et al., 2014), Kinugasa reaction (Kowalski et al., 2016), and the Reformatsky reaction (Trulli et al., 2018). The alkylation reaction with non-fluorinated β-lactams using lithium amide, such as LDA and Li/KHDMS, to obtain the alkylated products at C-3 was well documented in the literature (Kuhlein and Jensen, 1974; Kamath and Ojima, 2012; Deketelaere et al., 2017). Surprisingly, only one example is reported involving the formation of enolate of 4-CF_3_ β-lactams under the condition of LiHMDS/HMPA with alkyl iodides (Liu et al., 2013) (Scheme 1).

**Scheme 2:**
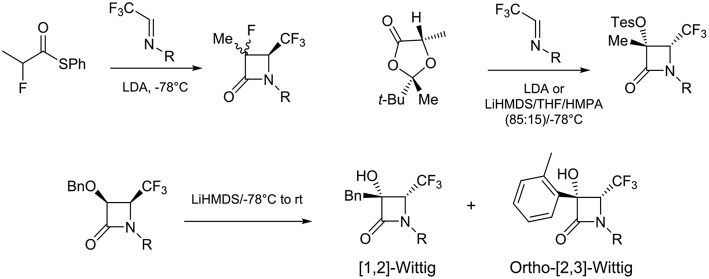
Approaches of C-3 disubstituted 4-CF_3_ β-lactams.

To the best of our knowledge, only few examples of the synthesis of disubstituted β-lactams at the C-3 position have been reported in the literature. We can cite the stereoselective synthesis of 4-CF_3_-3-fluoroazetidinones via the condensation of 2-fluoropropanethioate lithium enolate with CF_3_-imines (Ishihara et al., 1996), the addition of enolate to trifluoromethyl-*N*-*para*-methoxyphenyl imine that gave a 4:1 diastereomeric mixture of β-lactams (3R,4R, *ee*: 72%) and (3R,4S, *ee*: 72%) (Battaglia et al., 2003), and the Wittig rearrangement (Garbi et al., 2001) (Scheme 2).

## Results and Discussion

Herein, we disclose the straightforward synthesis of mono- and difunctionalized 4-CF_3_ β-lactams at the C-3 position. First, 4-CF_3_ β-lactams **1** and **2** were synthesized according to the literature (Trulli et al., 2018), and the same method was applied to the synthesis of the *N*-PMBn protected 4-CF_3_ β-lactam **3**. Then, we attempted the C-3 monodeprotonation of the 4-CF_3_ β-lactams **1–3** (Trulli et al., 2018) with diverse bases such as LDA, LiHMDS, and LTMP. Thus, the addition of benzaldehyde at different conditions (temperature, time, concentration of base) was conducted. The best conditions were achieved when 1.5 eq of LiHMDS in THF at −25°C was applied for 30 min, followed by the addition of 2 eq of aldehyde. The reaction mixture was kept for an additional 2 h at the same temperature and then it was let warm at room temperature overnight. The reaction is stereoselective and the best yields in corresponding 3-functionalized β-lactams **4** were obtained from **1** possessing the *para*-methoxyphenyl group. From β-lactams **2** and **3**, moderate yields were obtained. Our significant results are summarized in the following Table 1. The relative configuration was determined by ^1^H-NMR according to the coupling constant of *cis* (3R^*^, 4S^*^) (6 Hz) and *trans* (3S^*^, 4S^*^) (3 Hz) between H-3 and H-4. The addition occurred exclusively from the opposite side of the bulky trifluoromethyl substituent. This result has already been observed during our reactions of Li/Br exchange from 3-Br 4-CF_3_ lactams and aldehydes (Decamps et al., 2014).

**Table 1:** Synthesis of C-3 functionalizated 4-CF_3_ β-lactams **4–6**.

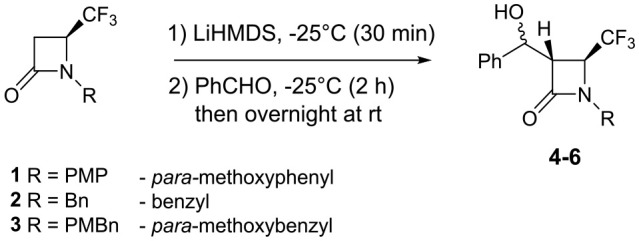				
**Entry**	**4-CF_**3**_ β-lactam**	**LiHMDS**	**4-6 *(dr)^b^***	**Yield (%)*^a^***
1	**1**	3 eq	**4a,b** (1/0.8)	76
2	**1**	2 eq	**4a,b** (1/0.7)	81
3	**1**	1.5 eq	**4a,b** (1/0.7)	83
4	**2**	1.5 eq	**5a,b** (0.4/1)	57
5	**3**	1.5 eq	**6a,b** (0.5/1)	60

a *Isolated yields*.

b Related to the newly formed stereocenter at benzylic carbon. Stereochemistry of the β-lactam ring is trans (3S^*^,4S^*^).

With these efficient conditions in hand, other electrophilic reagents have been investigated with the 4-CF_3_ β-lactam **1** (Table 2). Aldehydes were trapped by the enolate to afford the corresponding 3-functionalized 4-CF_3_ β-lactams **7** and **8** as a mixture of diastereoisomers. Products were isolated and separated in good yields and with excellent stereoselectivity. The relative configuration of compounds **7** and **8** is *trans* (3S^*^, 4S^*^). Compounds **7** and **8** prepared from Li/Br exchange at −100°C were obtained with an inverse stereoselectivity (Decamps et al., 2014). Next, the reactivity of alkyl halides has been undertaken. When reaction was performed with 1.2 eq of MeI, a mixture of C-3 mono- and disubstituted lactams **9** and **14** was obtained (Table 2, entry 3). Thus, with 2 eq of MeI, the product **14** was exclusively obtained in good yield (68%) (Table 2, entry 4). Similarly, with 1.2 eq of EtI, the same dependence was observed (Table 2, entry 5), but with 2 eq of EtI, C-3 disubstituted product **15** was obtained in much lower yield compared to **14** (Table 2, entry 6), due to the formation of a large number of unexpected products. From 1.2 eq of allyl bromide, C-3 mono- and disubstituted lactams were isolated (Table 2, entry 7). Surprisingly, with the propargyl bromide, only the C-3 monosubstituted azetidinone **12** was obtained (Table 2, entry 8). Regrettably, when 2 eq or more of allyl or propargyl bromide was used, many side products were present in the crude mixture. With another type of electrophile, the ethyl chloroformate, the C-3 monosubstituted lactam was obtained with 1.2 eq, and a mixture of C-3 mono- and disubstituted was observed with 2 eq (Table 2, entries 9 and 10, respectively). No improvement was observed when temperature and solvent, base, and its concentration were modified. Moreover, in almost each case, yields of the products were poor to moderate.

**Table 2:** Reactions of C-3 substituted 4-CF_3_ β-lactam **1** with electrophiles.

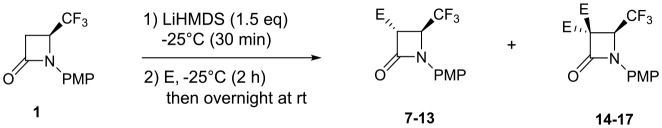				
**Entry**	**Electrophile**	***(dr)***	**Yield (%) (7–13)*^a^***	**Yield (%) (14–17)*^a^***
1	*p*-BrC_6_H_4_CHO (2 eq)	1/0.5	54 (**7a,b**)*^b^*	–
2	*p*-MeOC_6_H_4_CHO (2 eq)	1/0.5	57 (**8a,b**)*^*b*^*	–
3	MeI (1.2 eq)	–	15 (**9**)	30 (**14**)
4	MeI (2 eq)	-	–	68 (**14**)
5	EtI (1.2 eq)	–	12 (**10**)	23 (**15**)
6	EtI (2 eq)	–	–	33 (**15**)
7	Allyl bromide (1.2 eq)	–	26 (**11**)	43 (**16**)
8	Propargyl bromide (1.2 eq)	–	40 (**12**)	–
9	Ethyl chloroformate (1.2 eq)	-	28 (**13**)	–
10	Ethyl chloroformate (2 eq)	–	47 (**13**)	35 (**17**)

a *Isolated yields*.

b Related to the newly formed stereocenter at benzylic carbon. Stereochemistry of the β-lactam ring is trans (3S^*^,4S^*^).

**Scheme 3:**
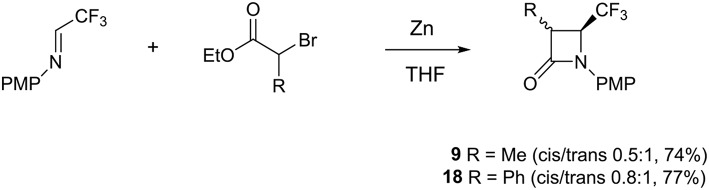
Reformatsky reaction of PMP-aldimine with methyl- and phenyl-α-bromo esters.

Due to these disappointing results, we moved toward a new pathway that focused on rapid access of C-3 disubstituted 4-CF_3_ β-lactams from C-3 monosubstituted 4-CF_3_ β-lactams **9** and **18**. Indeed 4-CF_3_ β-lactams possessing quaternary stereogenic center at C-3 are quite limited. The C-3 monosubstituted 4-CF_3_ β-lactams **9** and **18** have been prepared using the Reformatsky reaction between CF_3_-aldimine and methyl- and phenyl-α-bromo esters (Scheme 3). The 3-Me 4-CF_3_ β-lactams **9** were prepared according to the literature (Trulli et al., 2018) but with shorter reaction time (6 h). For this reason, we observed different ratio of *cis* (3S^*^,4S^*^) and *trans* (3R^*^,4S^*^) isomers (0.5:1, respectively) than was reported. The same method was incorporated to synthesize 3-Ph 4-CF_3_ β-lactams **18**, which were formed also as a mixture of *cis* (3S^*^,4S^*^) and *trans* (3R^*^,4S^*^) isomers (0.8:1, respectively) (Scheme 3).

**Scheme 4:**
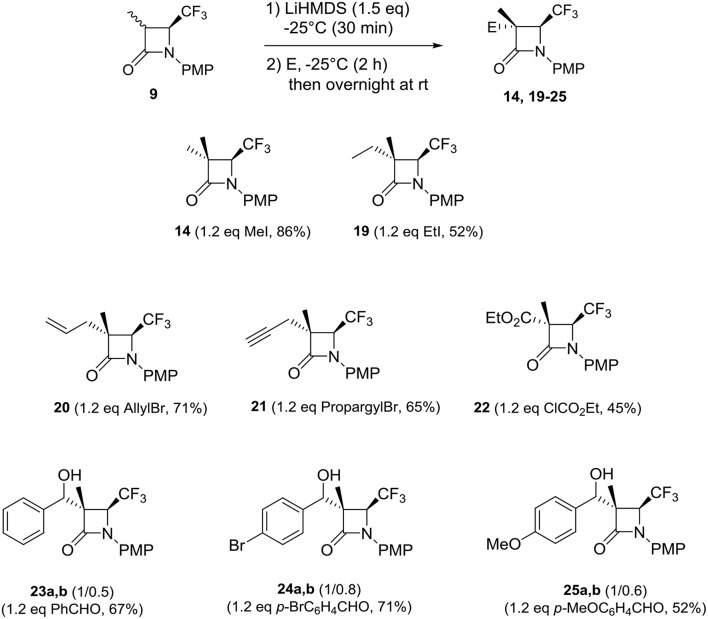
Reactions of 3-Me 4-CF_3_ β-lactams **9** with electrophiles.

To synthesize C-3 disubstituted 4-CF_3_ β-lactams, we started our studies with the previous conditions involving LiHMDS in THF at −25°C and MeI as electrophile with the 3-Me 4-CF_3_ β-lactams **9**. Fortunately, the corresponding C-3 disubstituted compound **14** was obtained in excellent yield (86%). Due to this new result, we investigated first the synthesis of non-symmetrical C-3 disubstituted 4-CF_3_ β-lactams from **9** (Scheme 4). From alkyl halides such as EtI, allyl, and propargyl bromides corresponding to 4-CF_3_ β-lactams **19–21** were obtained in reasonable to good yields and in excellent stereochemical purity (Scheme 4). From the ethyl chloroformate, the lactam **22** was obtained in 45% yield. Interestingly, 4-CF_3_ β-lactams **20–22** are very attractive because of the presence of various functions for functionalizing them or incorporating into bioactive molecules for example. Likewise, the reaction gave very good results with the aldehydes, which led to alcohols **23–25** with an excellent stereoselectivity.

**Scheme 5:**
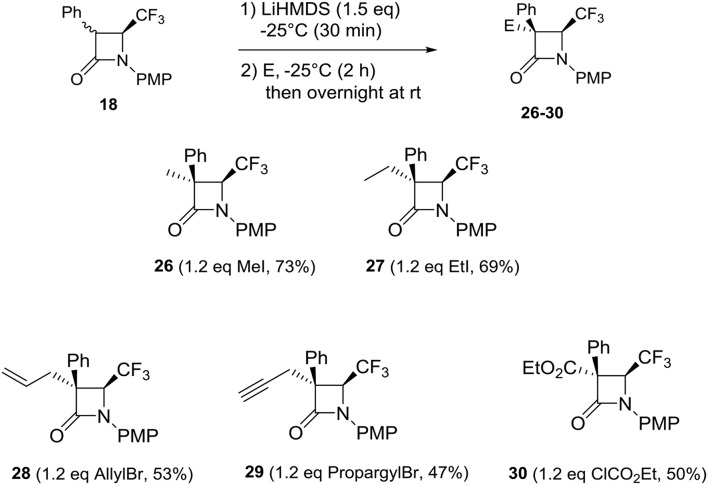
Reactions of 3-Ph 4-CF_3_ β-lactams **18** with electrophiles.

Then, we investigated reactions of 3-Ph 4-CF_3_ β-lactams **18** with the same electrophiles using the previous conditions (Scheme 5). Unfortunately, with aldehydes, only traces of products were detected. This surprising result could be explained by the steric hindrance of the 3-Ph group in **18** relative to the 3-Me group in **9**. Nevertheless, we investigated the reactions with the other electrophiles. In the case of the methyl and ethyl iodides, the reactions occurred to incorporate the methyl and the ethyl group in α position of the phenyl group. Compounds **26** and **27** were isolated in good yield, 73 and 69%, respectively. Reasonable yields were obtained with the allyl and the propargyl bromides, 53% (**28**) and 47% (**29**), respectively. The chloroformate reacted with the enolate intermediate, leading to the desired 4-CF_3_ β-lactam **30** in 50% yield.

**Scheme 6:**
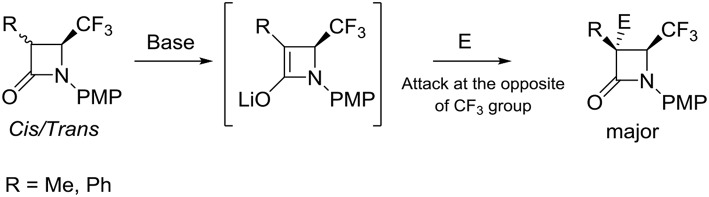
Stereochemistry of addition of electrophiles to enolate of C-3 substituted 4-CF_3_ β-lactams.

All products **14** and **19–30** were isolated pure and with an excellent stereoselectivity. Furthermore, if reactions were performed with lactams **9** or **18** as a *cis* or *trans* or in mixture, the results were the same, which means that the enolate intermediate is identical regardless of the ratio *cis/trans*. Then, the addition of the electrophile occurred at the opposite of the CF_3_ group. Thus, we can suppose the following mechanism reported in Scheme 6.

The configuration of the addition products of different electrophiles to enolate was studied and determined by NMR analysis of compounds **19–30**. For each product, 2D ^1^H-^19^F HOESY NMR spectra were performed. We observed correlation between the CF_3_ group and the 3-Me or 3-Ph group and correlation between CF_3_ and geminal proton due to the bulkiness of this substituent. As an example, for the product **23a**, the ^1^H-^19^F HOESY spectrum (see Supplementary Figure 1) showed correlations for the both mentioned interactions of the C***H***_3_ with C***F***_3_ as well as C***H***CF_3_ with C***F***_3_ on the same intensity level.

**Scheme 7:**
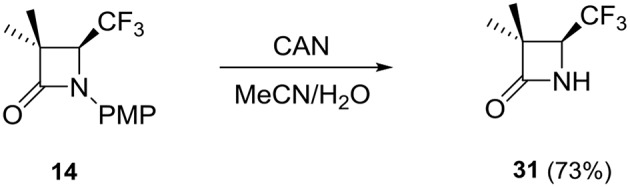
*N*-PMP deprotection of **14**.

Furthermore, the effortless and efficient method of *N*-PMP deprotection of the selected 4-CF_3_ β-lactam (**14**), using ceric ammonium nitrate (CAN) (Jarrahpour and Zarei, 2007), showed great synthetic opportunity toward preparation of β-lactam antibiotics and application in the semi-synthesis of anticancer agents (Zarei et al., 2012) (Scheme 7).

## Materials and Methods

### Experimental Section

#### General Methods

^1^H NMR, ^13^C NMR, ^19^F NMR, and ^31^P NMR spectra were performed on Bruker ASCEND 600 (600 MHz) spectrometers. All 2D and 1D selective NMR spectra were recorded on a Bruker ASCEND 600 (600 MHz) spectrometer. Chemical shifts of ^1^H NMR were expressed in parts per million downfield from tetramethylsilane (TMS) as an internal standard (δ = 0) in CDCl_3_. Chemical shifts of ^13^C NMR were expressed in parts per million downfield and upfield from CDCl_3_ as an internal standard (δ = 77.0). Chemical shifts of ^19^F NMR were expressed in parts per million upfield from CFCl_3_ as an internal standard (δ = 0) in CDCl_3_. All d.r. were evaluated on the basis of ^19^F NMR reaction mixture. High-resolution mass spectra were recorded by electron spray (MS-ESI) techniques using a QToF Impact HD Bruker spectrometer. Reagent grade chemicals were used. THF was dried by refluxing with sodium metal-benzophenone (THF) and distilled under argon atmosphere. All moisture-sensitive reactions were carried out under argon atmosphere using oven-dried glassware. Reaction temperatures below 0°C were performed using a cooling bath (liquid N_2_/*n*-hexane or liquid N_2_/*i*-PrOH). TLC was performed on Merck Kieselgel 60-F254 with EtOAc/*n*-hexane and MeOH/CHCl_3_ as developing systems, and products were detected by inspection under UV light (254 nm) and with a solution of potassium permanganate. Merck Kieselgel 60 (0.063–0.200 μm), Merck Kieselgel 60 (0.040–0.063 μm), and Merck Kieselgel 60 (0.015–0.004 μm) were used for column chromatography.

### General Procedure of the Synthesis Reformatsky Reaction of PMP-Aldimine With Methyl- and Phenyl-α-Bromo Esters

In a round-bottom flask, zinc (30 mmol) activated by acetic acid, dry THF (15 mL), trifluoromethyl aldimine (25 mmol), and 2-bromo ester (30 mmol) were added under an argon atmosphere. The suspension was warmed to 50 or 60°C and stirred at the same temperature (6 h). The mixture was quenched with saturated aqueous NH_4_Cl (5 ml) and then extracted with Et_2_O (7 × 10 ml). The organic layers were washed with brine, dried over MgSO_4_, filtrated, and concentrated under reduced pressure to give the crude mixture that was purified using column chromatography (hexane/ethyl acetate or cyclohexane/ethyl acetate).

#### Racemic mixture of (S)-1-(4-methoxyphenyl)-4-(trifluoromethyl)azetidin-2-one (1)

Pale yellow oil (1,530 mg, 65%): The NMR data were in good agreement (Liu et al., 2013).

#### Racemic mixture of (S)-1-benzyl-4-(trifluoromethyl) azetidin-2-one (2)

Pale yellow oil (1223 mg, 68%): The NMR data were in good agreement (Trulli et al., 2018).

#### Racemic Mixture of (S)-1-(4-methoxybenzyl)-4-(trifluoromethyl)azetidin-2-one (3)

Pale yellow oil (1004 mg, 71%): ^1^H NMR (600 MHz, CDCl_3_) δ = 7.18 (d, *J* = 8.5 Hz, 2H, Ar), 6.88 (d, *J* = 8.6 Hz, 2H, Ar), 4.77 (d, *J* = 15.0 Hz, 1H, C*H*HPh), 3.92 (d, *J* = 15.0 Hz, 1H, C*H*HPh), 3.84–3.76 (m, 4H, C*H*CF_3_, OC*H*_3_), 3.08 (dd, *J* = 14.9, 5.3 Hz, 1H, CH*H*CHCF_3_), 3.02 (dd, *J* = 14.9, 1.4 Hz, 1H, C*H*HCHCF_3_). ^13^C NMR (151 MHz, CDCl_3_) δ = 165.29 (s, *C* = O), 159.59 (s, Ar), 129.97 (s, Ar), 126.75 (s, Ar), 124.58 (q, *J* = 279.3 Hz, *C*F_3_), 114.45 (s, Ar), 55.39 (s, O*C*H_3_), 49.59 (q, *J* = 35.0 Hz, *C*HCF_3_), 45.27 (s, *C*H_2_Ar), 38.60 (s, *C*CHCF_3_). ^19^F NMR (565 MHz, CDCl_3_) δ = −75.29 (d, *J* = 5.7 *Hz*). HRMS (ESI) calcd for C_12_H_12_F_3_NO_2_ ([M+H^+^]): 260.0898, found: 260.0890.

#### Racemic mixture of (3S,4S)-1-(4-methoxyphenyl)-3-methyl-4-(trifluoromethyl)azetidin-2-one (cis-9)

Pale yellow oil (353 mg, 25%): The NMR data were in good agreement (Thi et al., 2016).

#### Racemic mixture of (3R,4S)-1-(4-methoxyphenyl)-3-methyl-4-(trifluoromethyl)azetidin-2-one (trans-9)

Pale yellow oil (692 mg, 49%): The NMR data were in good agreement (Decamps et al., 2014).

#### Racemic Mixture of (3S,4S)-1-(4-methoxyphenyl)-3-phenyl-4-(trifluoromethyl)azetidin-2-one (cis-18)

Pale yellow oil (609 mg, 34%): ^1^H NMR (600 MHz, CDCl_3_) δ = 7.46 (d, *J* = 8.9 Hz, 2H, Ar), 7.42–7.32 (m, 5H, Ph), 6.93 (d, *J* = 9.0 Hz, 2H, Ar), 4.94 (d, *J* = 6.1 Hz, 1H, *H*CPh), 4.80 (p, *J* = 6.0 Hz, 1H, C*H*CF_3_), 3.81 (s, 3H, OC*H*_3_). ^13^C NMR (151 MHz, CDCl_3_) δ = 164.56 (s, N*C* = O), 157.31 (s, Ar), 130.06 (s, Ph), 129.84 (s, Ph), 129.62 (s, Ph), 128.71 (s, Ph), 128.63 (s, Ar), 123.76 (q, *J* = 281.3 Hz, *C*F_3_), 119.97 (s, Ar), 114.57 (s, Ar), 57.19 (q, *J* = 32.4 Hz, *C*HCF_3_), 56.66 (s, *C*HCHCF_3_), 55.62 (s, O*C*H_3_). ^19^F NMR (565 MHz, CDCl_3_) δ = −68.05 (d, *J* = 5.8 *Hz*). HRMS (ESI) calcd for C_17_H_14_F_3_NO_2_ ([M+H^+^]): 322.1055, found: 322.1039.

#### Racemic Mixture of (3R,4S)-1-(4-methoxyphenyl)-3-phenyl-4-(trifluoromethyl)azetidin-2-one (trans-18)

Pale yellow oil (771 mg, 43%): ^1^H NMR (600 MHz, CDCl_3_) δ = 7.52–7.28 (m, 7H, Ph, Ar), 6.92 (d, *J* = 8.9 Hz, 2H, Ar), 4.60 (br s, 1H, *H*CPh), 4.50–4.43 (m, 1H, C*H*CF_3_), 3.81 (s, 3H, OC*H*_3_). ^13^C NMR (151 MHz, CDCl_3_) δ = 163.82 (s, N*C* = O), 157.35 (s, Ar), 132.75 (s, Ph), 129.72 (s, Ph), 129.38 (s, Ph), 128.62 (s, Ar), 127.48 (s, Ph), 124.34 (q, *J* = 280.6 Hz, *C*F_3_), 119.86 (s, Ar), 114.62 (s, Ar), 59.44 (q, *J* = 32.4 Hz, *C*HCF_3_), 56.25 (d, *J* = 1.2 Hz, *C*HCHCF_3_), 55.65 (s, O*C*H_3_). ^19^F NMR (565 MHz, CDCl_3_) δ = −72.96 (d, *J* = 5.8 *Hz*). HRMS (ESI) calcd for C_17_H_14_F_3_NO_2_ ([M+H^+^]): 322.1055, found: 322.1060.

### General Procedure of the Reactions of 4-CF_3_ β-lactams With Electrophiles

In a round-bottom flask, dry THF (2 ml) was cooled to −25°C and then LiHMDS (1.0 M in THF, 0.75 mmol) was added dropwise under an argon atmosphere. Then, the solution of β-lactam (0.5 mmol) in dry THF (1 ml) was added. The suspension was stirred at the same temperature for 30 min and then the electrophile (1 or 0.6 mmol) was added dropwise (*p*-BrPhCHO was dissolved in 1 ml of dry THF). Then, the reaction mixture was stirred at the same temperature for 2 h and left overnight at room temperature. Then, the reaction mixture was cooled to 0°C and carefully quenched by dropwise addition of saturated aqueous NH_4_Cl (1 ml), and then extracted with Et_2_O (2 × 5 ml). The organic layers were washed with brine, dried over MgSO_4_, filtrated, and concentrated under reduced pressure and purified using column chromatography (hexane/ethyl acetate or cyclohexane/ethyl acetate).

#### Racemic mixture of 3S,4S)-3-((R)-hydroxy(phenyl) methyl)-1-(4-methoxyphenyl)-4-(trifluoromethyl) azetidin-2-one (4a) and (3S,4S)-3-((S)-hydroxy (phenyl)methyl)-1-(4-methoxyphenyl)-4-(trifluoromethyl)azetidin-2-one (4a and 4b)

Pale yellow oil (145 mg, 83%). The NMR data were in good agreement (Decamps et al., 2014).

#### Racemic mixture of (3S,4S)-3-((R)-1-benzyl-3-((S)-hydroxy(phenyl)methyl)-4-(trifluoromethyl)azetidin-2-one and (3S,4S)-3-((S)-1-benzyl-3-((S)-hydroxy (phenyl)methyl)-4-(trifluoromethyl)azetidin-2-one (5a and 5b)

Pale yellow oil (95 mg, 57%): The NMR data were in good agreement (Decamps et al., 2014).

#### Racemic mixture of (3S,4S)-3-((R)-hydroxy(phenyl)methyl)-1-(4-methoxybenzyl)-4-(trifluoromethyl)azetidin-2-one and (3S,4S)-3-((S)-hydroxy(phenyl)methyl)-1-(4-methoxybenzyl)-4-(trifluoromethyl)azetidin-2-one (6a and 6b)

Pale yellow oil (109 mg, 60%): The NMR data were in good agreement (Decamps et al., 2014).

#### Racemic mixture of (3S,4S)-3-((R)-(4-bromophenyl)(hydroxy)methyl)-1-(4-methoxyphenyl)-4-(trifluoromethyl)azetidin-2-one and (3S,4S)-3-((S)-(4-bromophenyl)(hydroxy)methyl)-1-(4-methoxyphenyl)-4-(trifluoromethyl)azetidin-2-one (7a and 7b)

Pale yellow oil (116 mg, 54%): The NMR data were in good agreement (Decamps et al., 2014).

#### Racemic mixture of (3S,4S)-3-((R)-hydroxy(4-methoxyphenyl)methyl)-1-(4-methoxyphenyl)-3-methyl-4-(trifluoromethyl)azetidin-2-one and (3S,4S)-3-((S)-hydroxy(4-methoxyphenyl)methyl)-1-(4-methoxyphenyl)-3-methyl-4-(trifluoromethyl)azetidin-2-one (8a and 8b)

Pale yellow oil (109 mg, 57%): ^1^H NMR (600 MHz, CDCl_3_) δ = 7.45 (d, *J* = 8.4 Hz), 7.36 (d, *J* = 8.6 Hz), 7.34–7.28 (m), 6.95–6.85 (m), 5.30 (br s), 5.08 (br d, *J* = 4.1 Hz), 4.68–4.60 (m), 4.40 (br s), 3.84–3.77 (m), 3.75 (br s), 3.64 (br s). ^13^C NMR (151 MHz, CDCl_3_) δ = 164.52, 163.75, 159.94, 159.52, 157.23, 157.22, 132.62, 131.97, 129.69 (d, *J* = 7.1 Hz), 128.78, 128.16, 126.57, 124.37 (q, *J* = 280.2 Hz, *C*F_3_), 124.31 (q, *J* = 280.1 Hz, *C*F_3_), 119.98, 119.94, 114.49, 114.26, 114.21, 114.07, 71.34, 68.56, 65.13, 59.33, 58.16, 55.84, 55.60, 55.40, 54.48 (q, *J* = 34.7 Hz, *C*HCF_3_), 52.78 (q, *J* = 34.8 Hz, *C*HCF_3_). ^19^F NMR (565 MHz, CDCl_3_) δ = −72.98 (d, *J* = 4.7 *Hz*),−72.80 (d, *J* = 4.7 *Hz*). HRMS (ESI) calcd for C_19_H_18_F_3_NO_4_ ([M+Na^+^]): 404.1086, found: 404.1090.

#### Racemic mixture of (3R,4S)-3-methyl-1-(4-methoxyphenyl)-4-(trifluoromethyl)azetidin-2-one (9)

Pale yellow oil (19 mg, 15%): The NMR data were in good agreement (Decamps et al., 2014).

#### Racemic mixture of (3R,4S)-3-ethyl-1-(4-methoxyphenyl)-4-(trifluoromethyl)azetidin-2-one (10)

Pale yellow oil (17 mg, 12%): The NMR data were in good agreement (Gong and Kato, 2001).

#### Racemic mixture of (3R,4S)-3-allyl-1-(4-methoxyphenyl)-4-(trifluoromethyl)azetidin-2-one (11)

Pale yellow oil (37 mg, 26%): The NMR data were in good agreement (Decamps et al., 2014).

#### Racemic Mixture of (3R,4S)-1-(4-methoxyphenyl)-3-(prop-2-yn-1-yl)-4-(trifluoromethyl)azetidin-2-one (12)

Pale yellow oil (56 mg, 40%): ^1^H NMR (600 MHz, CDCl_3_) δ = 7.36 (d, *J* = 7.9 Hz, 2H, Ar), 6.89 (d, *J* = 7.8 Hz, 2H, Ar), 4.46 (br d, *J* = 4.9 Hz, 1H, C*H*CF_3_), 3.80 (s, 3H, OC*H*_3_), 3.53 (br t, *J* = 4.9 Hz, 1H, C*H*CHCF_3_), 2.76 (br d, *J* = 2.7 Hz, 2H, C*H*_2_C≡CH), 2.08 (br d, *J* = 1.4 Hz, 1H, C≡C*H*). ^13^C NMR (151 MHz, CDCl_3_) δ = 163.81 (s, *C* = O), 157.37 (s, Ar), 129.59 (s, Ar), 124.32 (q, *J* = 280.3 Hz, *C*F_3_), 120.02 (s, Ar), 114.58 (s, Ar), 77.95 (s, *C*≡CH), 71.96 (s, C≡*C*H), 56.09 (q, *J* = 34.8 Hz, *C*HCF_3_), 55.64 (s, O*C*H_3_), 49.93 (s, *C*HCHCF_3_), 17.17 (s, *C*H_2_C≡CH). ^19^F NMR (565 NMR (565 MHz, CDCl_3_) δ = −72.97 (d, *J* = 5.0 *Hz*). HRMS (ESI) calcd for ([M+H^+^]): 284.0898, found: 284.0894.

#### Racemic Mixture of Ethyl (3R,4S)-1-(4-methoxyphenyl)-2-oxo-4-(trifluoromethyl)azetidine-3-carboxylate (13)

Pale yellow oil (74 mg, 47%): ^1^H NMR (600 MHz, CDCl_3_) δ = 7.35 (d, *J* = 8.9 Hz, 2H, Ar), 6.90 (d, *J* = 8.9 Hz, 2H, Ar), 4.86 (dq, *J* = 5.1, 2.2 Hz, 1H, C*H*CF_3_), 4.30 (dq, *J* = 7.1, 1.1 Hz, 2H, OC*H*_2_CH_3_), 4.21 (d, *J* = 2.1 Hz, 1H, C*H*CHCF_3_), 3.80 (s, 3H, OC*H*_3_), 1.34 (t, *J* = 7.1 Hz, 3H, OCH_2_C*H*_3_). ^13^C NMR (151 MHz, CDCl_3_) δ = 164.77 (s, N*C* = O), 157.72 (s, O*C* = O), 157.63 (s, Ar), 129.32 (s, Ar), 123.78 (q, *J* = 280.4 Hz, *C*F_3_), 120.05 (s, Ar), 114.62 (s, Ar), 62.95 (s, O*C*H_2_CH_3_), 55.89 (s, *C*HCHCF_3_), 55.64 (s, O*C*H_3_), 54.26 (q, *J* = 35.5 Hz, *C*HCF_3_), 14.21 (s, OCH_2_*C*H_3_). ^19^F NMR (565 MHz, CDCl_3_) δ = −73.15 (d, *J* = 6.1 *Hz*). HRMS (ESI) calcd for C_14_H_14_F_3_NO_4_ ([M+H^+^]): 318.095318, found: 318.0944.

#### Racemic Mixture of (S)-1-(4-methoxyphenyl)-3,3-dimethyl-4-(trifluoromethyl)azetidin-2-one (14)

Pale yellow oil (117 mg, 86%): ^1^H NMR (600 MHz, CDCl_3_) δ = 7.36 (d, *J* = 8.9 Hz, 2H, Ar), 6.88 (d, *J* = 8.9 Hz, 2H, Ar), 4.16 (q, *J* = 6.4 Hz, 1H, C*H*CF_3_), 3.79 (s, 3H, OC*H*_3_), 1.48 (s, 3H, CC*H*_3_), 1.43 (s, 3H, CC*H*_3_). ^13^C NMR (151 MHz, CDCl_3_) δ = 170.03 (s, *C* = O), 157.11 (s, Ar), 130.00 (s, Ar), 124.35 (q, *J* = 280.9 Hz, *C*F_3_), 119.91 (s, Ar), 114.51 (s, Ar), 62.59 (q, *J* = 32.7 Hz, *C*HCF_3_), 55.59 (s, O*C*H_3_), 54.02 (s, *C*CHCF_3_), 23.38 (s, C*C*H_3_), 16.99 (q, *J* = 2.4 Hz, C*C*H_3_). ^19^F NMR (565 MHz, CDCl_3_) δ = −68.19 (d, *J* = 6.0 *Hz*). HRMS (ESI) calcd for C_13_H_14_F_3_NO_2_ ([M+H^+^]): 274.1055, found: 274.1062.

#### Racemic Mixture of (S)-3-diethyl-1-(4-methoxyphenyl)-4-(trifluoromethyl)azetidin-2-one (15)

Pale yellow oil (49 mg, 33%): ^1^H NMR (600 MHz, CDCl_3_) δ = 7.34 (d, *J* = 7.8 Hz, 2H, Ar), 6.89 (d, *J* = 7.8 Hz, 2H, Ar), 4.17 (q, *J* = 5.6 Hz, 1H, C*H*CF_3_), 3.79 (s, 3H, OC*H*_3_), 2.06 (dq, *J* = 15.0, 7.8 Hz, 1H, C*H*HCH_3_), 1.97 (dq, *J* = 14.9, 7.6 Hz, 1H, CH*H*CH_3_), 1.87–1.72 (m, 2H, C*H*_2_CH_3_), 1.12 (t, *J* = 7.1 Hz, 3H, CH_2_C*H*_3_), 1.01 (t, *J* = 7.0 Hz, 3H, CH_2_C*H*_3_). ^13^C NMR (151 MHz, CDCl_3_) δ = 169.83 (s, *C* = O), 157.13 (s, Ar), 129.83 (s, Ar), 124.53 (q, *J* = 281.0 Hz, *C*F_3_), 120.17 (s, Ar), 114.51 (s, Ar), 62.23 (s, *C*CHCF_3_), 60.93 (q, *J* = 32.9 Hz, *C*HCF_3_), 55.64 (s, O*C*H_3_), 24.92 (s, *C*H_2_CH_3_), 20.62 (d, *J* = 2.1 Hz, *C*H_2_CH_3_), 8.84 (s, CH_2_*C*H_3_), 8.58 (s, CH_2_*C*H_3_). ^19^F NMR (565 MHz, CDCl_3_) δ = −66.86 (d, *J* = 5.8 *Hz*). HRMS (ESI) calcd for C_15_H_18_F_3_NO_2_ ([M+H^+^]): 302.1368, found: 302.1368.

#### Racemic Mixture of (S)-3,3-diallyl-1-(4-methoxyphenyl)-4-(trifluoromethyl)azetidin-2-one (16)

Pale yellow oil (69 mg, 43%): ^1^H NMR (600 MHz, CDCl_3_) δ = 7.32 (d, *J* = 8.8 Hz, 2H, Ar), 6.89 (d, *J* = 8.8 Hz, 2H, Ar), 6.03 (ddt, *J* = 16.0, 9.3, 5.7 Hz, 1H, *H*C = CH_2_), 5.74 (dddd, *J* = 16.3, 9.9, 8.3, 6.1 Hz, 1H, *H*C = CH_2_), 5.29–5.14 (m, 4H, 2 × HC = C*H*_2_), 4.29 (q, *J* = 6.5 Hz, 1H, C*H*CF_3_), 3.79 (s, 3H, OC*H*_3_), 2.82 (dd, *J* = 14.3, 4.8 Hz, 1H, C*H*HCH = CH_2_), 2.60–2.44 (m, 3H, CH*H*CH = CH_2_ and C*HH*CH = CH_2_). ^13^C NMR (151 MHz, CDCl_3_) δ = 168.36 (s, *C* = O), 157.31 (s, Ar), 132.45 (s, H*C* = CH_2_), 131.78 (s, H*C* = CH_2_), 129.40 (s, Ar), 124.52 (q, *J* = 281.1 Hz, *C*F_3_), 120.67 (s, HC = *C*H_2_), 120.40 (s, Ar), 119.40 (s, HC = *C*H_2_), 114.54 (s, Ar), 59.80 (s, *C*CHCF_3_), 59.41 (q, *J* = 33.3 Hz, *C*HCF_3_), 55.64 (s, O*C*H_3_), 37.39 (s, *C*H_2_CH = CH_2_), 34.04 (q, *J* = 2.5 Hz, *C*H_2_CH = CH_2_). ^19^F NMR (565 MHz, CDCl_3_) δ = −66.89 (d, *J* = 6.5 *Hz*). HRMS (ESI) calcd for C_17_H_18_F_3_NO_2_ ([M+Na^+^]): 348.1187, found: 348.1184.

#### Racemic Mixture of Diethyl (S)-1-(4-methoxyphenyl)-2-oxo-4-(trifluoromethyl)azetidine-3,3-dicarboxylate (17)

Pale yellow oil (68 mg, 35%): ^1^H NMR (600 MHz, CDCl_3_) δ = 7.35 (d, *J* = 8.9 Hz, 2H, Ar), 6.91 (d, *J* = 9.0 Hz, 2H, Ar), 5.22 (q, *J* = 6.0 Hz, 1H, C*H*CF_3_), 4.43–4.27 (m, 4H, 2 × OC*H*_2_CH_3_), 3.80 (s, 3H, OC*H*_3_), 1.34 (t, *J* = 7.1 Hz, 3H, OCH_2_C*H*_3_), 1.32 (t, *J* = 7.1 Hz, 3H, OCH_2_C*H*_3_). ^13^C NMR (151 MHz, CDCl_3_) δ = 163.00 (s, N*C* = O), 162.14 (s, O*C* = O), 158.04 (s, Ar), 156.10 (s, O*C* = O), 128.58 (s, Ar), 123.07 (q, *J* = 281.5 Hz, *C*F_3_), 121.07 (s, Ar), 114.62 (s, Ar), 70.75 (s, *C*CHCF_3_), 64.06 (s, O*C*H_2_CH_3_), 63.34 (s, O*C*H_2_CH_3_), 58.64 (q, *J* = 35.1 Hz, *C*HCF_3_), 55.66 (s, O*C*H_3_), 14.07 (s, OCH_2_*C*H_3_), 13.85 (s, OCH_2_*C*H_3_). ^19^F NMR (565 MHz, CDCl_3_) δ = −69.36 (d, *J* = 6.1 *Hz*). HRMS (ESI) calcd for C_17_H_18_F_3_NO_6_ ([M+Na^+^]): 412.0984, found: 412.0982.

#### Racemic Mixture of (3R, 4S)-3-ethyl-1-(4-methoxyphenyl)-3-methyl-4-(trifluoromethyl)azetidin-2-one (19)

Pale yellow oil (75 mg, 52%): ^1^H NMR (600 MHz, CDCl_3_) δ = 7.36 (d, *J* = 8.9 Hz, 2H, Ar), 6.89 (d, *J* = 9.0 Hz, 2H, Ar), 4.19 (q, *J* = 6.5 Hz, 1H, C*H*CF_3_), 3.79 (s, 3H, OC*H*_3_), 1.88–1.75 (m, 2H, C*H*_2_CH_3_), 1.42 (s, 3H, CC*H*_3_), 1.04 (t, *J* = 7.5 Hz, 3H, CH_2_C*H*_3_). ^13^C NMR (151 MHz, CDCl_3_) δ = 169.87 (s, *C* = O), 157.12 (s, Ar), 129.87 (s, Ar), 124.49 (q, *J* = 280.9 Hz, *C*F_3_), 119.95 (s, Ar), 114.52 (s, Ar), 60.44 (q, *J* = 32.8 Hz, *C*HCF_3_), 58.26 (s, *C*CHCF_3_), 55.61 (s, O*C*H_3_), 29.56 (s, *C*H_2_CH_3_), 14.51 (q, *J* = 2.6 Hz, C*C*H_3_), 8.91 (s, CH_2_*C*H_3_). ^19^F NMR (565 MHz, CDCl_3_) δ = −67.73 (d, *J* = 6.0 *Hz*). HRMS (ESI) calcd for C_14_H_16_F_3_NO_2_ ([M+H^+^]): 288.1211, found: 288.1214.

#### Racemic Mixture of (3R,4S)-3-allyl-1-(4-methoxyphenyl)-3-methyl-4-(trifluoromethyl)azetidin-2-one (20)

Pale yellow oil (106 mg, 71%): ^1^H NMR (600 MHz, CDCl_3_) δ = 7.34 (d, *J* = 8.9 Hz, 2H, Ar), 6.88 (d, *J* = 8.9 Hz, 2H, Ar), 5.83–5.73 (m, 1H, *H*C = CH_2_), 5.27–5.18 (m, 2H, HC = C*H*_2_), 4.25 (q, *J* = 6.5 Hz, 1H, C*H*CF_3_), 3.78 (s, 3H, OC*H*_3_), 2.53 (dd, *J* = 14.1, 6.5 Hz, 1H, C*H*HCH = CH_2_), 2.46 (dd, *J* = 14.1, 8.1 Hz, 1H, CH*H*CH = CH_2_), 1.44 (s, 3H, CC*H*_3_). ^13^C NMR (151 MHz, CDCl_3_) δ = 169.11 (s, *C* = O), 157.18 (s, Ar), 131.90 (s, H*C* = CH_2_), 129.60 (s, Ar), 124.48 (q, *J* = 280.9 Hz, *C*F_3_), 120.37 (s, HC = *C*H_2_), 120.09 (s, Ar), 114.48 (s, Ar), 59.66 (q, *J* = 32.9 Hz, *C*HCF_3_), 57.09 (s, *C*CHCF_3_), 55.55 (s, O*C*H_3_), 40.84 (s, *C*H_2_CH = CH_2_), 15.32 (q, *J* = 2.5 Hz, C*C*H_3_). ^19^F NMR (565 MHz, CDCl_3_) δ = −67.68 (d, *J* = 6.0 *Hz*). HRMS (ESI) calcd for C_15_H_16_F_3_NO_2_ ([M+H^+^]): 300.1211, found: 300.1216.

#### Racemic Mixture of (3R, 4S)-1-(4-methoxyphenyl)-3-methyl-3-(prop-2-yn-1-yl)-4-(trifluoromethyl)azetidin-2-one (21)

Pale yellow oil (96 mg, 65%): ^1^H NMR (600 MHz, CDCl_3_) δ = 7.36 (d, *J* = 8.9 Hz, 2H, Ar), 6.89 (d, *J* = 9.0 Hz, 2H, Ar), 4.51 (q, *J* = 6.4 Hz, 1H, C*H*CF_3_), 3.79 (s, 3H, OC*H*_3_), 2.68 (dd, 1H, *J* = 17.1, 2.4, C*H*HC≡CH), 2.59 (dd, 1H, *J* = 17.1, 2.4, CH*H*C≡CH), 2.09 (t, *J* = 2.4 Hz, 1H, C≡C*H*), 1.50 (s, CC*H*_3_). ^13^C NMR (151 MHz, CDCl_3_) δ = 167.83 (s, *C* = O), 157.39 (s, Ar), 129.33 (s, Ar), 124.30 (q, *J* = 280.8 Hz, *C*F_3_), 120.47 (s, Ar), 114.54 (s, Ar), 78.15 (s, *C*≡CH), 72.26 (s, C≡*C*H), 60.18 (q, *J* = 33.2 Hz, *C*HCF_3_), 56.42 (s, *C*CHCF_3_), 55.61 (s, O*C*H_3_), 26.26 (s, *C*H_2_C≡CH), 15.27 (q, *J* = 2.4 Hz, C*C*H_3_). ^19^F NMR (565 MHz, CDCl_3_) δ = −67.88 (d, *J* = 6.0 *Hz*). HRMS (ESI) calcd C_15_H_14_F_3_NO_2_ ([M+H^+^]): 298.1055, found: 298.1057.

#### Racemic Mixture of Ethyl (3R,4S)-1-(4-methoxyphenyl)-3-methyl-2-oxo-4-(trifluoromethyl)azetidine-3-carboxylate (22)

Pale yellow oil (98 mg, 45%): ^1^H NMR (600 MHz, CDCl_3_) δ = 7.36 (d, *J* = 8.9 Hz, 2H, Ar), 6.90 (d, *J* = 8.9 Hz, 2H, Ar), 4.96 (q, *J* = 6.3 Hz, 1H, C*H*CF_3_), 4.33–4.23 (m, 2H, OC*H*_2_CH_3_), 3.80 (s, 3H, OC*H*_3_), 1.72 (s, 3H, CC*H*_3_), 1.31 (t, *J* = 7.1 Hz, 3H, OCH_2_C*H*_3_). ^13^C NMR (151 MHz, CDCl_3_) δ = 168.17 (s, N*C* = O), 162.59 (s, O*C* = O), 157.65 (s, Ar), 129.19 (s, Ar), 123.81 (q, *J* = 281.1 Hz, *C*F_3_), 120.58 (s, Ar), 114.60 (s, Ar), 62.96 (s, O*C*H_2_CH_3_), 62.45 (s, *C*CHCF_3_), 58.83 (q, *J* = 33.7 Hz, *C*HCF_3_), 55.63 (s, O*C*H_3_), 14.17 (s, OCH_2_*C*H_3_), 13.02 (q, *J* = 2.5 Hz, C*C*H_3_). ^19^F NMR (565 MHz, CDCl_3_) δ = −67.70 (d, *J* = 6.3 *Hz*). HRMS (ESI) calcd for C_15_H_16_F_3_NO_4_ ([M+H^+^]): 332.1110, found: 332.1102.

#### Racemic mixture of diastereomer A of (3S,4S)-3-(hydroxy(phenyl)methyl)-1-(4-methoxyphenyl)-3-methyl-4-(trifluoromethyl)azetidin-2-one (23a)

Pale yellow oil (86 mg, 44%): ^1^H NMR (600 MHz, CDCl_3_) δ = 7.47 (d, *J* = 7.2 Hz, 2H, Ph), 7.37–7.27 (m, 3H, Ph), 7.14 (d, *J* = 8.8 Hz, 2H, Ar), 6.80 (d, *J* = 8.9 Hz, 2H, Ar), 4.85 (s, 1H, *H*COH), 4.59 (q, *J* = 6.5 Hz, 1H, C*H*CF_3_), 3.75 (s, 3H, OC*H*_3_), 2.87 (br d, *J* = 15.9 Hz, 1H, O*H*), 1.39 (s, 3H CC*H*_3_). ^13^C NMR (151 MHz, CDCl_3_) δ = 168.01 (s, N*C* = O), 157.36 (s, Ar), 138.90 (s, Ph), 129.03 (s, Ar), 128.74 (s, Ph), 128.55 (s, Ph), 127.45 (s, Ph), 124.54 (q, *J* = 280.9 Hz, *C*F_3_), 120.90 (s, Ar), 114.37 (s, Ar), 76.63 (s, H*C*OH), 62.33 (s, *C*CHCF_3_), 58.03 (q, *J* = 32.8 Hz, *C*HCF_3_), 55.51 (s, O*C*H_3_), 13.16 (d, *J* = 1.8 Hz, C*C*H_3_). ^19^F NMR (565 MHz, CDCl_3_) δ = −67.43 (d, *J* = 5.8 *Hz*). HRMS (ESI) calcd for C_19_H_18_F_3_NO_3_ ([M+H^+^]): 366.1317, found: 366.1318.

#### Racemic mixture of diastereomer B of (3S,4S)-3-(hydroxy(phenyl)methyl)-1-(4-methoxyphenyl)-3-methyl-4-(trifluoromethyl)azetidin-2-one (23b)

Pale yellow oil (43 mg, 23%): ^1^H NMR (600 MHz, CDCl_3_) δ = 7.41–7.30 (m, 7H, Ph, Ar), 6.87 (d, *J* = 9.1 Hz, 2H, Ar), 4.98 (q, *J* = 6.7 Hz, 1H, C*H*CF_3_), 4.87 (s, 1H, *H*COH), 3.79 (s, 3H, OC*H*_3_), 3.80 (br s, 1H, O*H*), 1.24 (s, 3H CC*H*_3_). ^13^C NMR (151 MHz, CDCl_3_) δ = 169.42 (s, N*C* = O), 157.35 (s, Ar), 139.22 (s, Ph), 129.40 (s, Ar), 128.72 (s, Ph), 127.49 (s, Ph), 126.97 (s, Ph), 124.45 (q, *J* = 280.8 Hz, *C*F_3_), 120.74 (s, Ar), 114.50 (s, Ar), 74.80 (s, H*C*OH), 62.40 (s, *C*CHCF_3_), 56.37 (q, *J* = 33.0 Hz, *C*HCF_3_), 55.59 (s, O*C*H_3_), 13.46 (d, *J* = 1.9 Hz, C*C*H_3_). ^19^F NMR (565 MHz, CDCl_3_) δ = −67.01 (d, *J* = 6.3 *Hz*). HRMS (ESI) calcd for C_19_H_18_F_3_NO_3_ ([M+H^+^]): 366.1317, found: 366.1316.

#### Racemic mixture of diastereomer A of (3S,4S)-3-((4-bromophenyl)(hydroxy)methyl)-1-(4-methoxyphenyl)-3-methyl-4-(trifluoromethyl)azetidin-2-one (24a)

Pale yellow oil (70 mg, 32%): ^1^H NMR (600 MHz, CDCl_3_) δ = 7.49 (d, *J* = 8.4 Hz, 2H, 4-BrPh), 7.38 (d, *J* = 8.3 Hz, 2H, 4-BrPh), 7.21 (d, *J* = 8.9 Hz, 2H, 4-MeOPh), 6.85 (d, *J* = 8.9 Hz, 2H, 4-MeOPh), 4.87 (s, 1H, *H*COH), 4.59 (q, *J* = 6.5 Hz, 1H, C*H*CF_3_), 3.78 (s, 3H, OC*H*_3_), 2.49 (br s, 1H, O*H*), 1.37 (s, 3H CC*H*_3_). ^13^C NMR (151 MHz, CDCl_3_) δ = 167.60 (s, N*C* = O), 157.51 (s, 4-MeOPh), 137.84 (s, 4-BrPh), 131.83 (s, 4-BrPh), 129.33 (s, 4-BrPh), 129.09 (s, 4-MeOPh), 124.46 (q, *J* = 280.9 Hz, *C*F_3_), 123.01 (s, 4-BrPh), 120.75 (s, 4-MeOPh), 114.52 (s, 4-MeOPh), 76.36 (s, H*C*OH), 62.03 (s, *C*CHCF_3_), 58.17 (q, *J* = 32.9 Hz, *C*HCF_3_), 55.63 (s, O*C*H_3_), 13.09 (q, *J* = 2.6 Hz, C*C*H_3_). ^19^F NMR (565 MHz, CDCl_3_) δ = −67.40 (d, *J* = 5.9 *Hz*). HRMS (ESI) calcd for C_19_H_17_BrF_3_NO_3_ ([M+H^+^]): 444.0422 and 446.0402, found: 444.0404 and 446.0386.

#### Racemic mixture of diastereomer B of (3S,4S)-3-((4-bromophenyl)(hydroxy)methyl)-1-(4-methoxyphenyl)-3-methyl-4-(trifluoromethyl)azetidin-2-one (24b)

Pale yellow oil (87mg, 39%): ^1^H NMR (600 MHz, CDCl_3_) δ = 7.51 (d, *J* = 8.3 Hz, 2H, 4-BrPh), 7.21 (d, *J* = 8.2 Hz, 2H, 4-BrPh), 7.36 (d, *J* = 8.9 Hz, 2H, 4-MeOPh), 6.88 (d, *J* = 8.9 Hz, 2H, 4-MeOPh), 4.91 (q, *J* = 6.6 Hz, 1H, C*H*CF_3_), 4.86 (s, 1H, *H*COH), 3.80 (s, 3H, OC*H*_3_), 2.96 (br s, 1H, O*H*), 1.24 (s, 3H CC*H*_3_). ^13^C NMR (151 MHz, CDCl_3_) δ = 169.02 (s, N*C* = O), 157.49 (s, 4-MeOPh), 138.19 (s, 4-BrPh), 131.86 (s, 4-BrPh), 129.27 (s, 4-MeOPh), 128.62 (s, 4-BrPh), 124.35 (q, *J* = 280.6 Hz, *C*F_3_), 122.78 (s, 4-BrPh), 120.73 (s, 4-MeOPh), 114.56 (s, 4-MeOPh), 74.22 (s, H*C*OH), 62.24 (s, *C*CHCF_3_), 56.32 (q, *J* = 33.0 Hz, *C*HCF_3_), 55.64 (s, O*C*H_3_), 13.39 (d, *J* = 2.0 Hz, C*C*H_3_). ^19^F NMR (565 MHz, CDCl_3_) δ = −67.02 (d, *J* = 6.0 Hz). HRMS (ESI) calcd for C_19_H_17_BrF_3_NO_3_ ([M+H^+^]): 444.0422 and 446.0402, found: 444.0416 and 446.0399.

#### Racemic mixture of (3S,4S)-3-((R)-hydroxy(4-methoxyphenyl)methyl)-1-(4-methoxyphenyl)-3-methyl-4-(trifluoromethyl)azetidin-2-one and (3S,4S)-3-((S)-hydroxy(4-methoxyphenyl)methyl)-1-(4-methoxyphenyl)-3-methyl-4-(trifluoromethyl)azetidin-2-one (25a and 25b)

Pale yellow oil (102 mg, 52%): ^1^H NMR (600 MHz, CDCl_3_) δ = 7.43 (d, *J* = 8.6 Hz), 7.38 (d, *J* = 8.9 Hz), 7.27 (d, *J* = 6.9 Hz), 7.22 (d, *J* = 8.9 Hz), 7.00–6.82 (m), 4.95 (q, *J* = 6.8 Hz), 4.86 (br d, *J* = 5.6 Hz), 4.65–4.55 (m), 3.88–3.76 (m), 2.61 (br s), 2.41 (br s), 1.38 (s), 1.25 (s). ^13^C NMR (151 MHz, CDCl_3_) δ = 169.35, 167.98, 159.99, 159.94, 157.35, 131.25, 130.99, 129.52, 129.29, 128.86, 128.17, 124.60 (q, *J* = 280.9 Hz, *C*F_3_), 124.53 (q, *J* = 281.0 Hz, *C*F_3_), 120.75, 120.69, 114.51, 114.44, 114.10, 114.04, 76.59, 74.71, 65.19, 62.48, 62.45, 58.15 (q, *J* = 32.9 Hz, *C*HCF_3_), 56.45 (q, *J* = 32.9 Hz, *C*HCF_3_), 55.63, 55.60, 55.43, 13.47 (q, *J* = 2.0 Hz, C*C*H_3_), 13.18 (q, *J* = 2.3 Hz, C*C*H_3_). ^19^F NMR (565 MHz, CDCl_3_) δ = −67.41 (d, *J* = 6.1 *Hz*),−66.96 (d, *J* = 6.4 *Hz*). HRMS (ESI) calcd for C_20_H_20_F_3_NO_4_ ([M+Na^+^]): 418.1242, found: 418.1243.

#### Racemic Mixture of (3S,4S)-1-(4-methoxyphenyl)-3-methyl-3-phenyl-4-(trifluoromethyl)azetidin-2-one (26)

Pale yellow oil (122 mg, 73%): ^1^H NMR (600 MHz, CDCl_3_) δ = 7.54–7.31 (m, 7H, Ph, Ar), 6.93 (d, *J* = 9.0 Hz, 2H, Ar), 4.46 (q, *J* = 6.0 Hz, 1H, C*H*CF_3_), 3.81 (s, 3H, OC*H*_3_), 1.90 (s, 3H, C*H*_3_). ^13^C NMR (151 MHz, CDCl_3_) δ = 168.41 (s, *C* = O), 157.40 (s, Ar), 135.19 (s, Ph), 129.80 (s, Ph), 128.51 (s, Ph), 128.29 (s, Ar), 127.59 (s, Ph), 123.60 (q, *J* = 281.2 Hz, *C*F_3_), 120.50 (s, Ar), 114.53 (s, Ar), 64.41 (q, *J* = 32.0 Hz, *C*HCF_3_), 61.49 (s, *C*CHCF_3_), 55.61 (s, O*C*H_3_), 24.12 (s, C*C*H_3_). ^19^F NMR (565 MHz, CDCl_3_) δ = −68.39 (d, *J* = 5.7 *Hz*). HRMS (ESI) calcd for C_18_H_16_F_3_NO_2_ ([M+H^+^]): 336.1211, found: 336.1211.

#### Racemic Mixture of (3S,4S)-3-ethyl-1-(4-methoxyphenyl)-3-phenyl-4-(trifluoromethyl)azetidin-2-one (27)

Pale yellow oil (120 mg, 69%): ^1^H NMR (600 MHz, CDCl_3_) δ = 7.54–7.30 (m, 7H, Ph, Ar), 6.92 (d, *J* = 8.9 Hz, 2H, Ar), 4.45 (q, *J* = 5.8 Hz, 1H, C*H*CF_3_), 3.81 (s, 3H, OC*H*_3_), 2.37 (dq, *J* = 14.8, 7.4 Hz, 1H, C*H*HCH_3_), 2.27 (dq, *J* = 14.4, 7.3 Hz, 1H, CH*H*CH_3_), 0.98 (t, *J* = 7.3 Hz, 3H, CH_2_C*H*_3_). ^13^C NMR (151 MHz, CDCl_3_) δ = 167.77 (s, *C* = O), 157.38 (s, Ar), 134.23 (s, Ph), 129.63 (s, Ph), 128.52 (s, Ph), 128.20 (s, Ar), 127.88 (s, Ph), 123.86 (q, *J* = 281.3 Hz, *C*F_3_), 120.56 (s, Ar), 114.55 (s, Ar), 65.89 (s, *C*CHCF_3_), 62.57 (q, *J* = 31.8 Hz, *C*HCF_3_), 55.66 (s, O*C*H_3_), 31.34 (s, *C*H_2_CH_3_), 9.18 (s, CH_2_*C*H_3_). ^19^F NMR (565 MHz, CDCl_3_) δ = −68.08 (d, *J* = 5.6 *Hz*). HRMS (ESI) calcd for C_19_H_18_F_3_NO_2_ ([M+Na^+^]): 372.1187, found: 372.1192.

#### Racemic Mixture of (3S,4S)-3-allyl-1-(4-methoxyphenyl)-3-phenyl-4-(trifluoromethyl)azetidin-2-one (28)

Pale yellow oil (96 mg, 53%): ^1^H NMR (600 MHz, CDCl_3_) δ = 7.56–7.29 (m, 7H, Ph, Ar), 6.91 (d, *J* = 8.9 Hz, 2H, Ar), 5.84–5.73 (m, 1H, *H*C = CH_2_), 5.31 (br d, *J* = 17.0 Hz, 1H, HC = C*H*H), 5.20 (br d, *J* = 10.2 Hz, 1H, HC = CH*H*), 4.53 (q, *J* = 6.0 Hz, 1H, C*H*CF_3_), 3.81 (s, 3H, OC*H*_3_), 2.95 (d, *J* = 7.2 Hz, 2H, C*H*_2_CH = CH_2_). ^13^C NMR (151 MHz, CDCl_3_) δ = 167.12 (s, *C* = O), 157.53 (s, Ar), 134.64 (s, Ph), 131.87 (s, H*C* = CH_2_), 129.16 (s, Ph), 128.58 (s, Ph), 128.35 (s, Ar), 127.75 (s, Ph), 123.96 (q, *J* = 281.3 Hz, *C*F_3_), 121.17 (s, HC = *C*H_2_), 121.05 (s, Ar), 114.54 (s, Ar), 64.74 (s, *C*CHCF_3_), 61.07 (q, *J* = 32.0 Hz, *C*HCF_3_), 55.65 (s, O*C*H_3_), 41.72 (s, *C*H_2_CH = CH_2_). ^19^F NMR (565 MHz, CDCl_3_) δ = −67.92 (d, *J* = 5.6 *Hz*). HRMS (ESI) calcd for C_20_H_18_F_3_NO_2_ ([M+Na^+^]): 384.1187, found: 384.1193.

#### Racemic Mixture of (3S,4S)-1-(4-methoxyphenyl)-3-phenyl-3-(prop-2-yn-1-yl)-4-(trifluoromethyl)azetidin-2-one (29)

Pale yellow oil (84 mg, 47%): ^1^H NMR (600 MHz, CDCl_3_) δ = 7.55–7.32 (m, 7H, Ph, Ar), 6.92 (d, *J* = 9.0 Hz, 2H, Ar), 4.90 (q, *J* = 6.1 Hz, 1H, C*H*CF_3_), 3.81 (s, 3H, OC*H*_3_), 3.17 (dd, *J* = 17.3, 2.6, 1H, C*H*HC≡CH), 2.97 (dd, *J* = 17.3, 2.4, 1H, CH*H*C≡CH), 2.10 (t, *J* = 2.5 Hz, 1H, C≡C*H*). ^13^C NMR (151 MHz, CDCl_3_) δ = 166.17 (s, *C* = O), 157.66 (s, Ar), 133.69 (s, Ph), 128.91 (s, Ar), 128.78 (s, Ph), 128.73 (s, Ph), 127.62 (s, Ph), 123.78 (q, *J* = 280.9 Hz, *C*F_3_), 121.33 (s, Ar), Ar), 114.49 (s, Ar), 78.21 (s, *C*≡CH), 72.55 (s, C≡*C*H), 63.68 (s, *C*CHCF_3_), 61.39 (q, *J* = 32.4 Hz, *C*HCF_3_), 55.61 (s, O*C*H_3_), 26.86 (s, *C*H_2_C≡CH). ^19^F NMR (565 MHz, CDCl_3_) δ = −68.05 (d, *J* = 6.3 *Hz*). HRMS (ESI) calcd for C_20_H_16_F_3_NO_2_ ([M+Na^+^]): 382.1030 found: 382.1022.

#### Racemic Mixture of Ethyl (3R,4S)-1-(4-methoxyphenyl)-2-oxo-3-phenyl-4-(trifluoromethyl)azetidine-3-carboxylate (30)

Pale yellow oil (74 mg, 50%): ^1^H NMR (600 MHz, CDCl_3_) δ = 7.50–7.35 (m, 7H, Ph, Ar), 6.92 (d, *J* = 8.9 Hz, 2H, Ar), 5.39 (q, *J* = 5.8 Hz 1H, C*H*CF_3_), 4.34–4.20 (m, 2H, OC*H*_2_CH_3_), 3.81 (s, 3H, OC*H*_3_), 1.20 (t, *J* = 7.1 Hz, 3H, OCH_2_C*H*_3_). ^13^C NMR (151 MHz, CDCl_3_) δ = 167.50 (s, N*C* = O), 160.67 (s, O*C* = O), 157.79 (s, Ar), 130.49 (s, Ph), 129.17 (s, Ph), 129.05 (s, Ph), 128.81 (s, Ph), 128.39 (s, Ar), 123.26 (q, *J* = 281.3 Hz, *C*F_3_), 120.92 (s, Ar), 114.57 (s, Ar), 71.52 (s, *C*CHCF_3_), 63.36 (s, O*C*H_2_CH_3_), 60.36 (q, *J* = 32.6 Hz, *C*HCF_3_), 55.64 (s, O*C*H_3_), 13.99 (s, OCH_2_*C*H_3_). ^19^F NMR (565 MHz, CDCl_3_) δ = −68.00 (d, *J* = 5.5 *Hz*). HRMS (ESI) calcd for C_20_H_18_F_3_NO_4_ ([M+H^+^]): 394.1266, found: 394.1262.

### Procedure of the *N*-PMP deprotection of 4-CF_3_ β-lactams

A solution of β-lactam **14** (0.25 mmol) in MeCN (1.5 ml) was cooled to 0°C and then CAN (0.75 mmol) in water (1 ml) was added dropwise. The mixture was stirred at 0°C for AN additional 30 min. Then, water (5 ml) was added and the mixture was extracted with EtOAc (4 × 5 ml) and washed with 10% aqueous NaHCO_3_ (10 ml). The aqueous layer was extracted again with EtOAc (10 ml) and all organic layers were combined and washed successively with 10% NaHSO_3_ (2 × 5 ml), 10% NaHCO_3_ (5 ml), and brine (5 ml) and then dried over MgSO_4_. After filtration and evaporation of the solvent *in vacuo*, the crude product **31** was purified by column chromatography (cyclohexane/ethyl acetate; 25:75, v/v).

#### Racemic Mixture of (S)-3,3-dimethyl-4-(trifluoromethyl)azetidin-2-one (31)

Pale yellow oil (31 mg, 73%): ^1^H NMR (600 MHz, CDCl_3_) δ = 6.05 (br s, N*H*), 3.74 (q, *J* = 6.8 Hz, 1H, C*H*CF_3_), 1.44 (s, 3H, CC*H*_3_), 1.35 (s, 3H, CC*H*_3_). ^13^C NMR (151 MHz, CDCl_3_) δ = 172.87 (s, *C* = O), 124.44 (q, *J* = 278.9 Hz, *C*F_3_), 59.03 (q, *J* = 33.6 Hz, *C*HCF_3_), 56.05 (s, *C*CHCF_3_), 23.45 (s, C*C*H_3_), 16.79 (q, *J* = 2.4 Hz, C*C*H_3_). ^19^F NMR (565 MHz, CDCl_3_) δ = −72.20 (d, *J* = 6.5 *Hz*). HRMS (ESI) calcd for C_6_H_8_F_3_NO ([M+H^+^]): 168.0636, found: 168.0637.

## Conclusion

In conclusion, we developed a convenient and highly diastereoselective synthesis of C-3 mono- and disubstituted 4-CF_3_ β-lactams. We showed that the enolate of 4-CF_3_ β-lactams can be formed under basic conditions and then undergoes reaction with various electrophiles. These wide ranges of 4-CF_3_ β-lactams variously functionalized are excellent synthons for constructing products of interest or for incorporating them into bioactive molecules.

## Data Availability

All datasets generated for this study are included in the manuscript/Supplementary Files.

## Author Contributions

TC and BC carried out manuscript writing. MS carried out chemical synthesis, characterization, and manuscript writing. TM and MK contributed to manuscript writing and revision. BC, TC, and HK designed and managed the study. All authors listed have made substantial, direct, and intellectual contributions to the work, and approved it for publication.

### Conflict of Interest Statement

The reviewer AP-L declared a shared affiliation, with no collaboration, with one of the authors BC to the handling editor at time of review. The remaining authors declare that the research was conducted in the absence of any commercial or financial relationships that could be construed as a potential conflict of interest.
