# Supplementary material for: Direct Access to Substituted 4-CF3 β-Lactams at the C-3 Position
Source: Front Chem. 2019 Aug 6;7:526. doi: 10.3389/fchem.2019.00526 (PMC6691124; doi:10.3389/fchem.2019.00526)
Supplement: Supplementary file 1 [file Data_Sheet_1.PDF]

Supporting Information

Direct Access to Substituted 4-CF_3 β-Lactams in C-3 Position

Monika Skibińska[1,2], Marcin Kaźmierczak[1,3], Thierry Milcent[2], Tomasz Cytlak*[1,3], Henryk Koroniak[1] and Benoit Crousse*[2]

(1) Faculty of Chemistry, Adam Mickiewicz University, Uniwersytetu Poznańskiego 8, 61-614 Poznań, Poland,
(2) Faculté de Pharmacie, UMR 8076 CNRS, BioCIS, Univ. Paris-Sud, Univ. Paris-Saclay, 92290, Châtenay-Malabry, France
(3) Centre for Advanced Technologies, Adam Mickiewicz University, Uniwersytetu Poznańskiego 10, 61-614 Poznań, Poland
Corresponding authors: cytlak@amu.edu.pl and benoit.crousse@u-psud.fr

Table of Contents

1. **1H, ^{13}C, ^{19}F Spectra of Compounds…………………………………………………………S2**
2. **Example of 2D HOESY ^1H-^{19}F NMR spectrum……………………………………………….S39**

1. ^{1}H, ^{13}C, ^{19}F NMR Spectra of Compounds

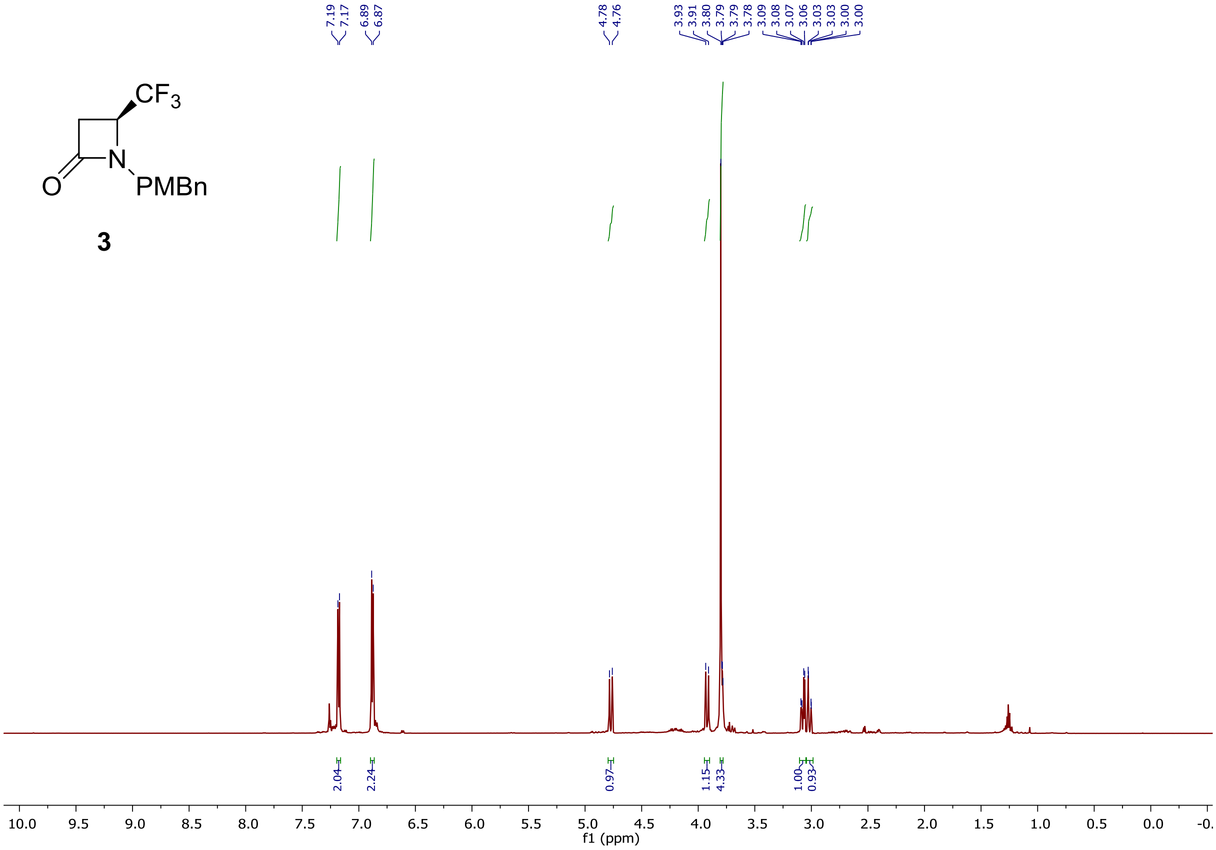

^{1}H NMR of 3

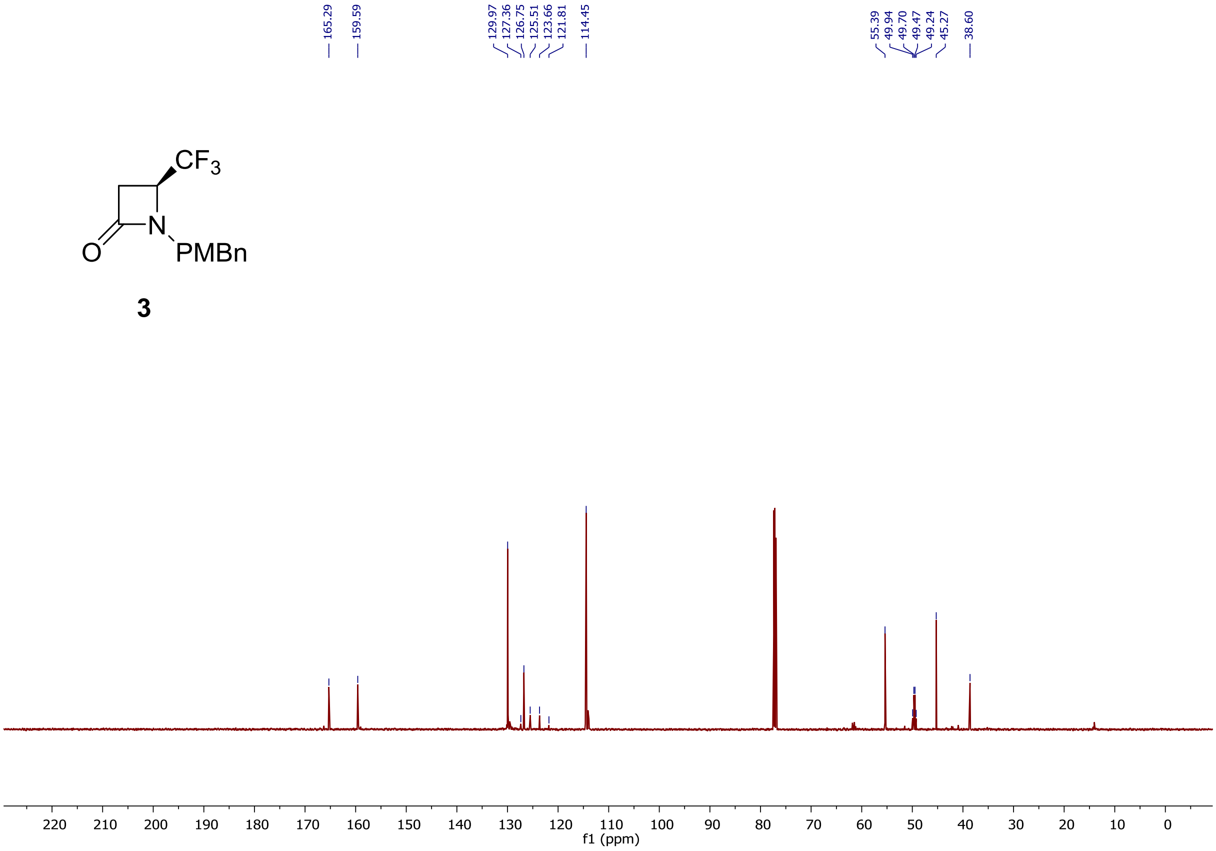

^{13}C NMR of 3

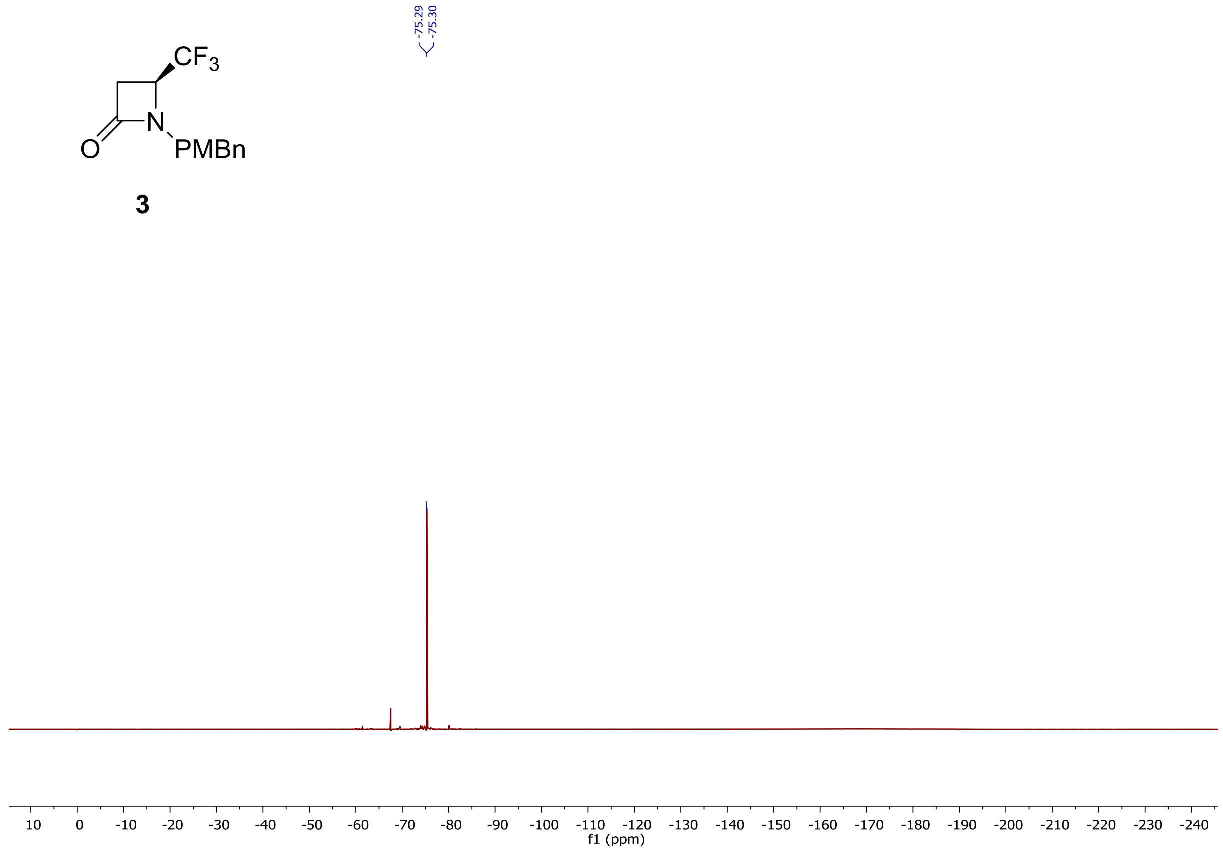

^{19}F NMR of 3

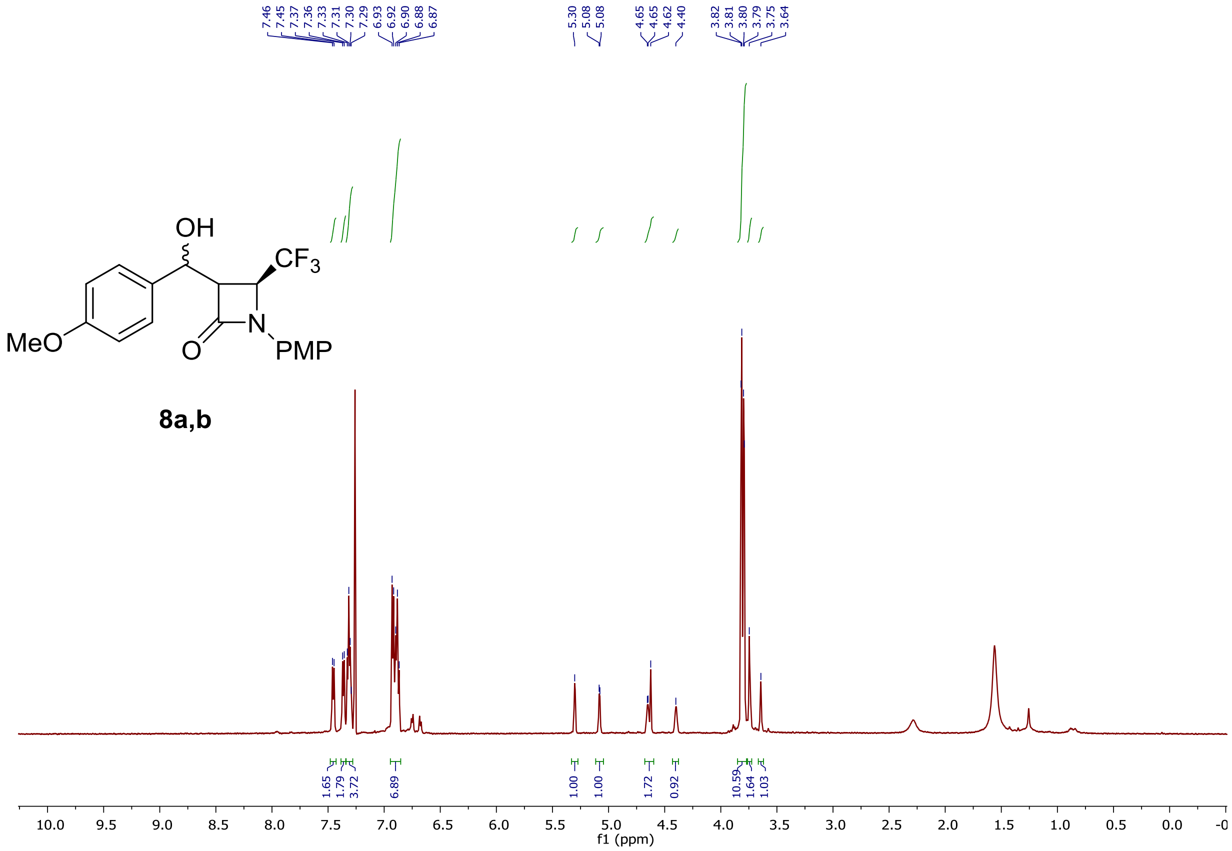

^{1}H NMR of 8a and 8b

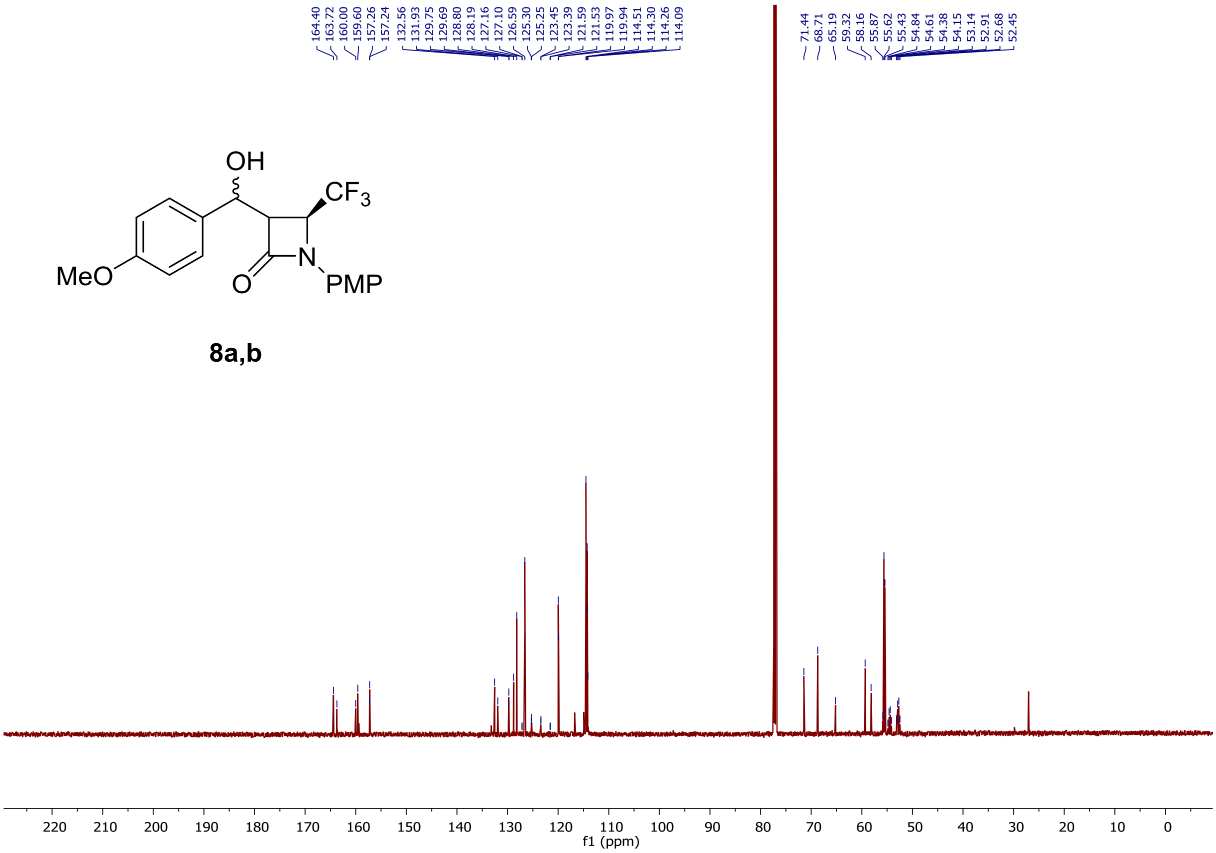

^{13}C NMR of 8a and 8b

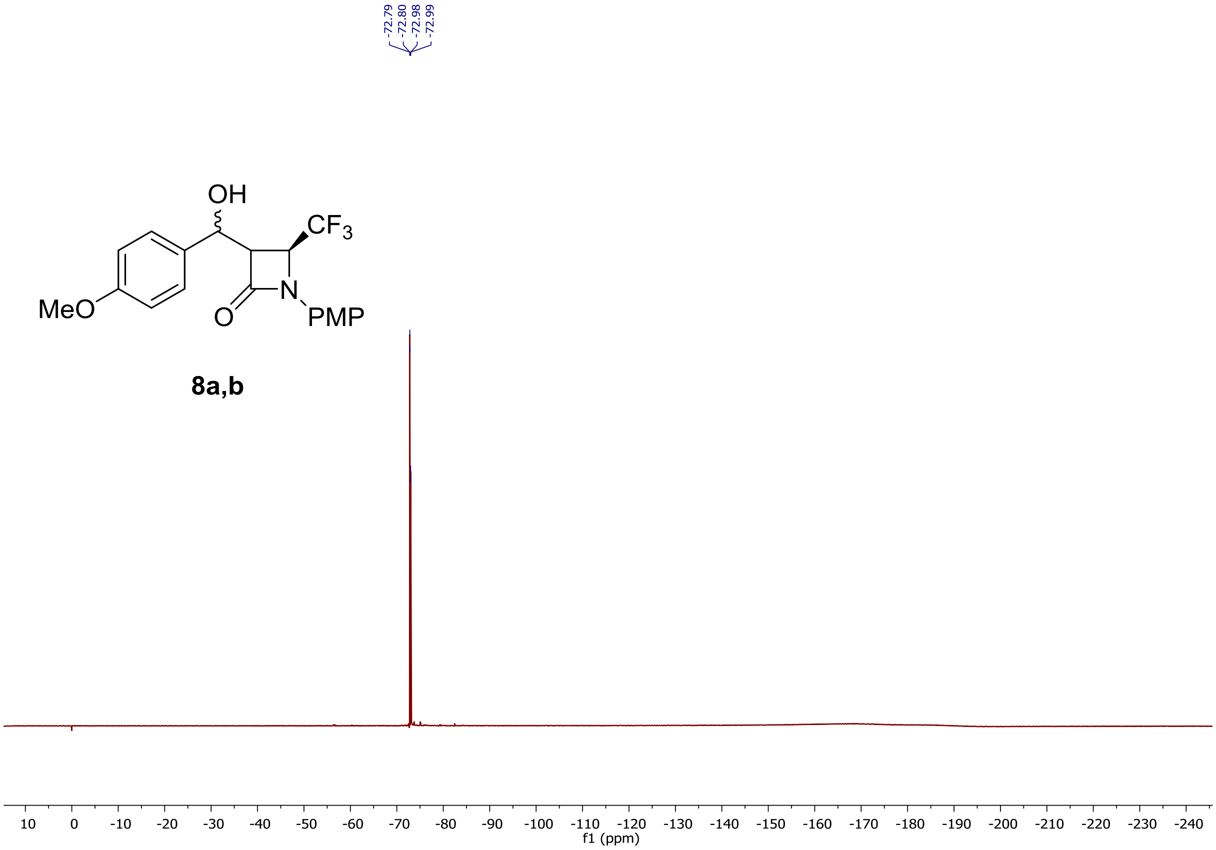

^{19}F NMR of 8a and 8b

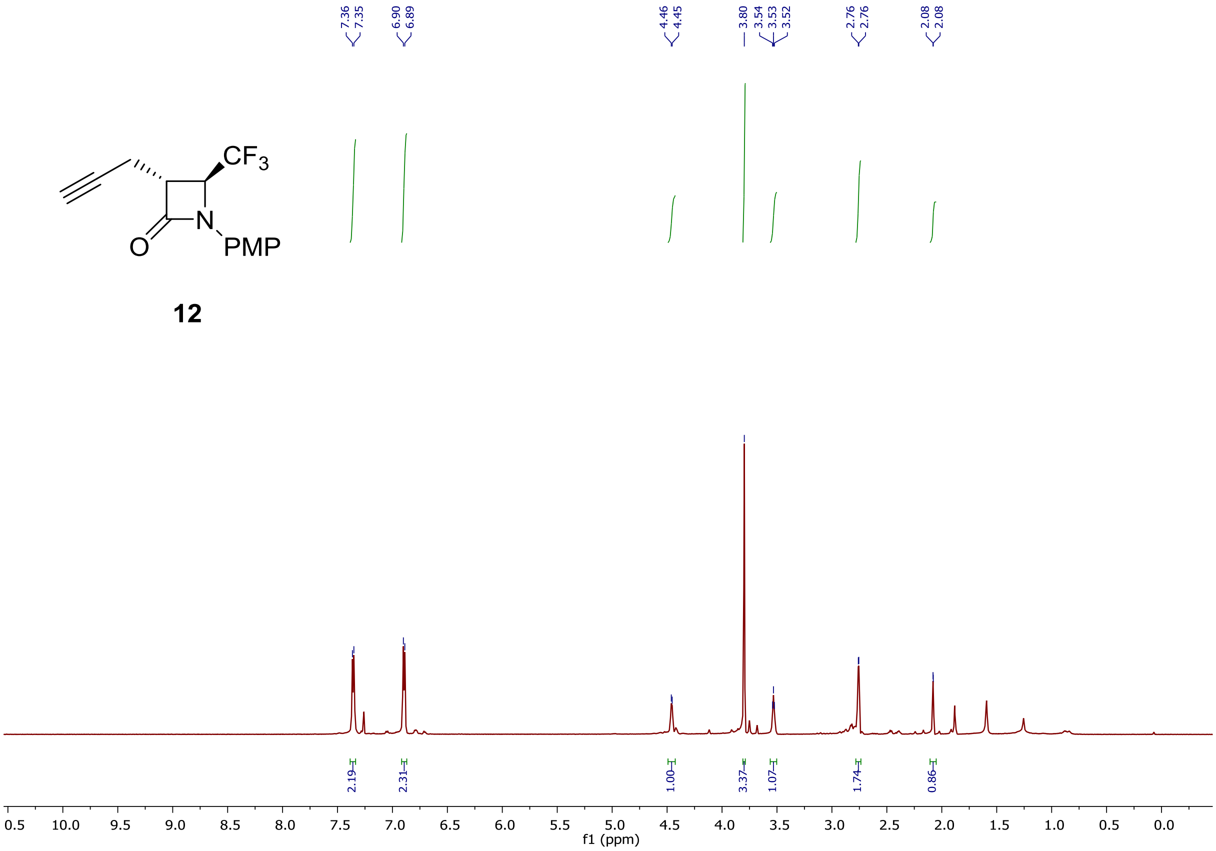

^{1}H NMR of 12

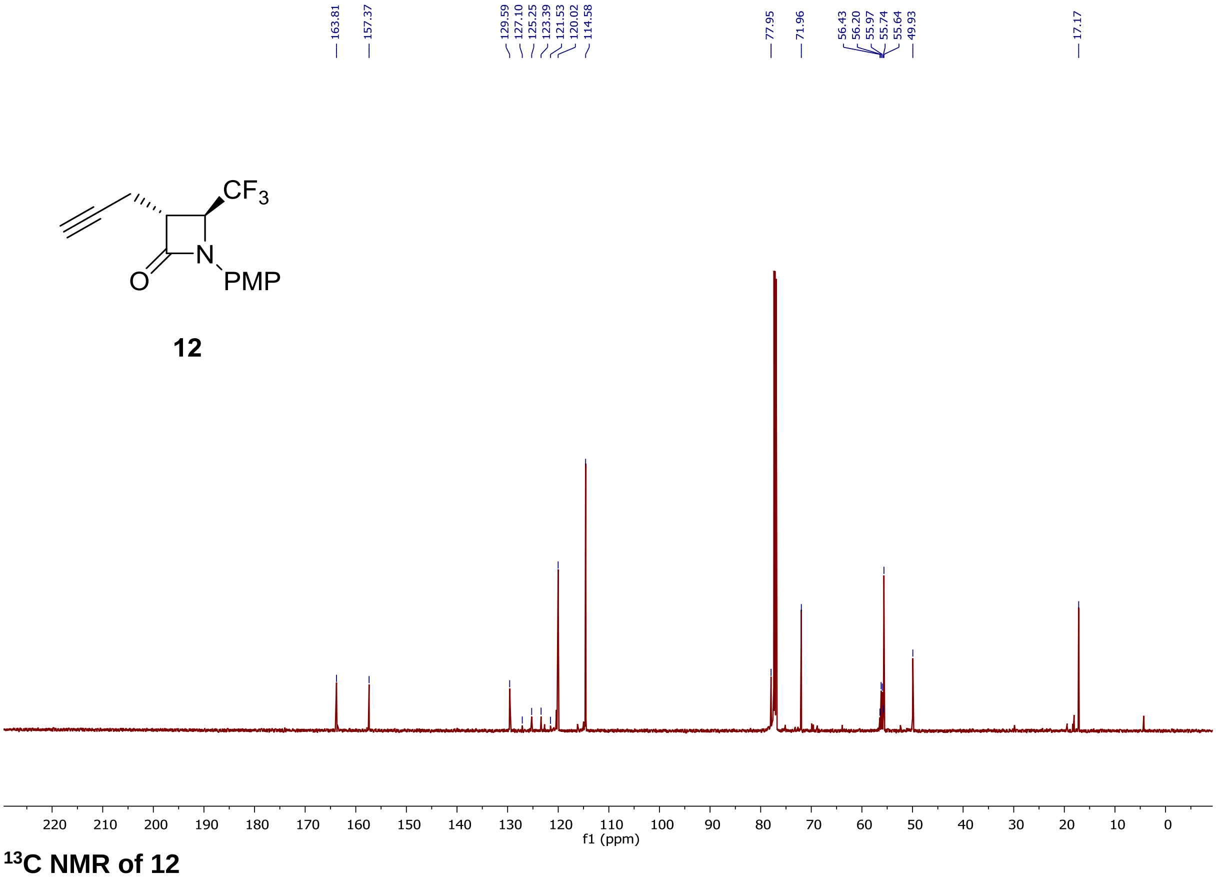

^{13}C NMR of 12

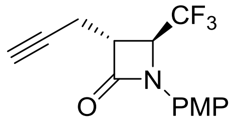

12

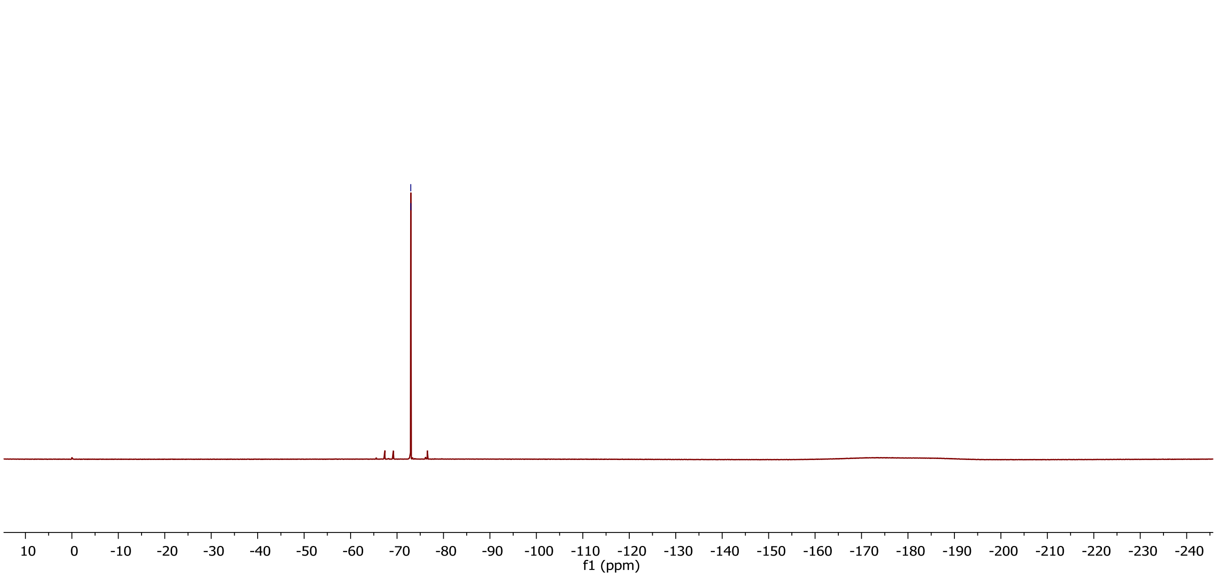

^{19}F NMR of 12

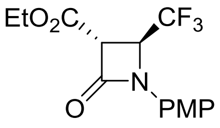

13

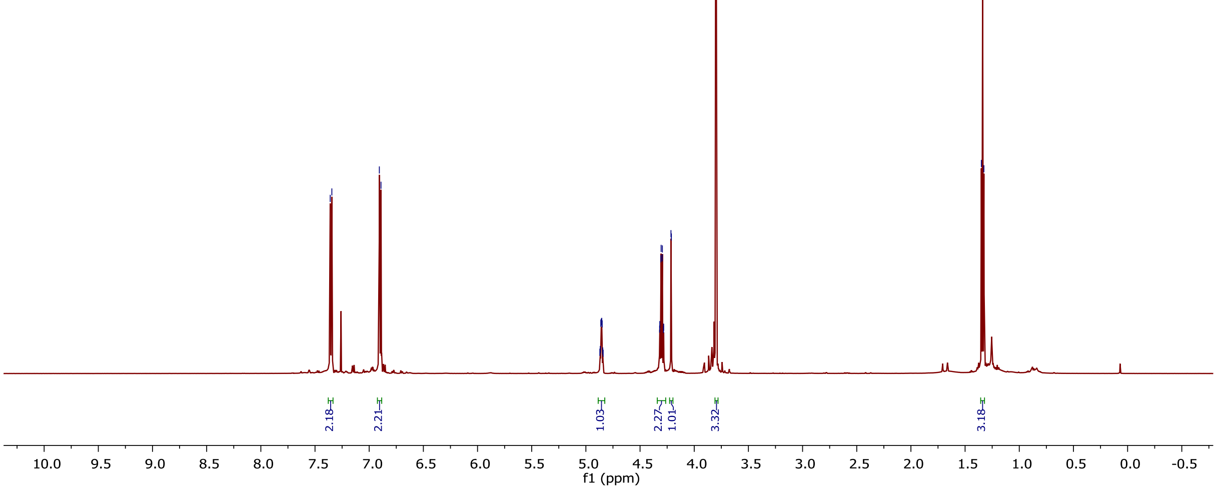

^{1}H NMR of 13

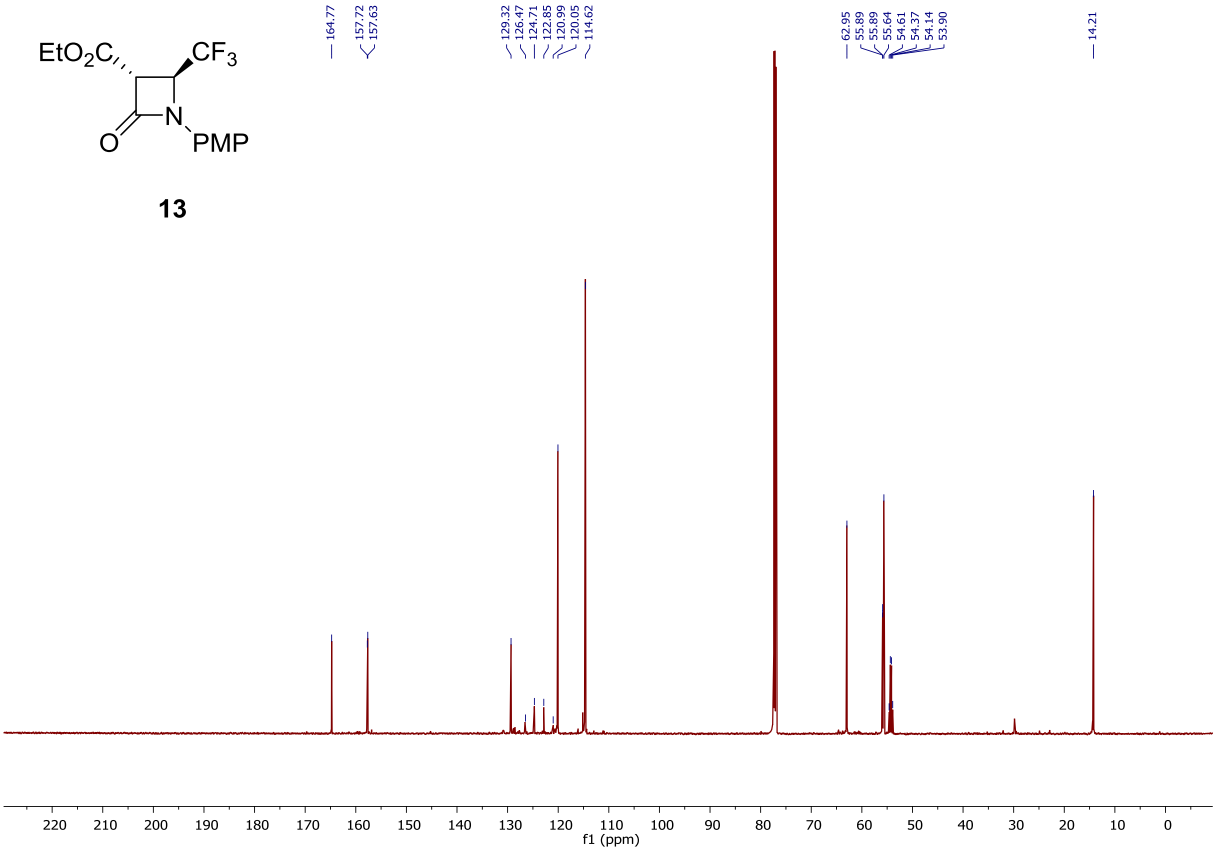

^{13}C NMR of 13

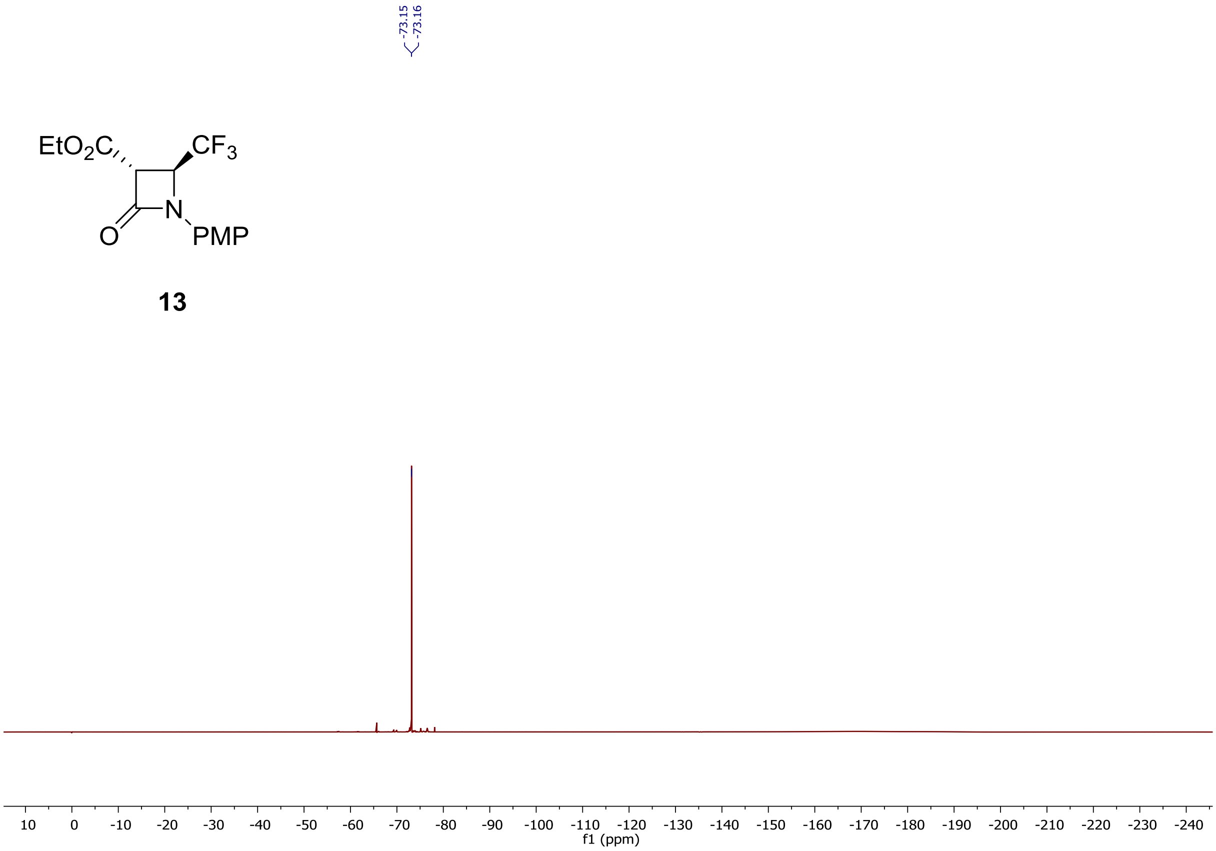

^{19}F NMR of 13

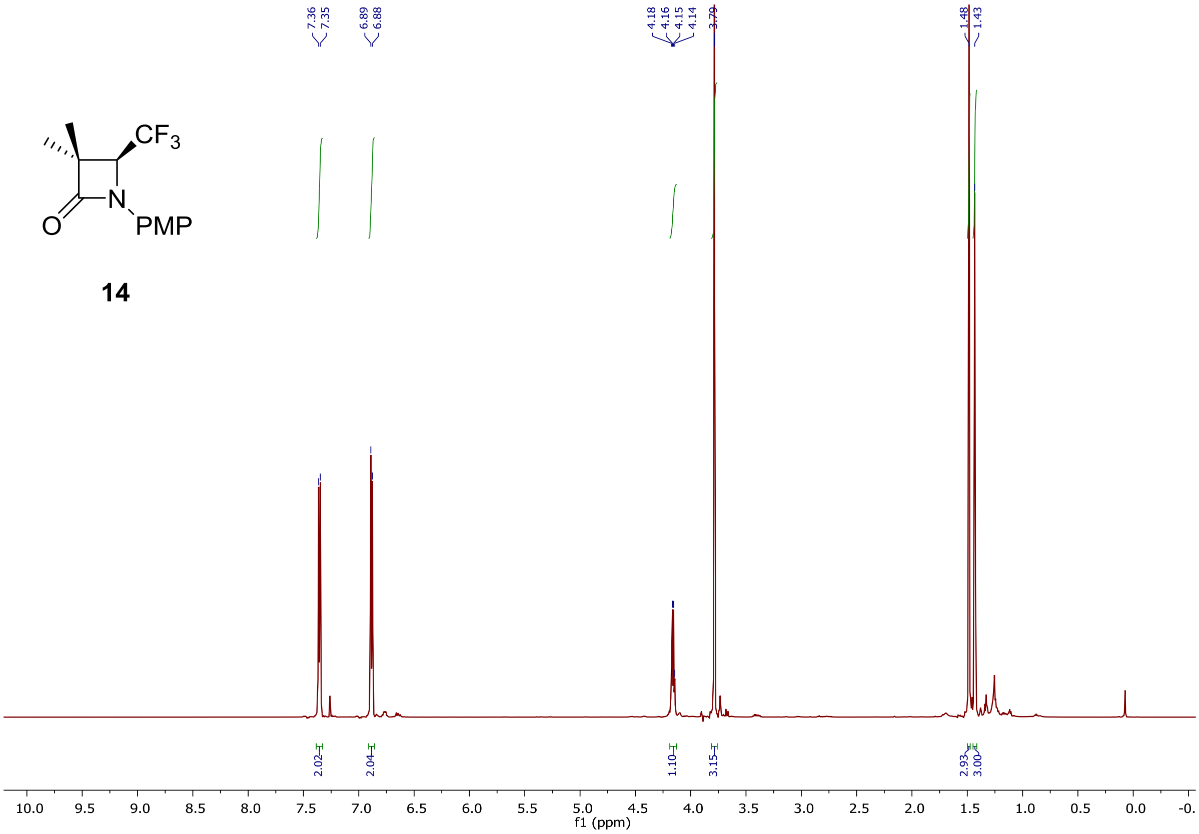

^{1}H NMR of 14

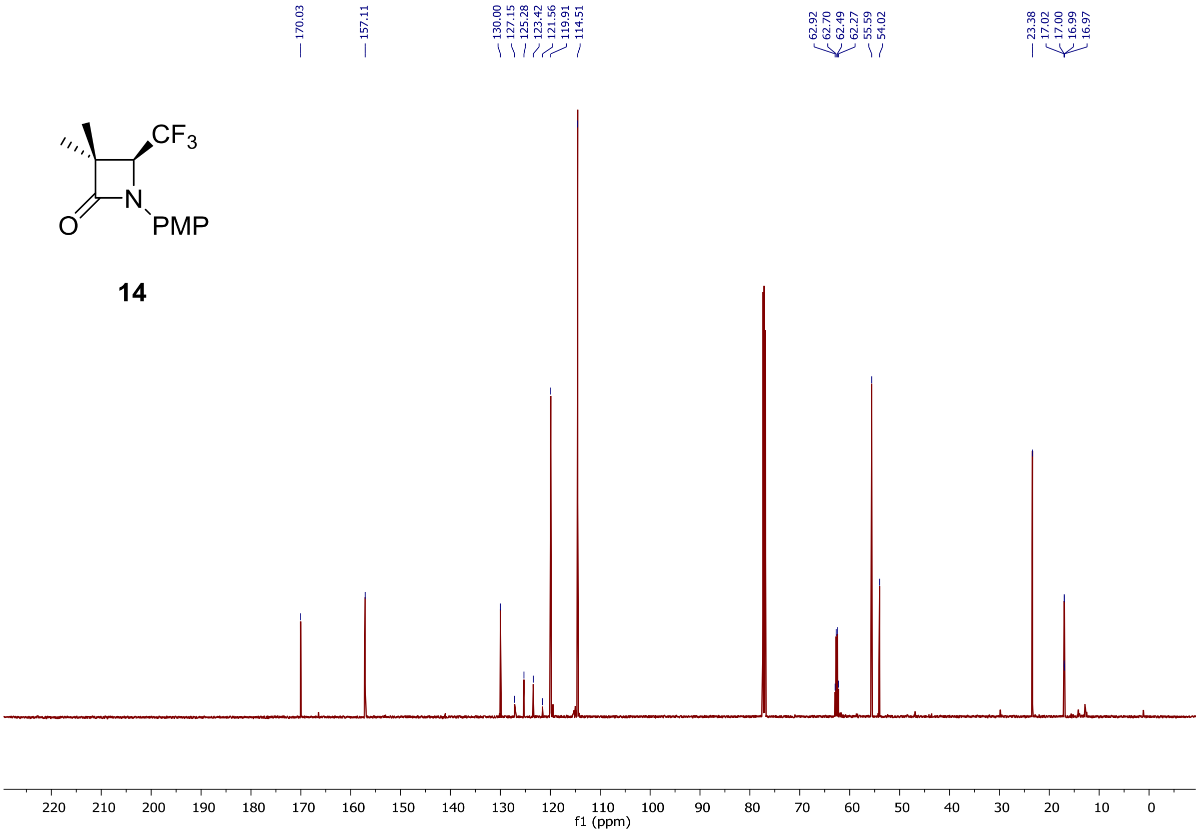

^{13}C NMR of 14

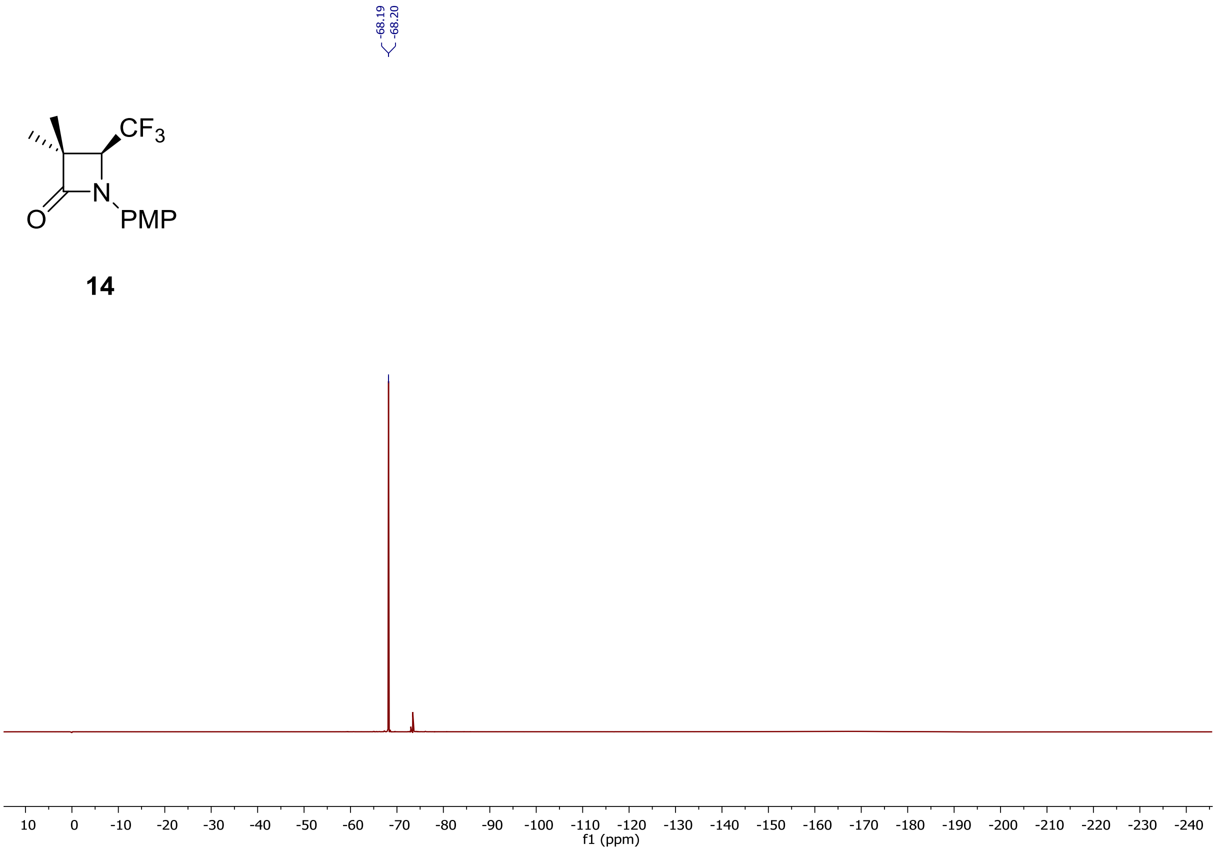

^{19}F NMR of 14

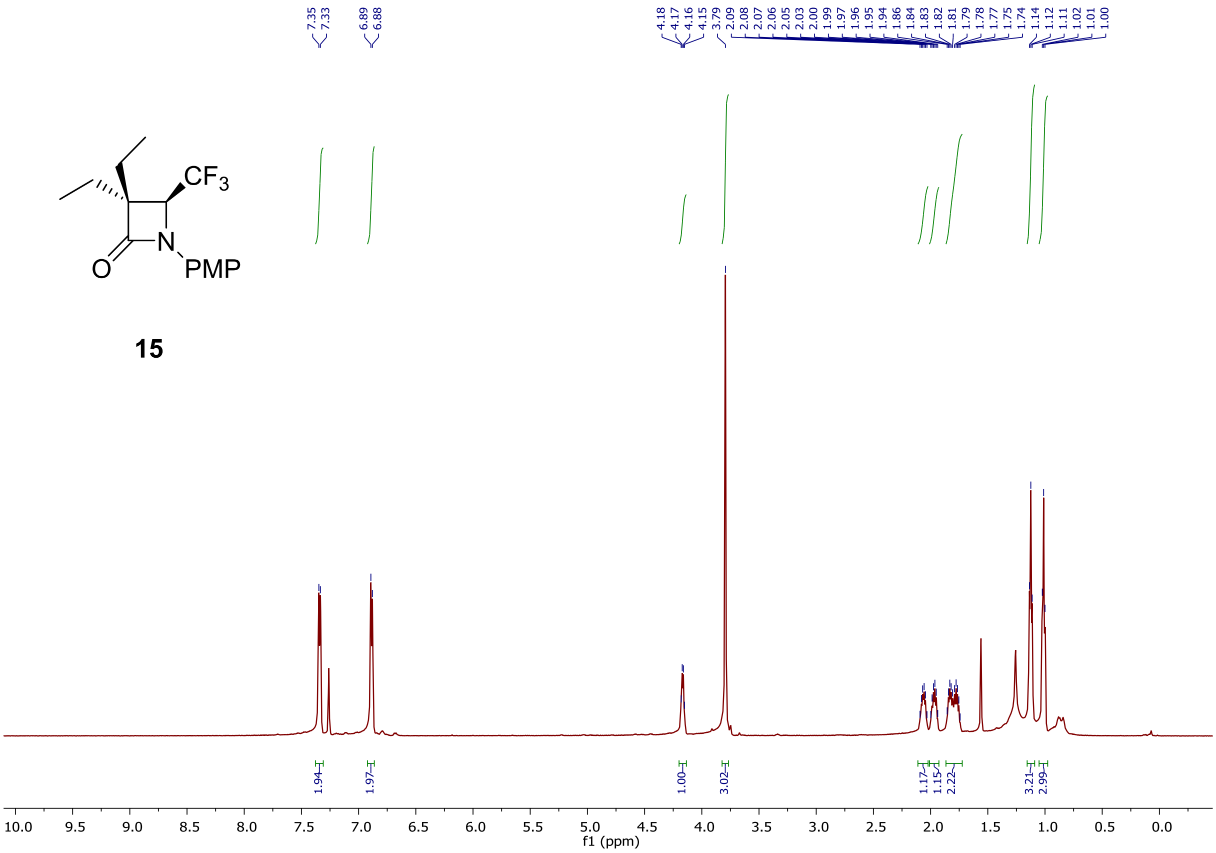

^{1}H NMR of 15

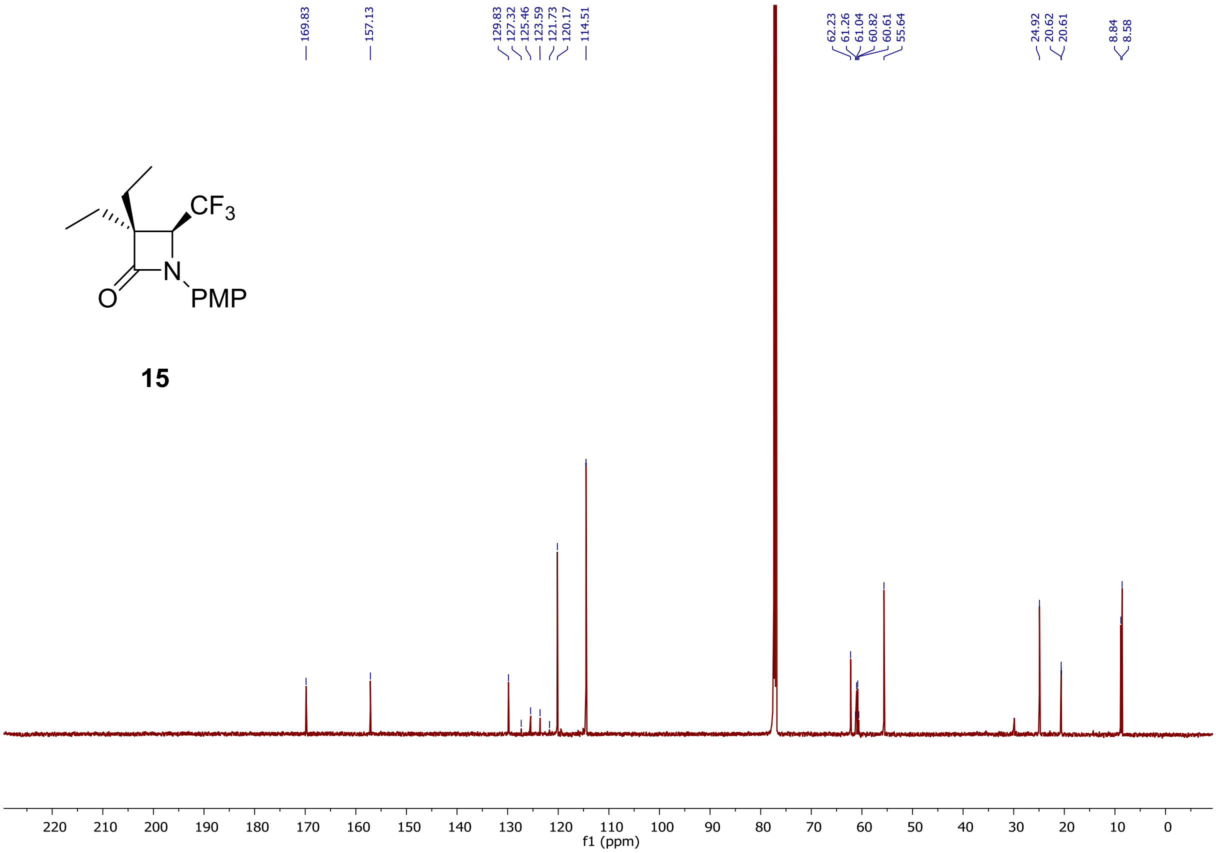

^{13}C NMR of 15

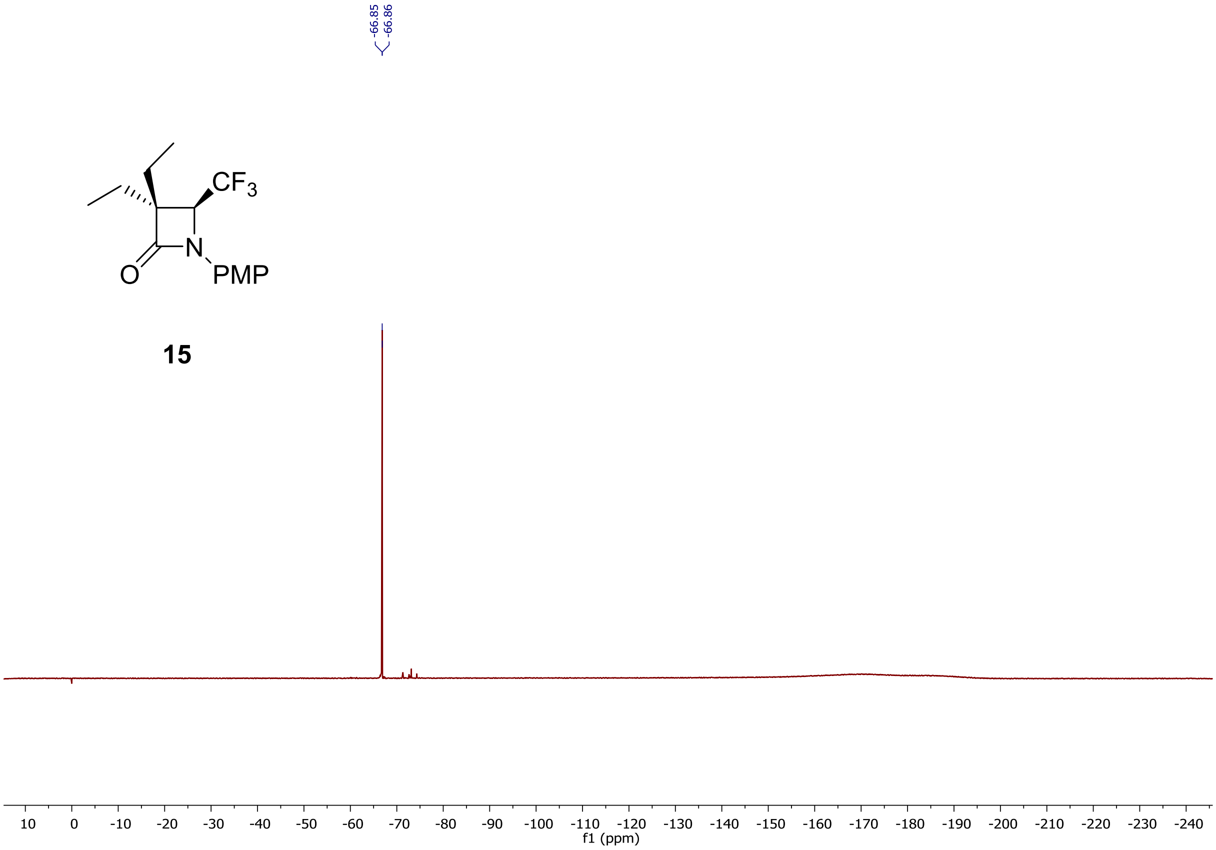

^{19}F NMR of 15

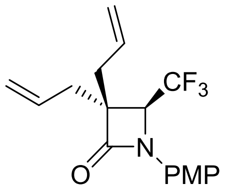

16

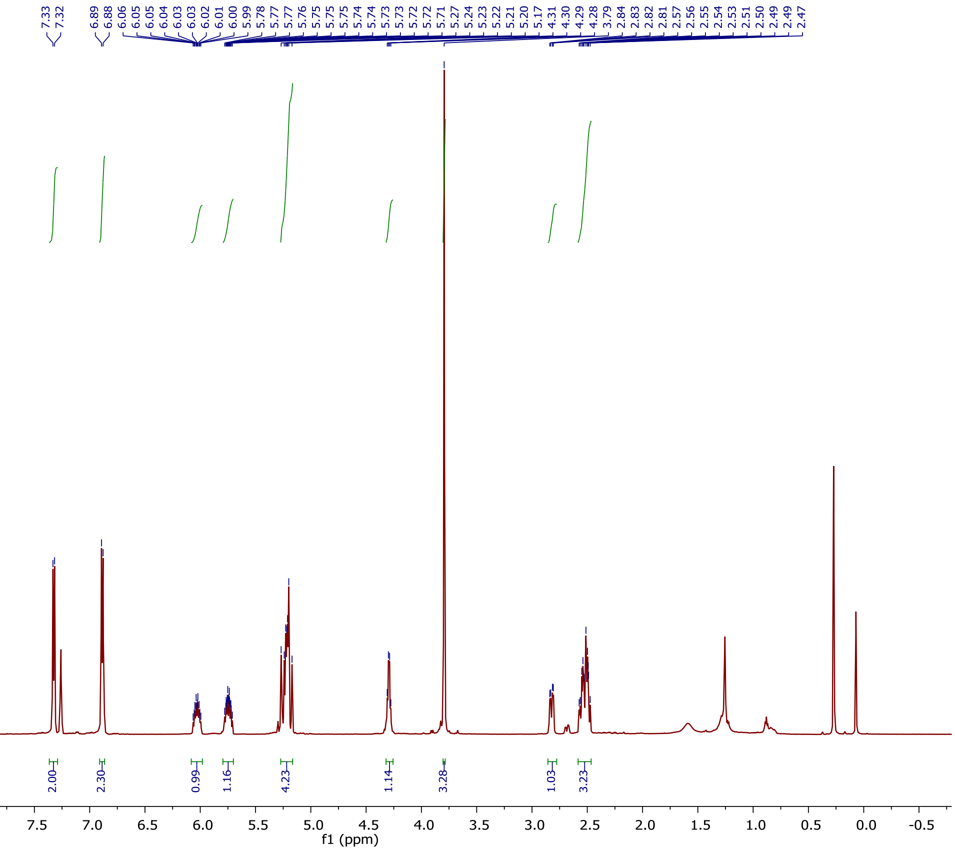

^{1}H NMR of 16

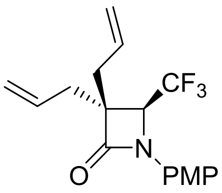

16

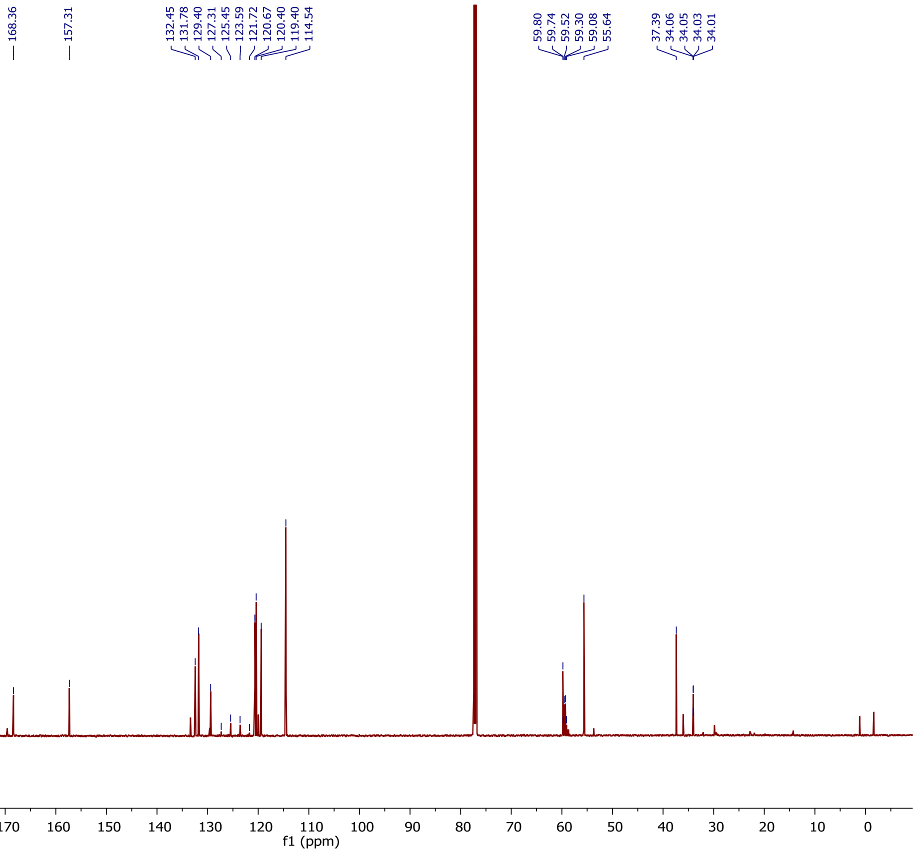

^{13}C NMR of 16

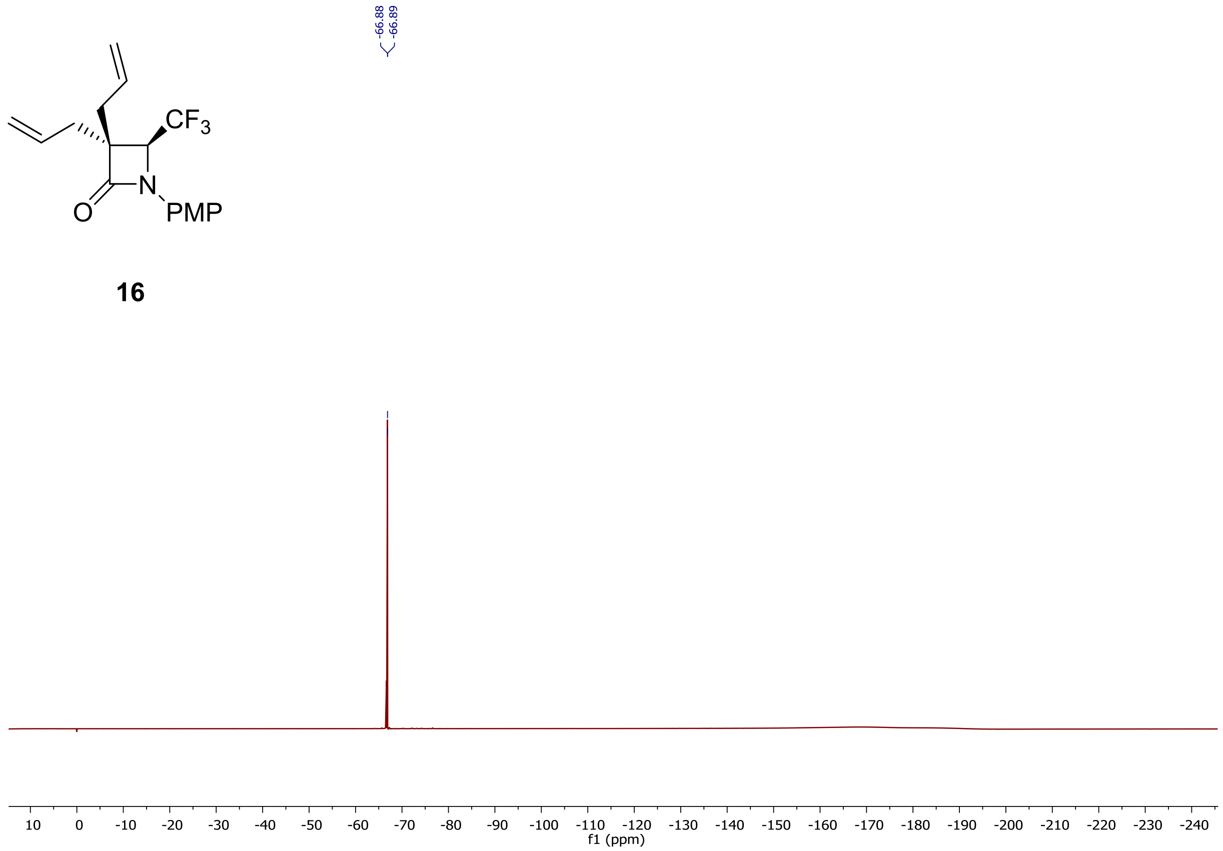

16

^{19}F NMR of 16

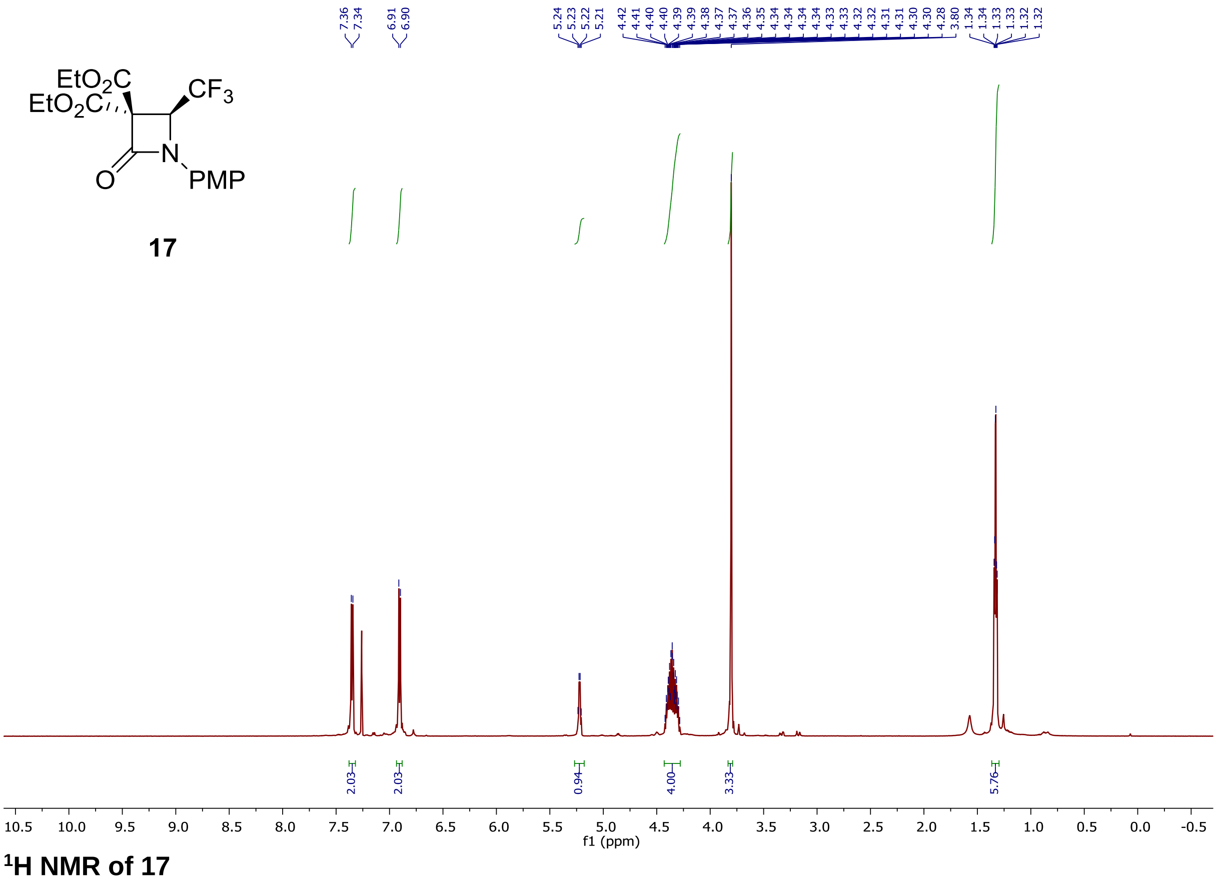

17

^{1}H NMR of 17

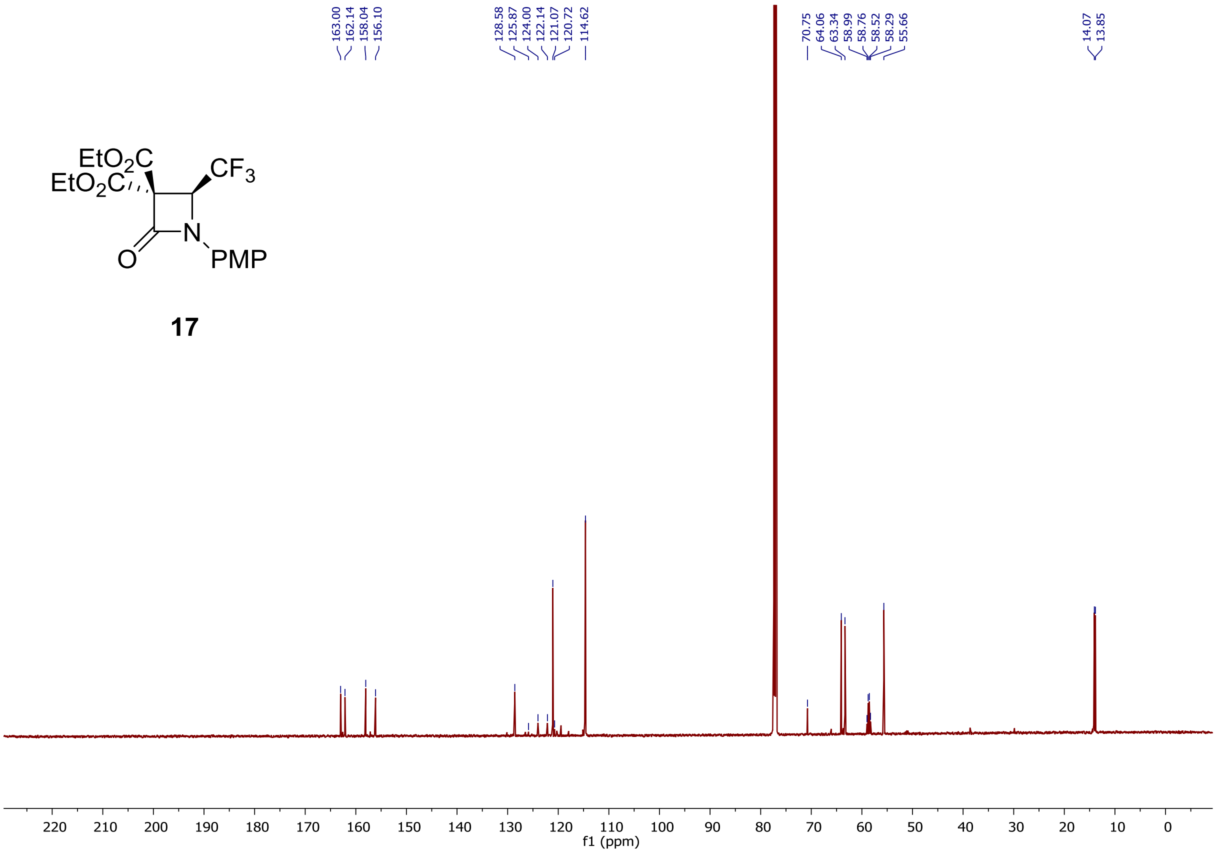

^{13}C NMR of 17

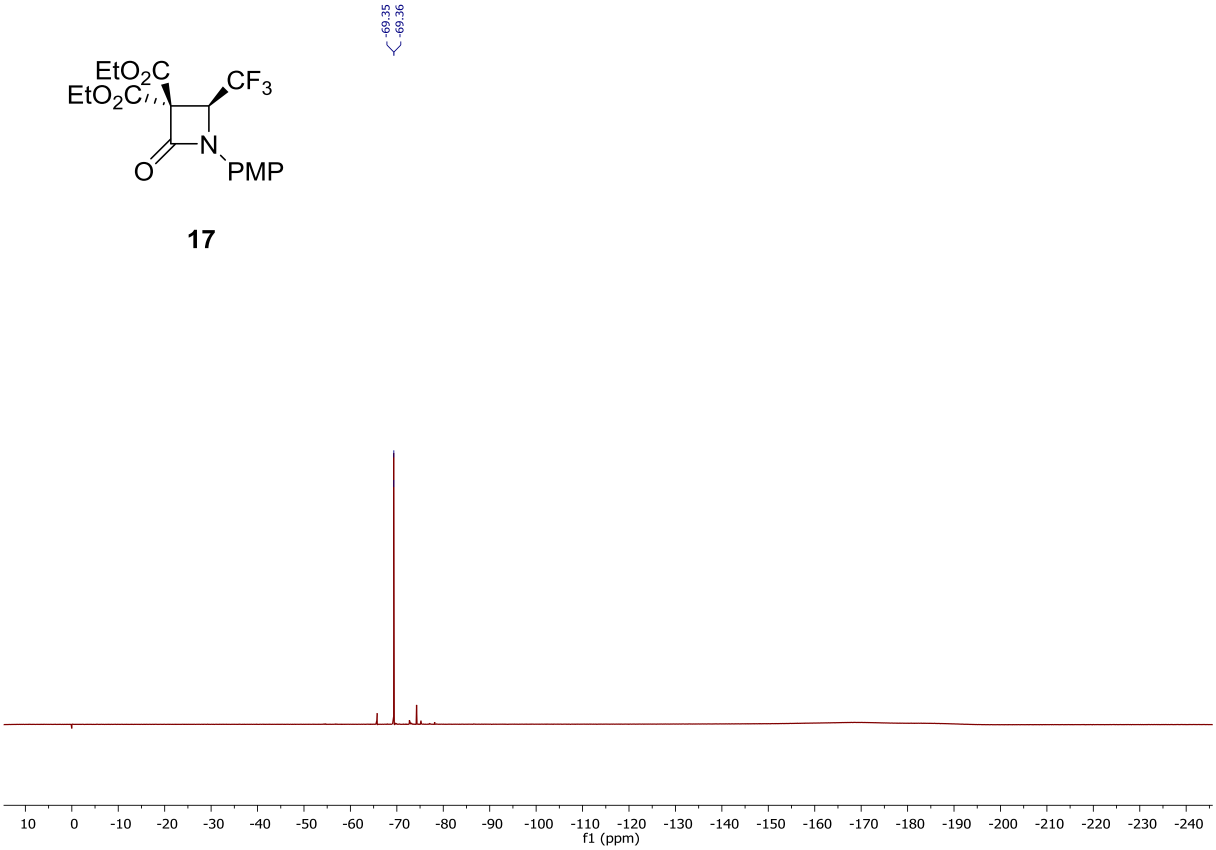

^{19}F NMR of 17

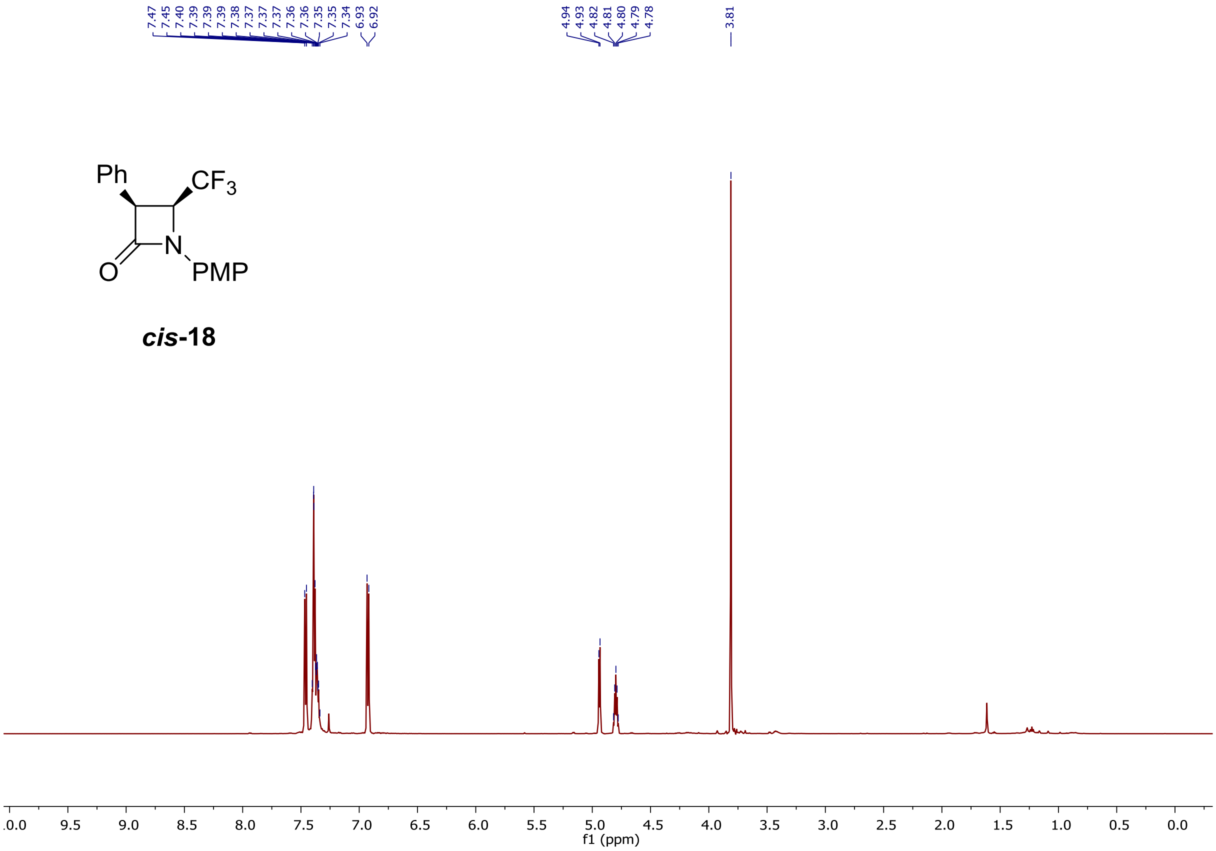

***cis*-18**

^{1}H NMR of *cis*-18

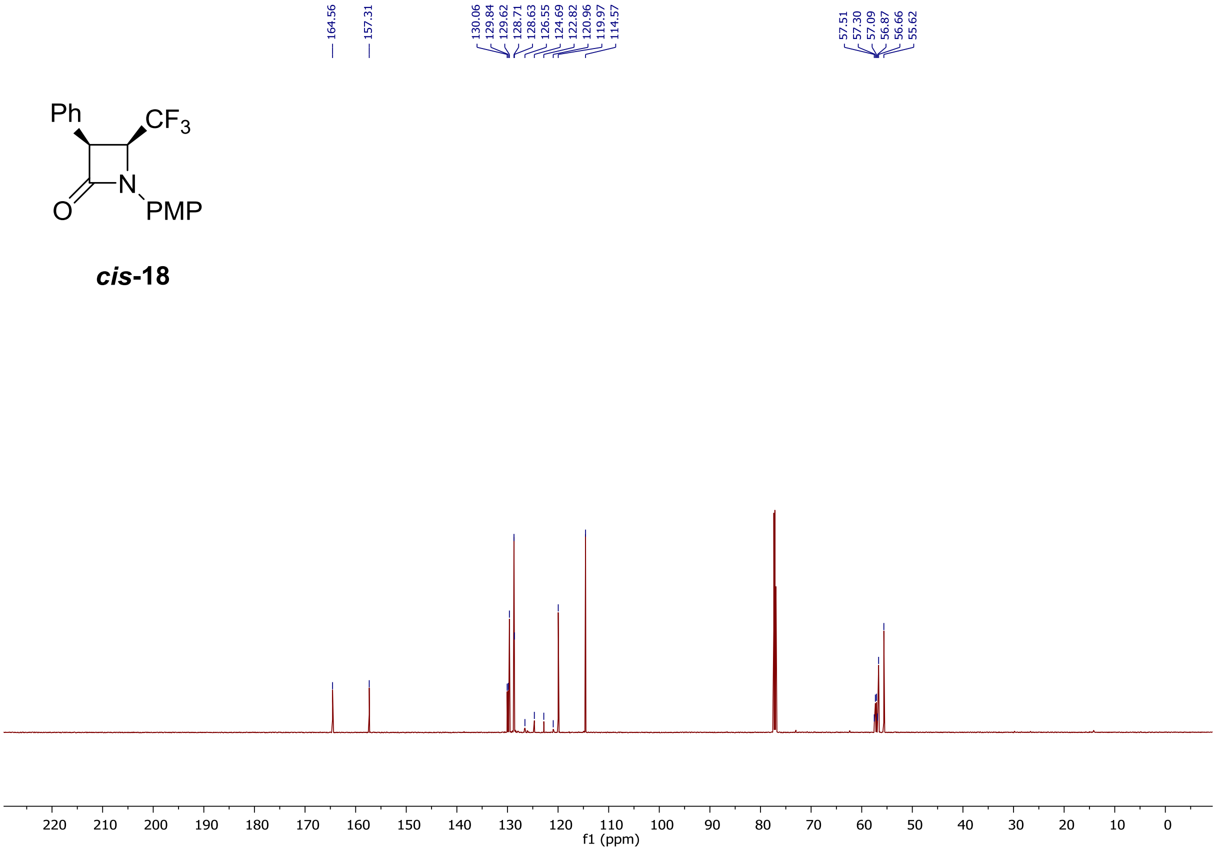

***cis*-18**

^{13}C NMR of *cis*-18

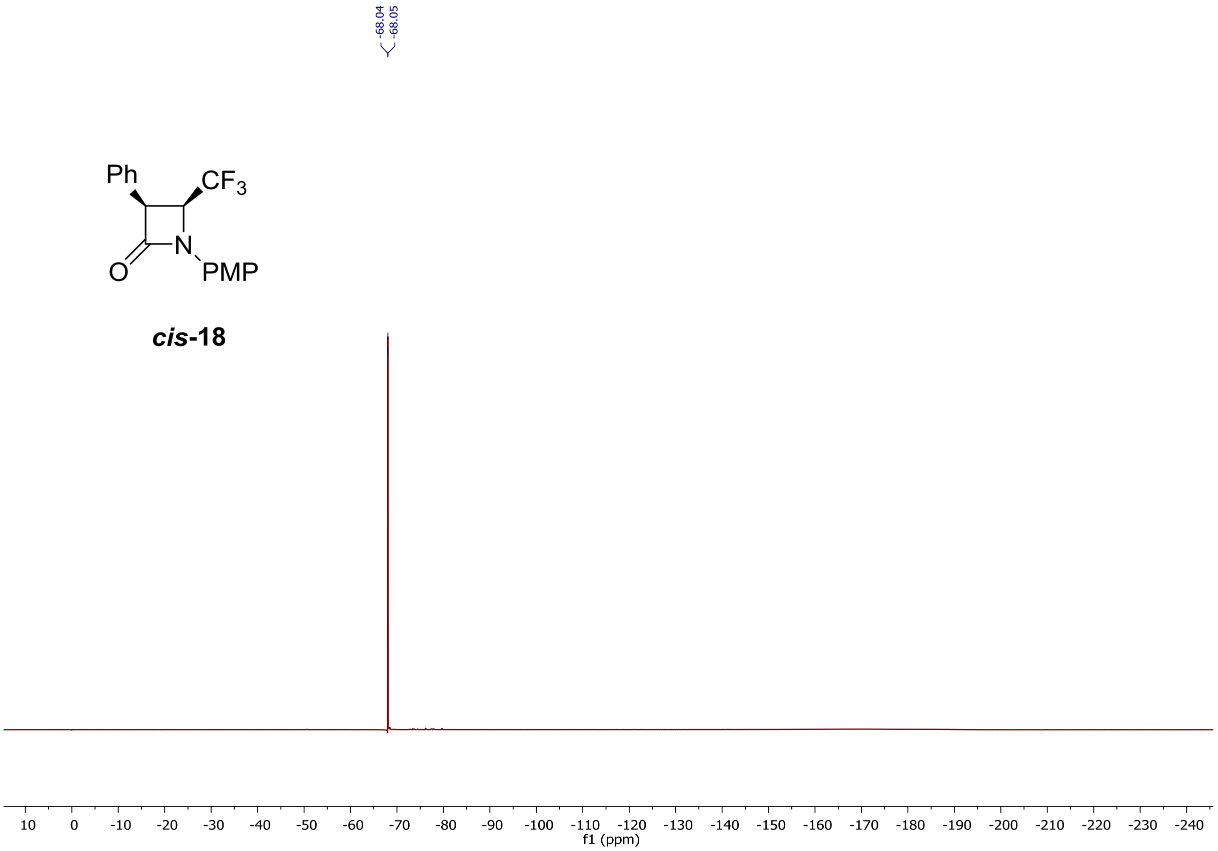

^{19}**F NMR of *cis*-18**

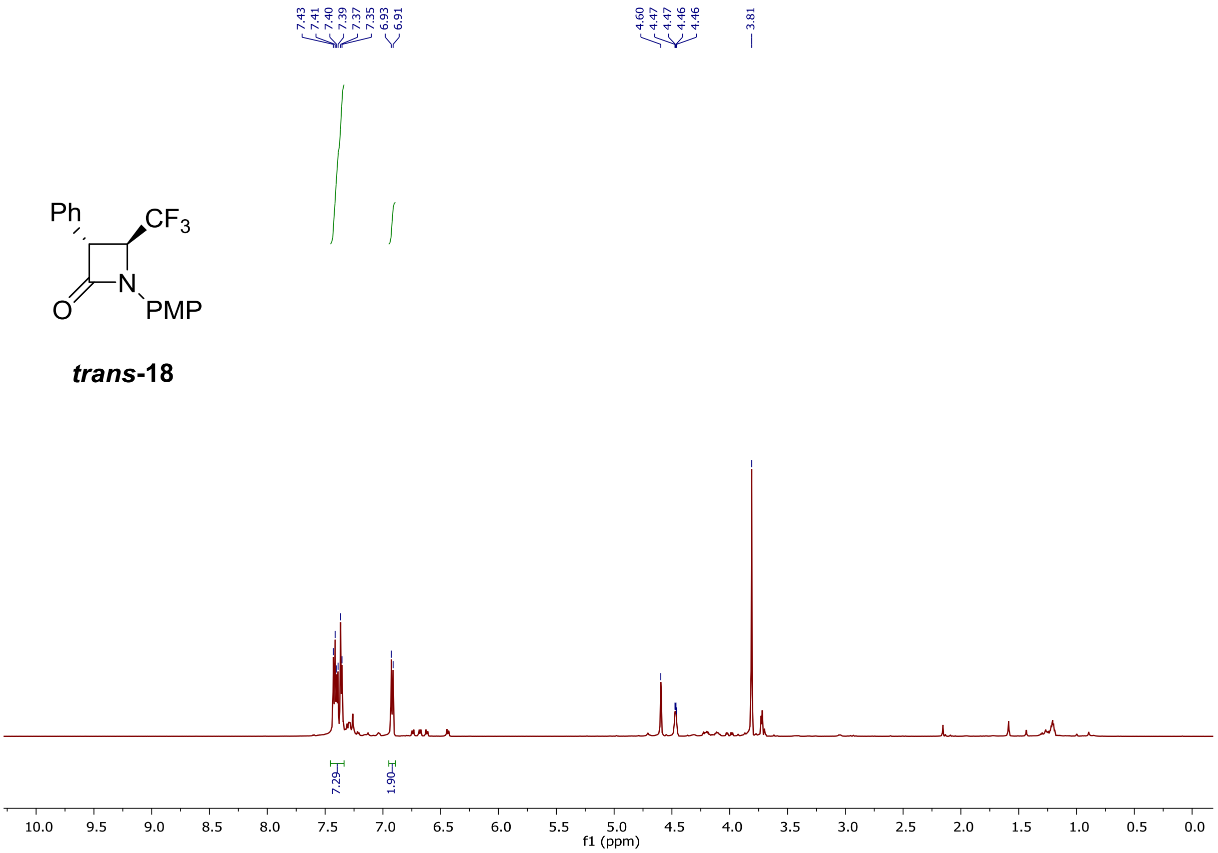

^{1}**H NMR of *trans*-18**

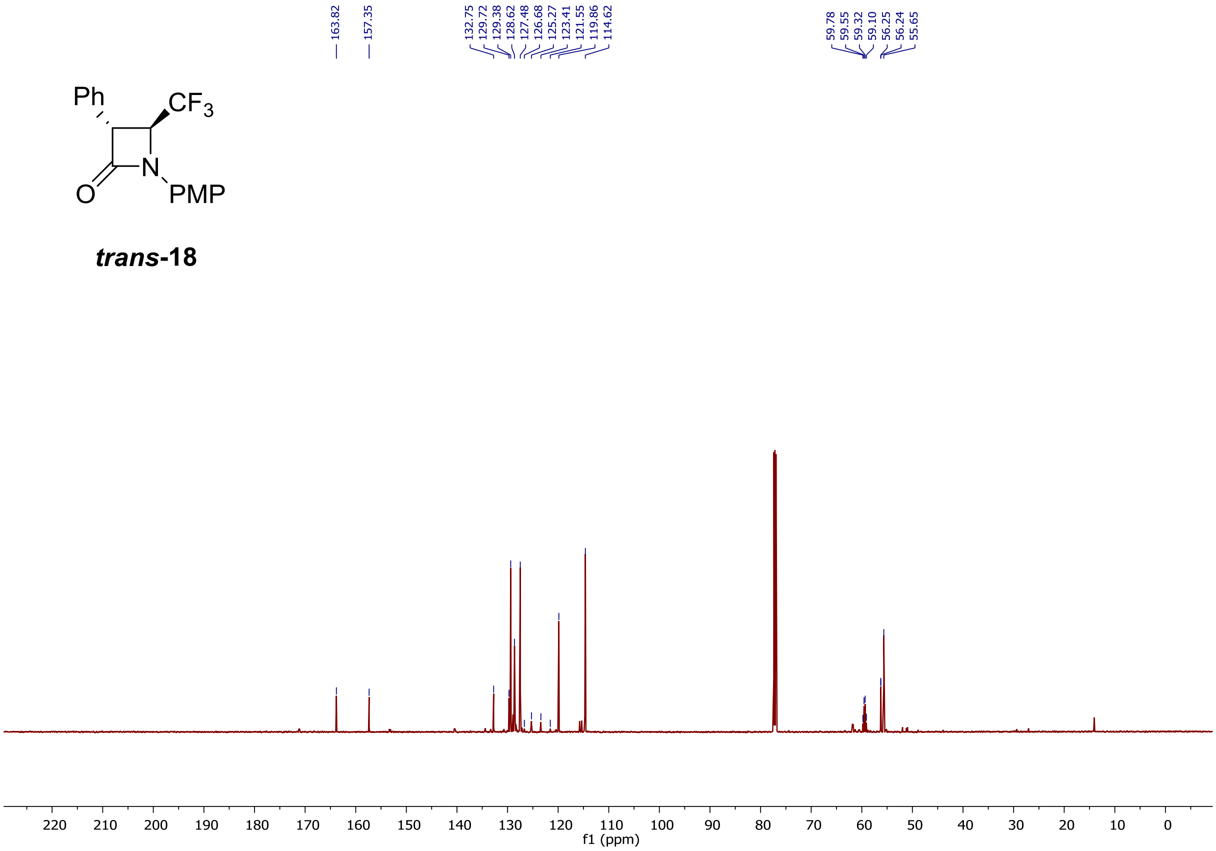

^{13}C NMR of *trans*-18

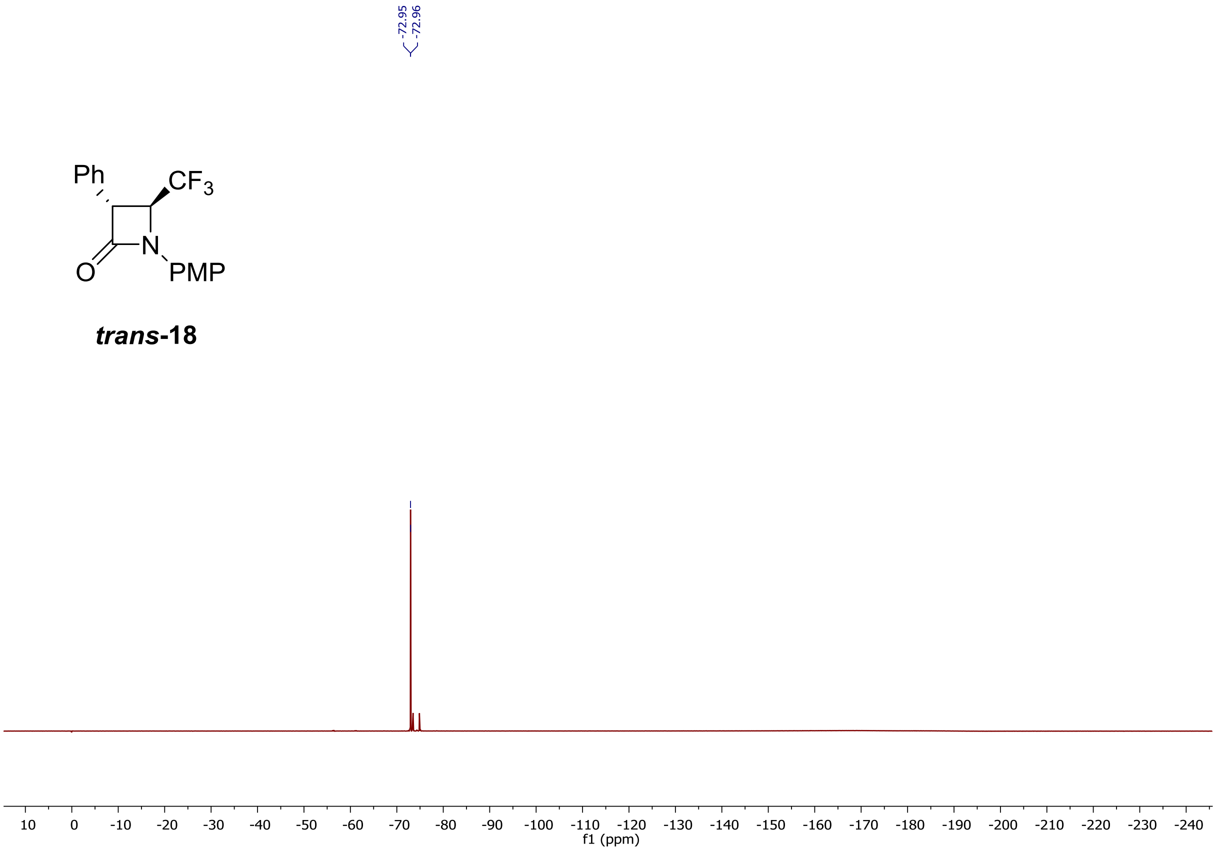

^{19}F NMR of *trans*-18

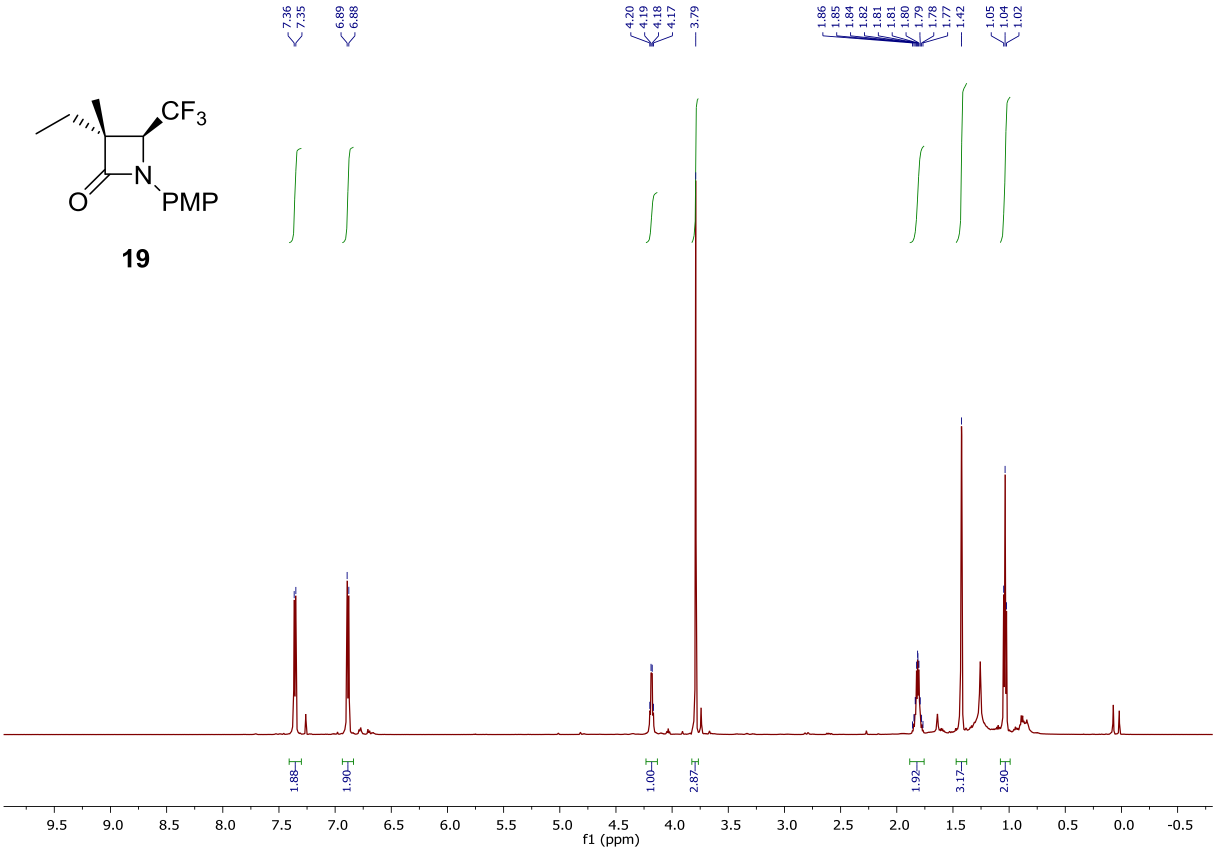

^{1}H NMR of 19

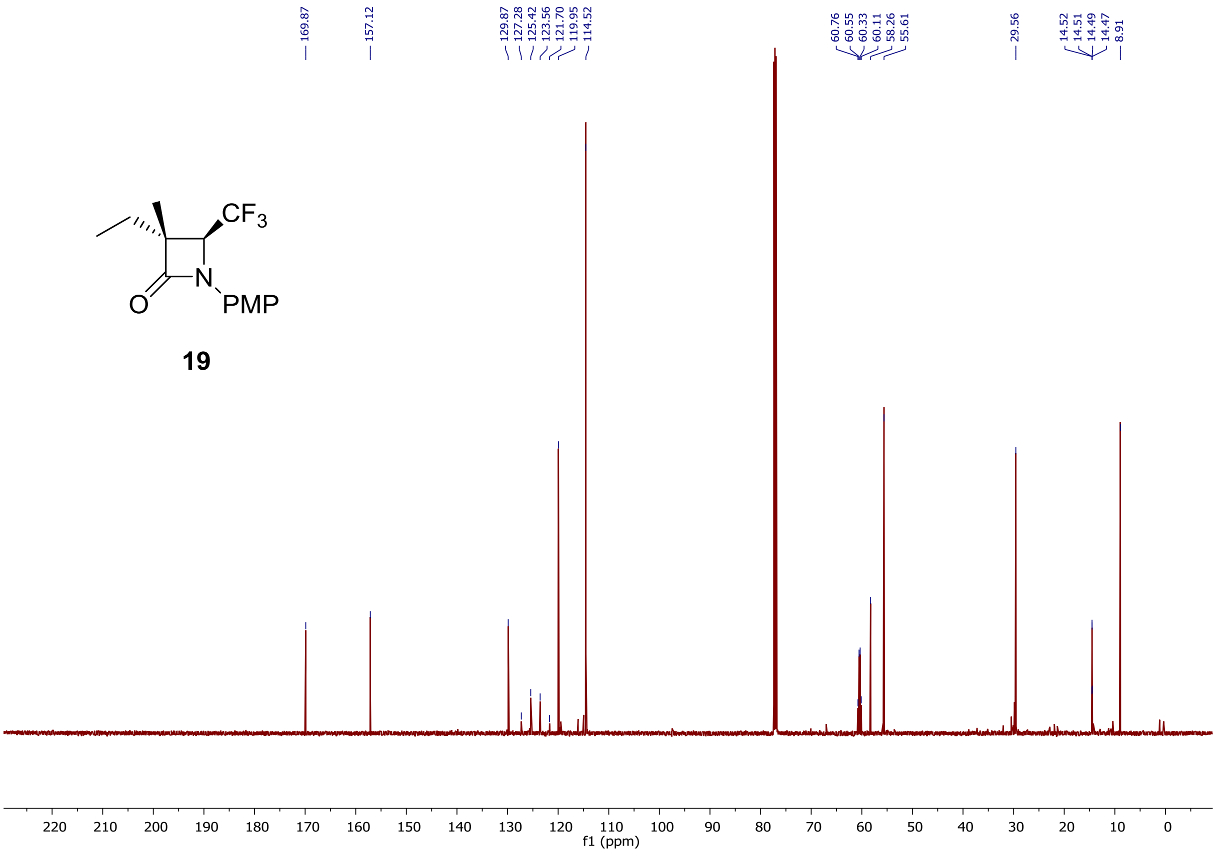

^{13}C NMR of 19

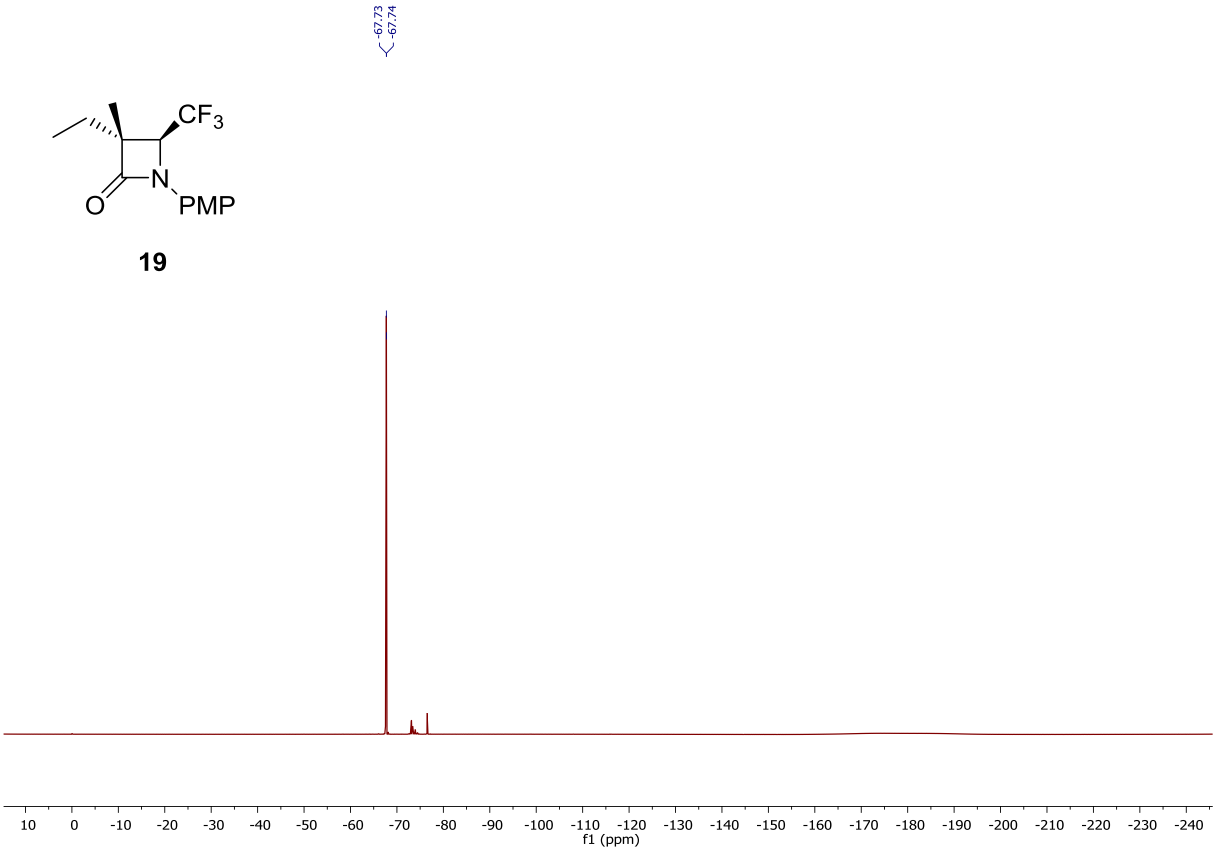

^{19}F NMR of 19

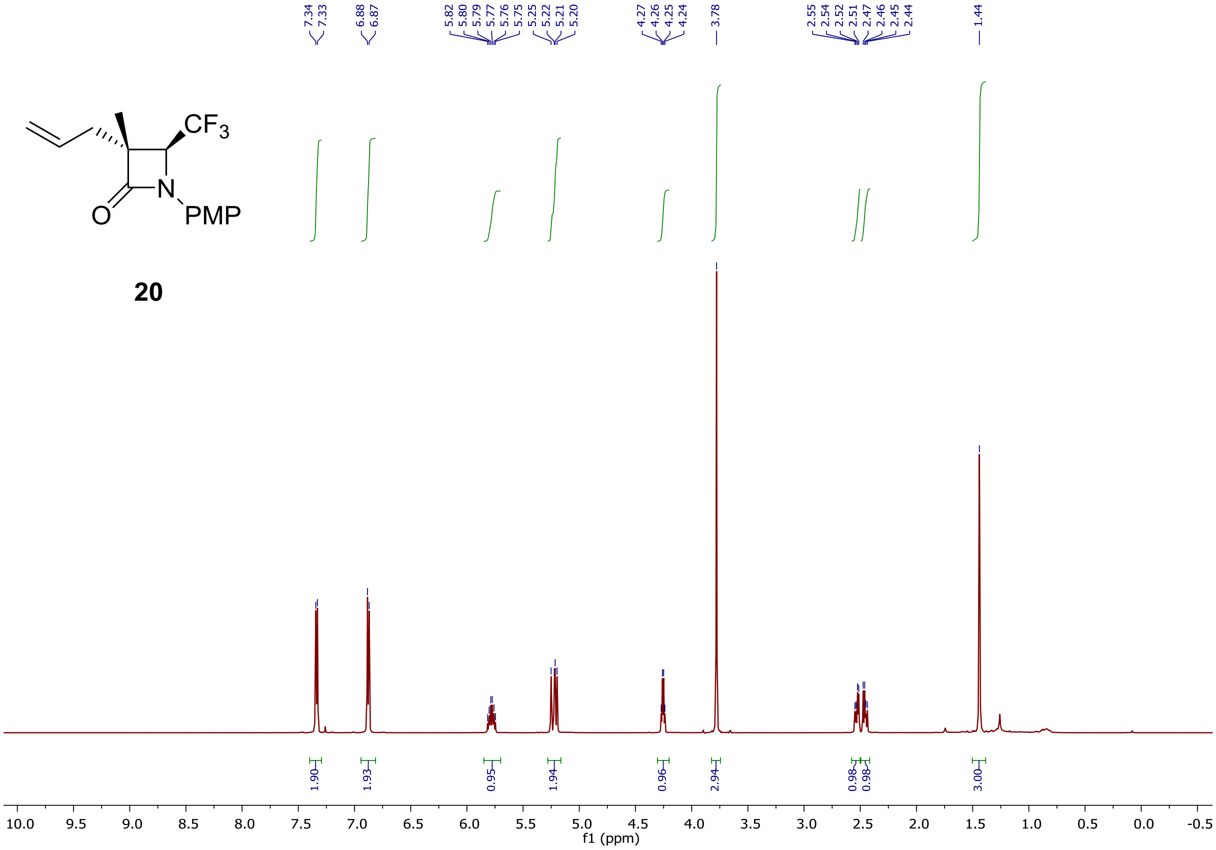

^{1}H NMR of 20

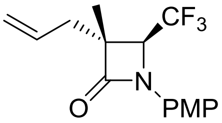

20

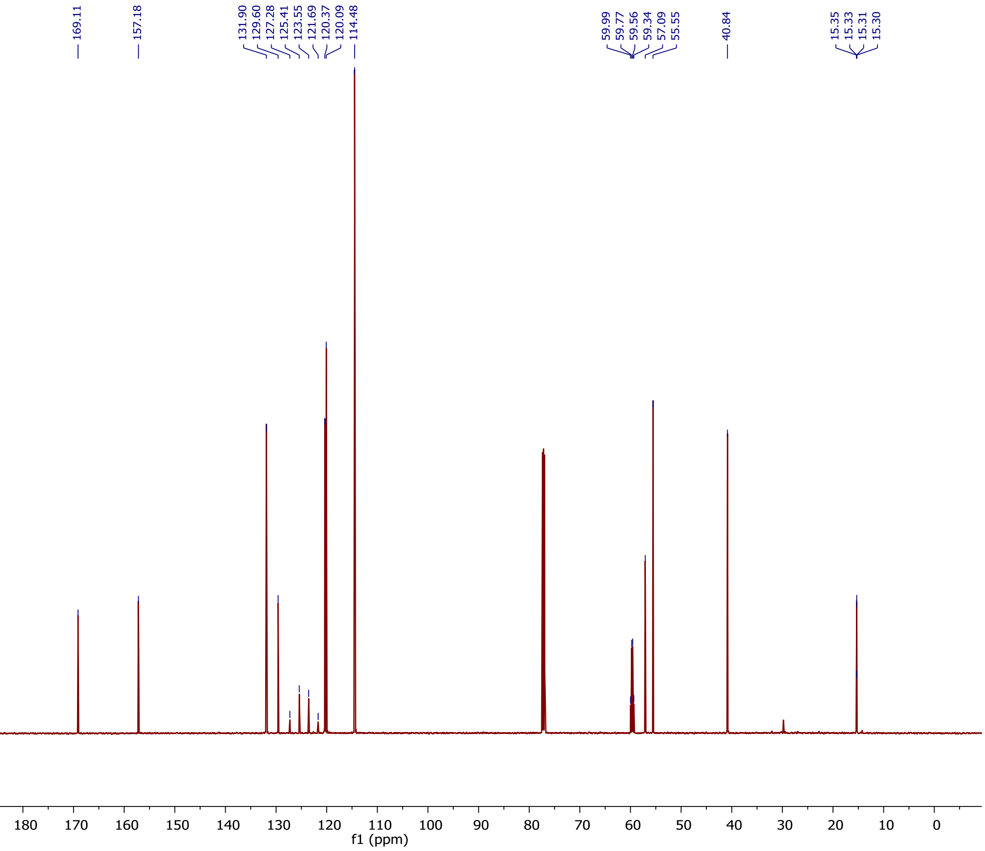

^{13}C NMR of 20

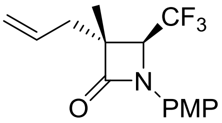

20

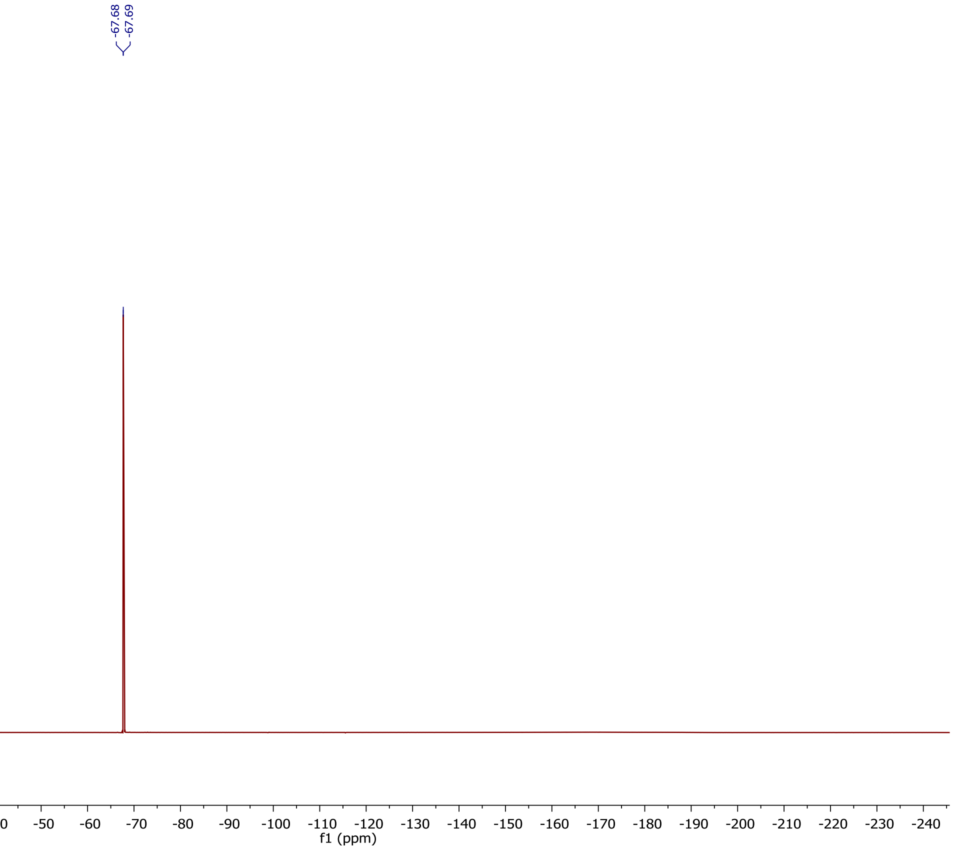

^{19}F NMR of 20

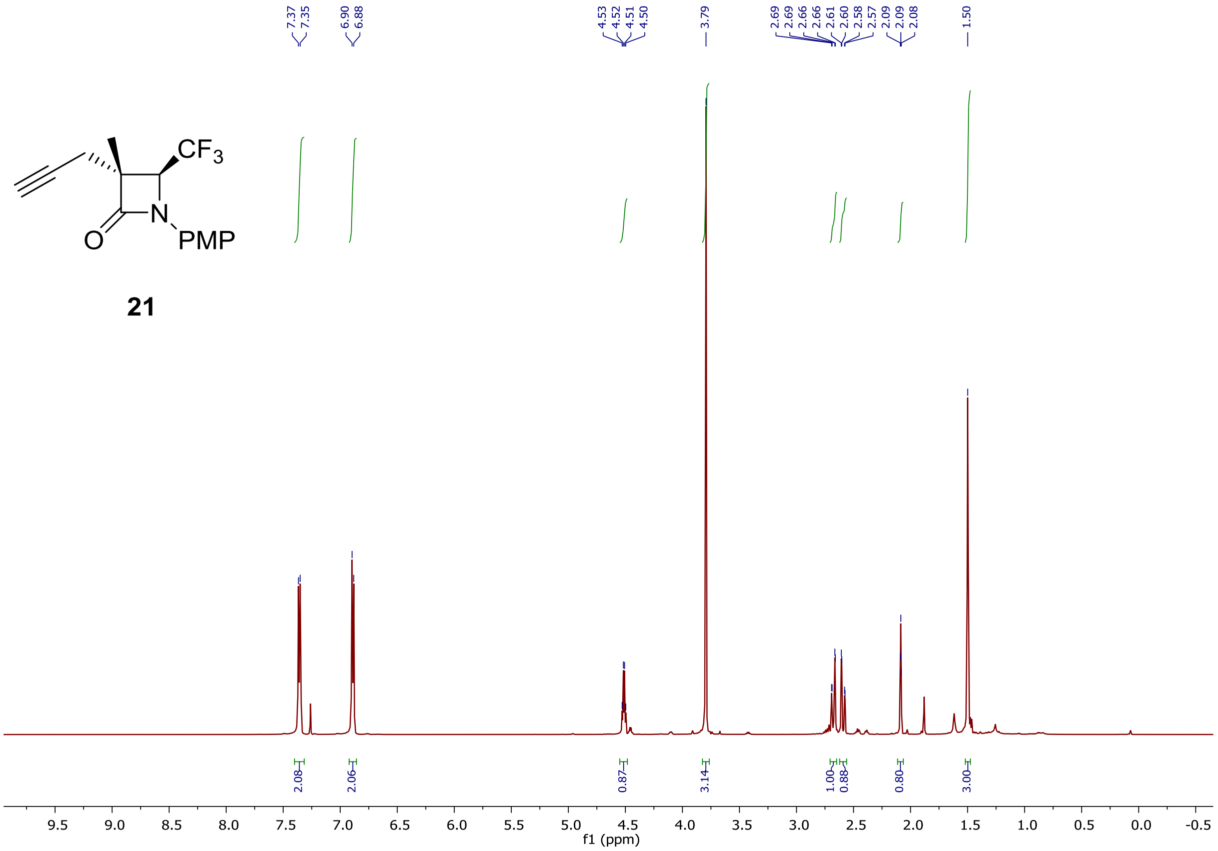

^{1}H NMR of 21

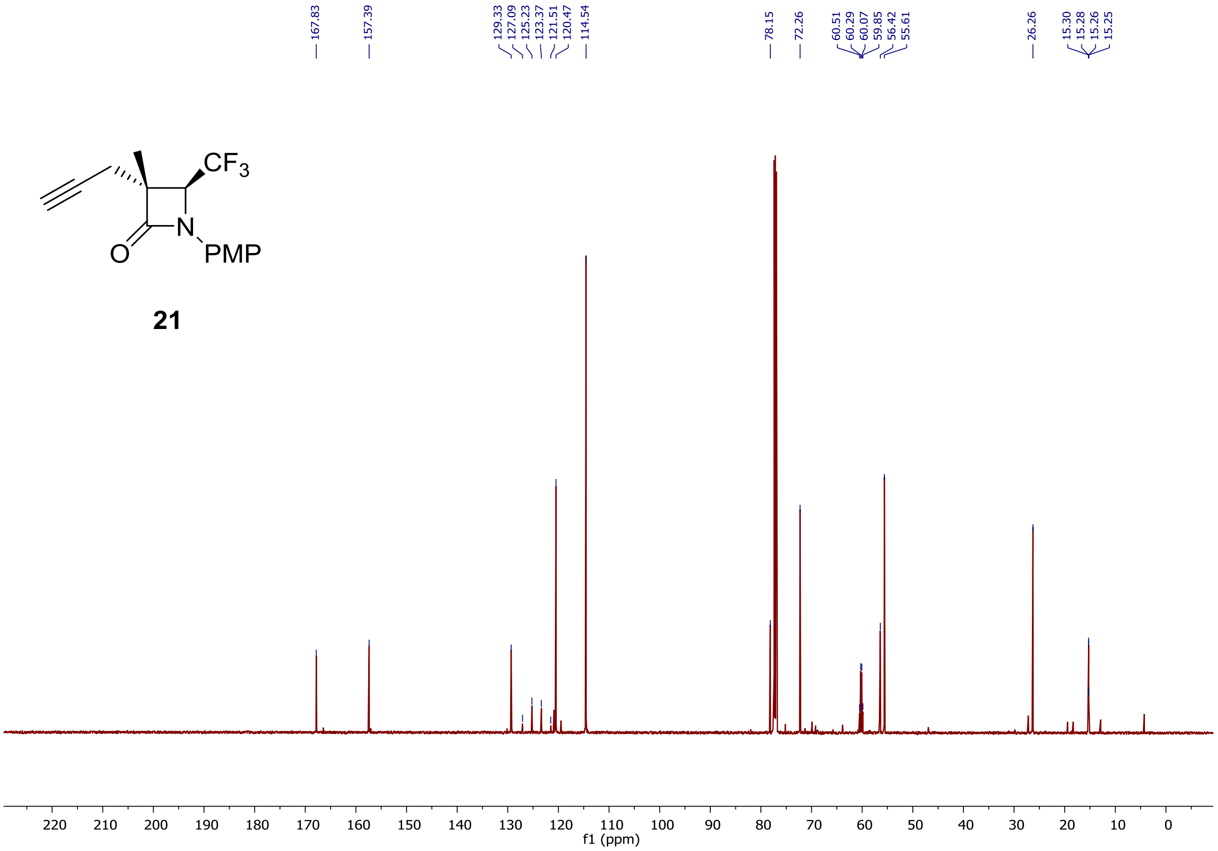

^{13}C NMR of 21

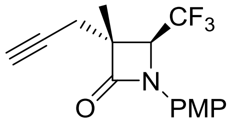

21

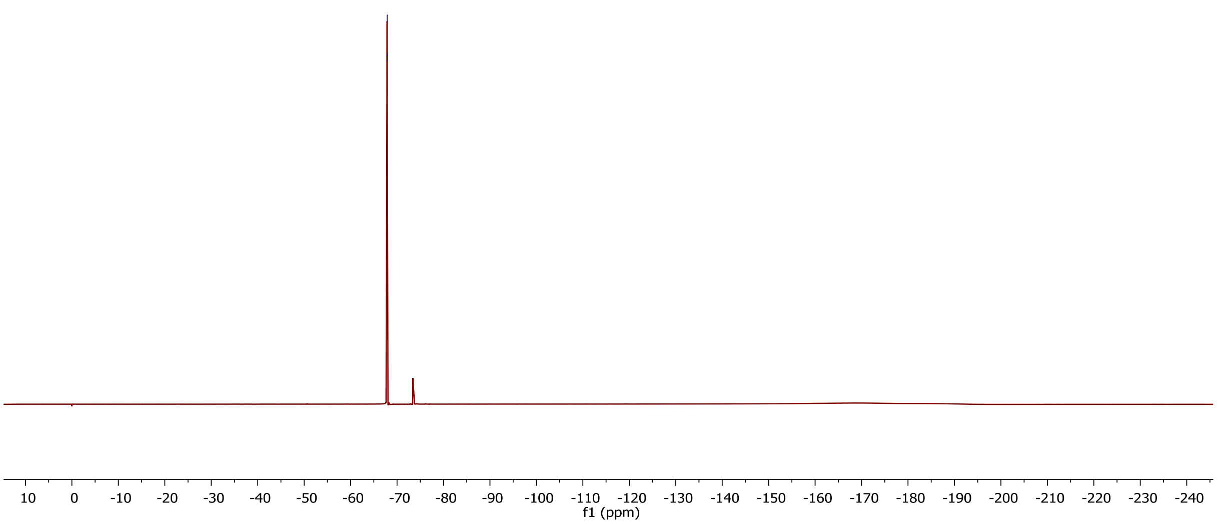

^{19}F NMR of 21

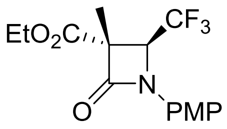

22

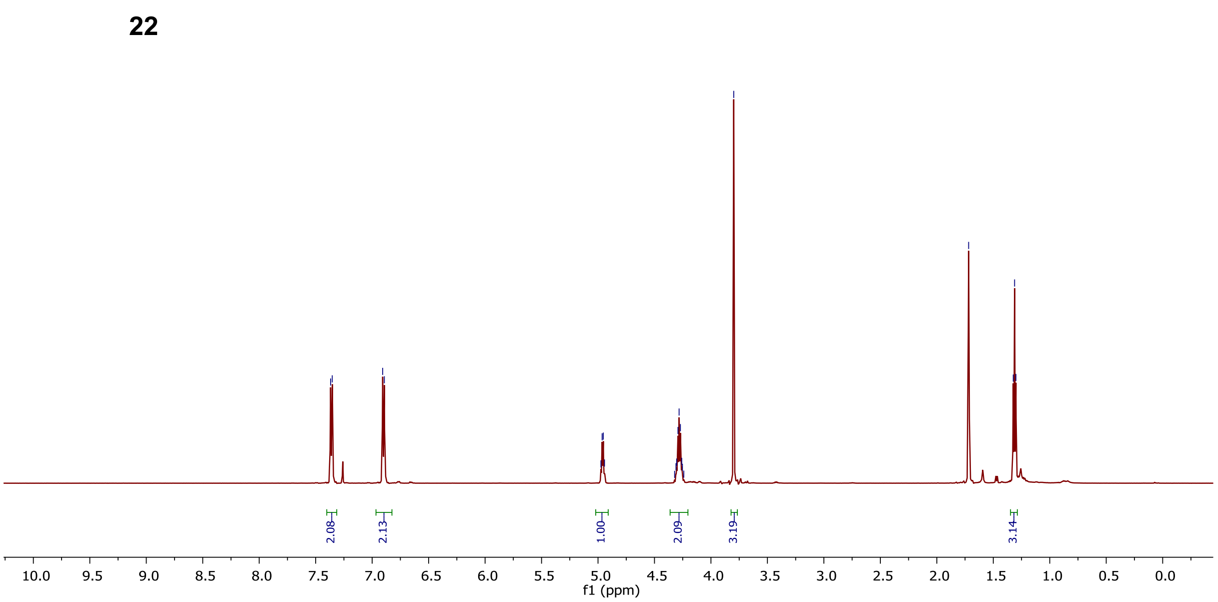

^{1}H NMR of 22

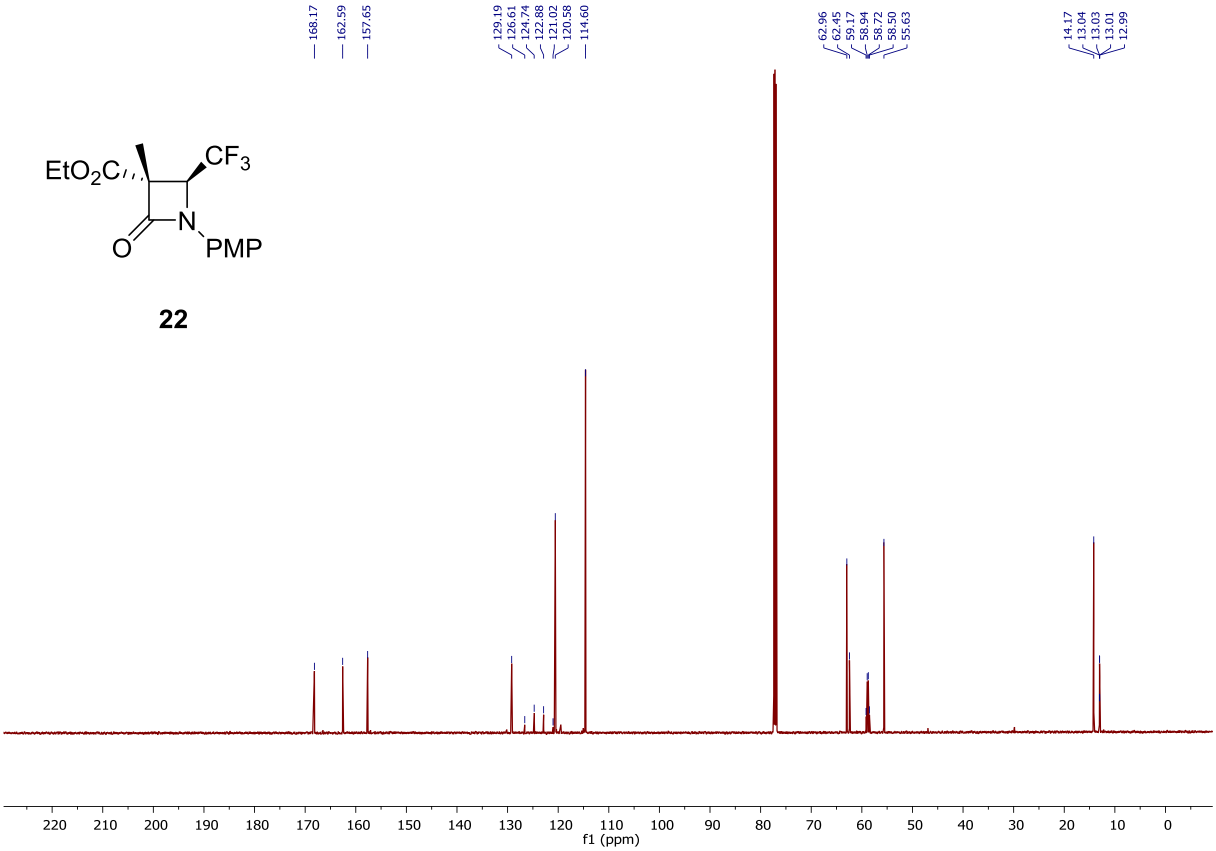

^{13}C NMR of 22

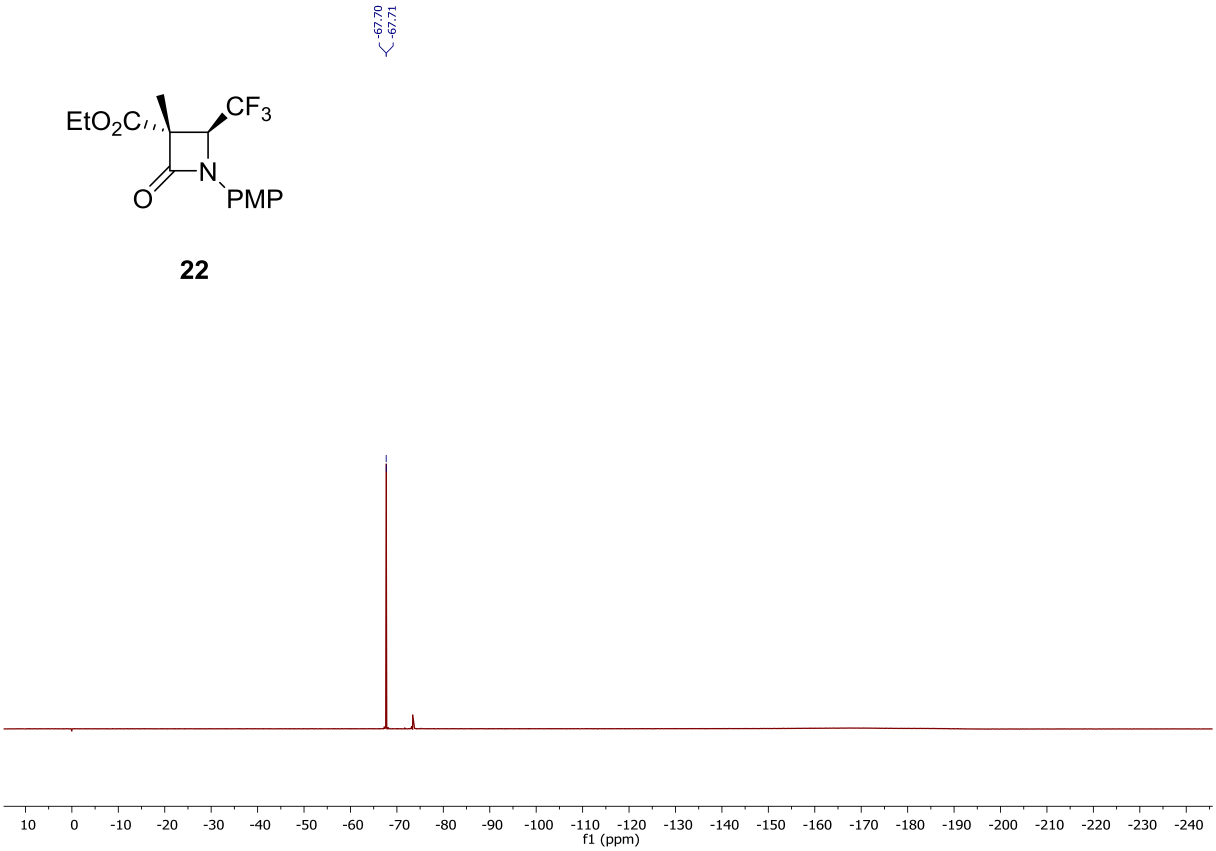

^{19}F NMR of 22

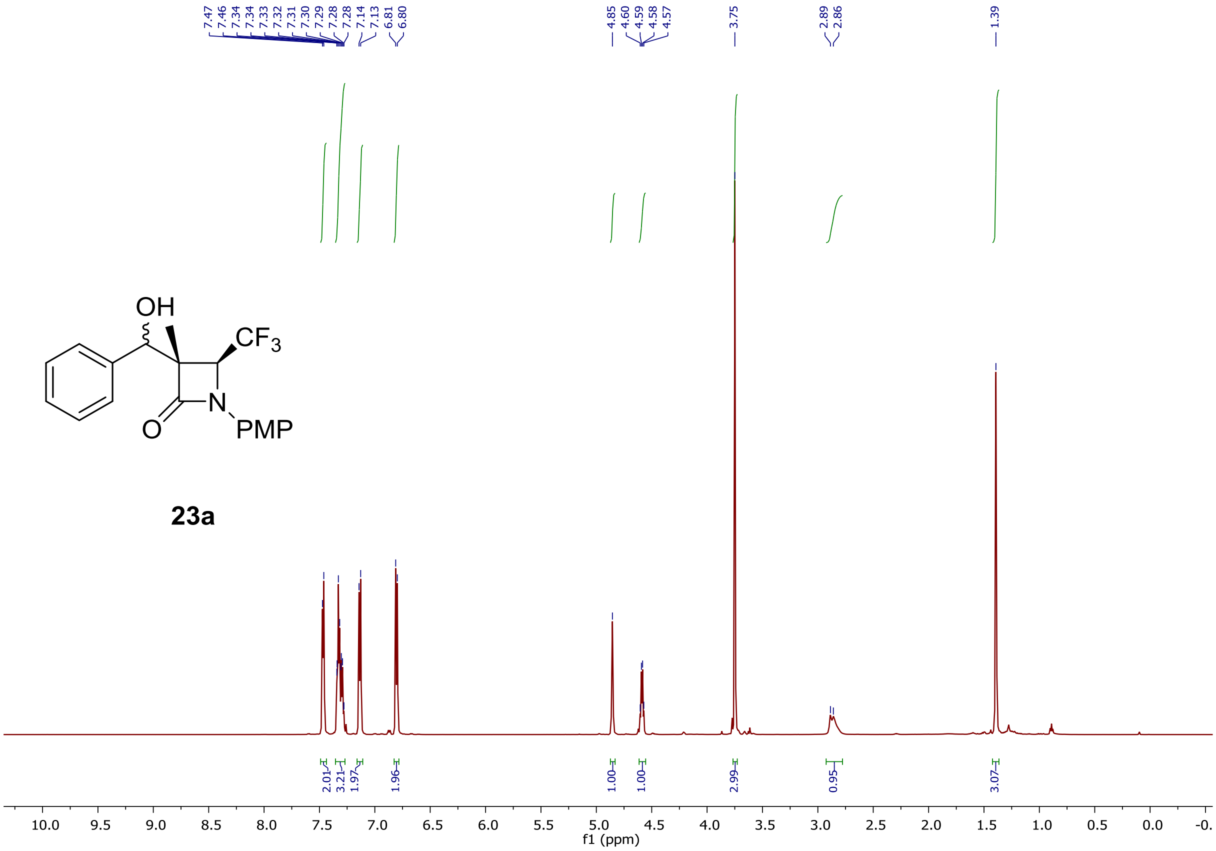

^{1}H NMR of 23a

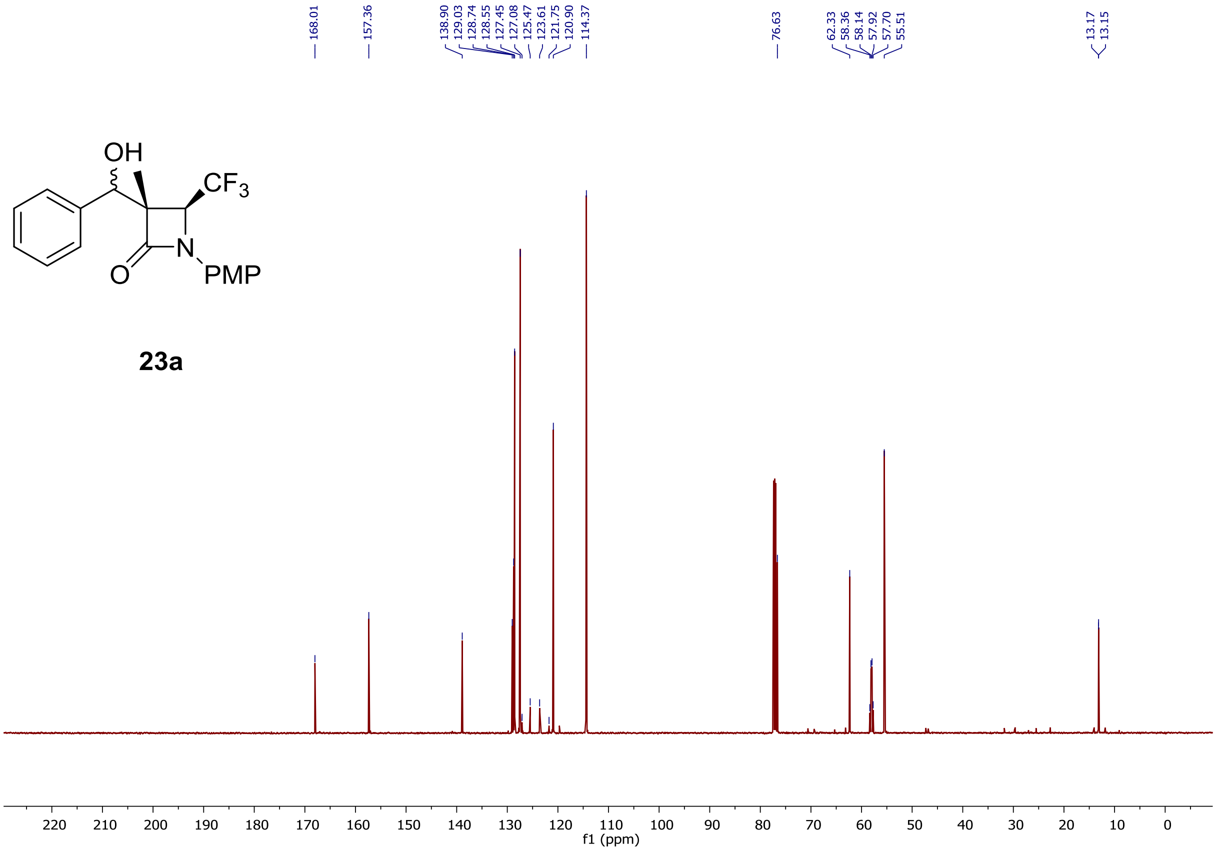

^{13}C NMR of 23a

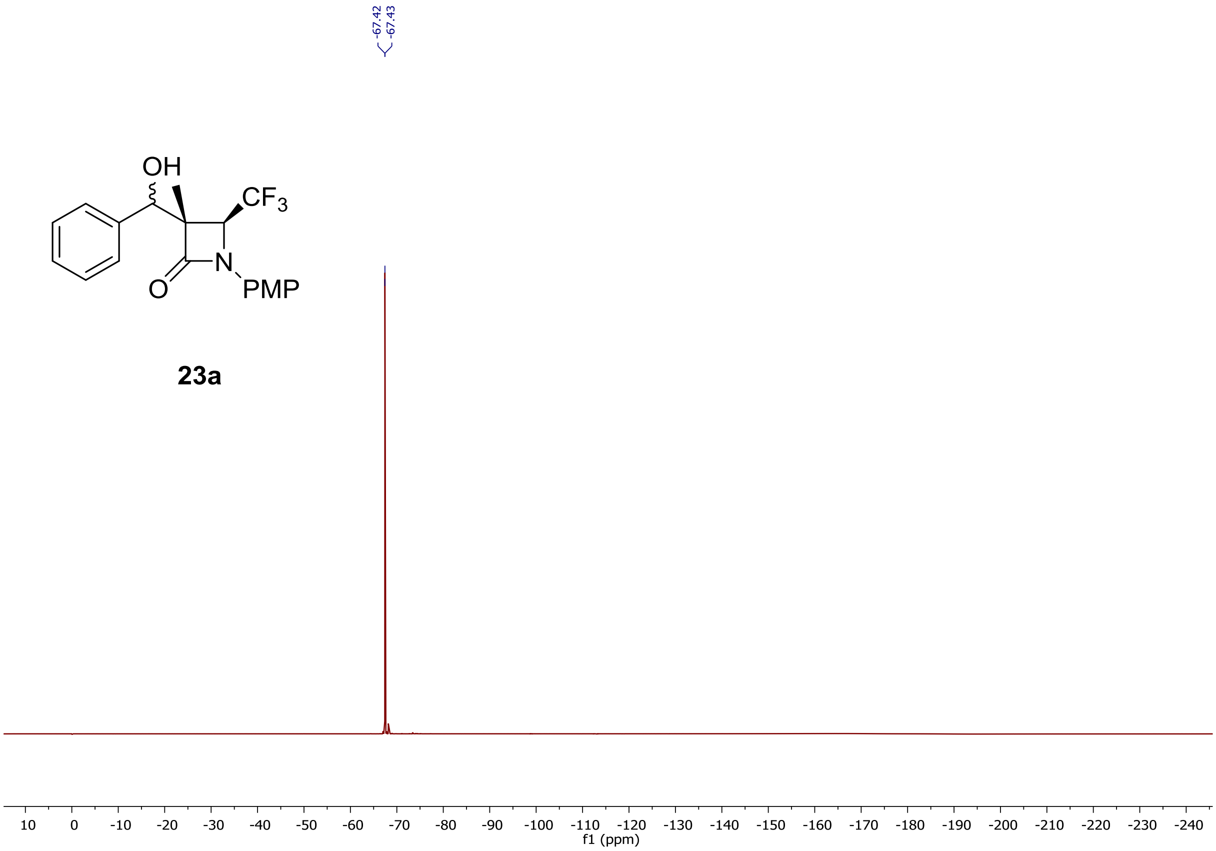

^{19}F NMR of 23a

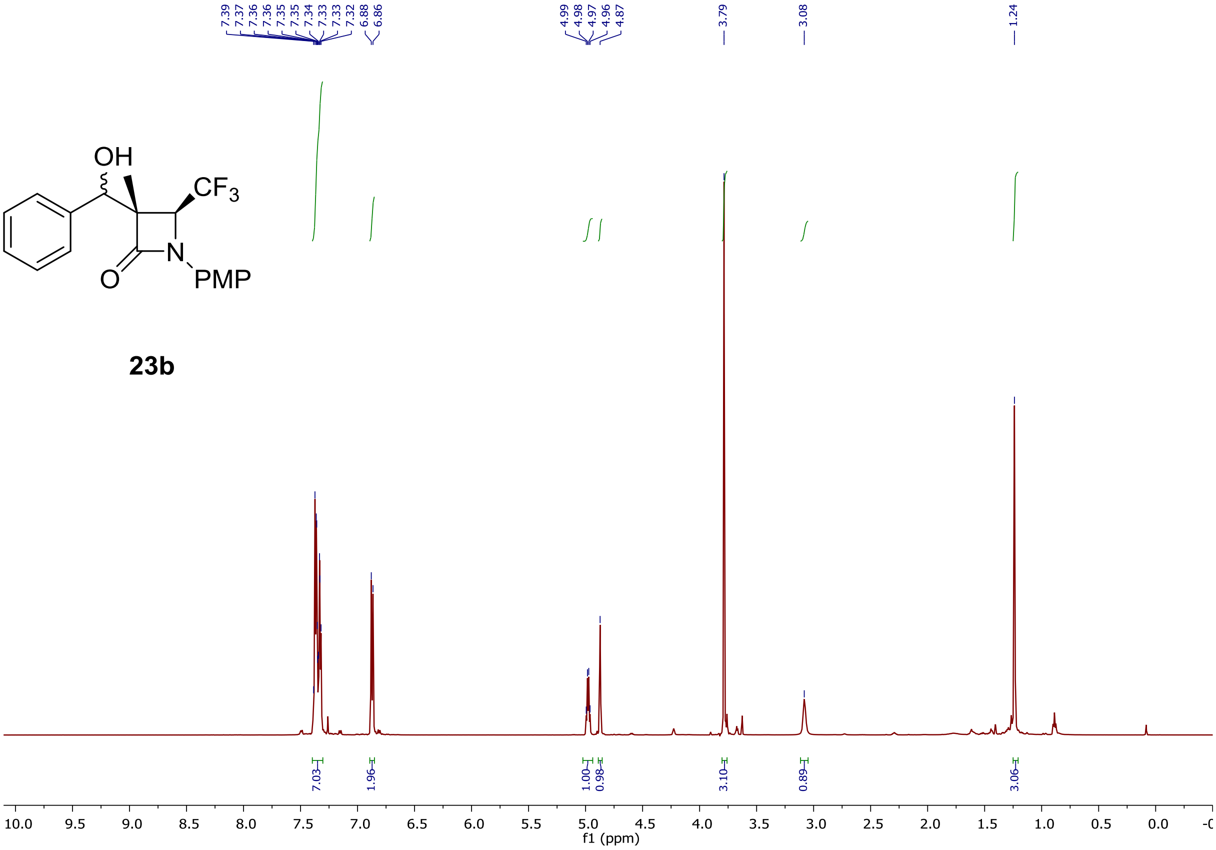

^{1}H NMR of 23b

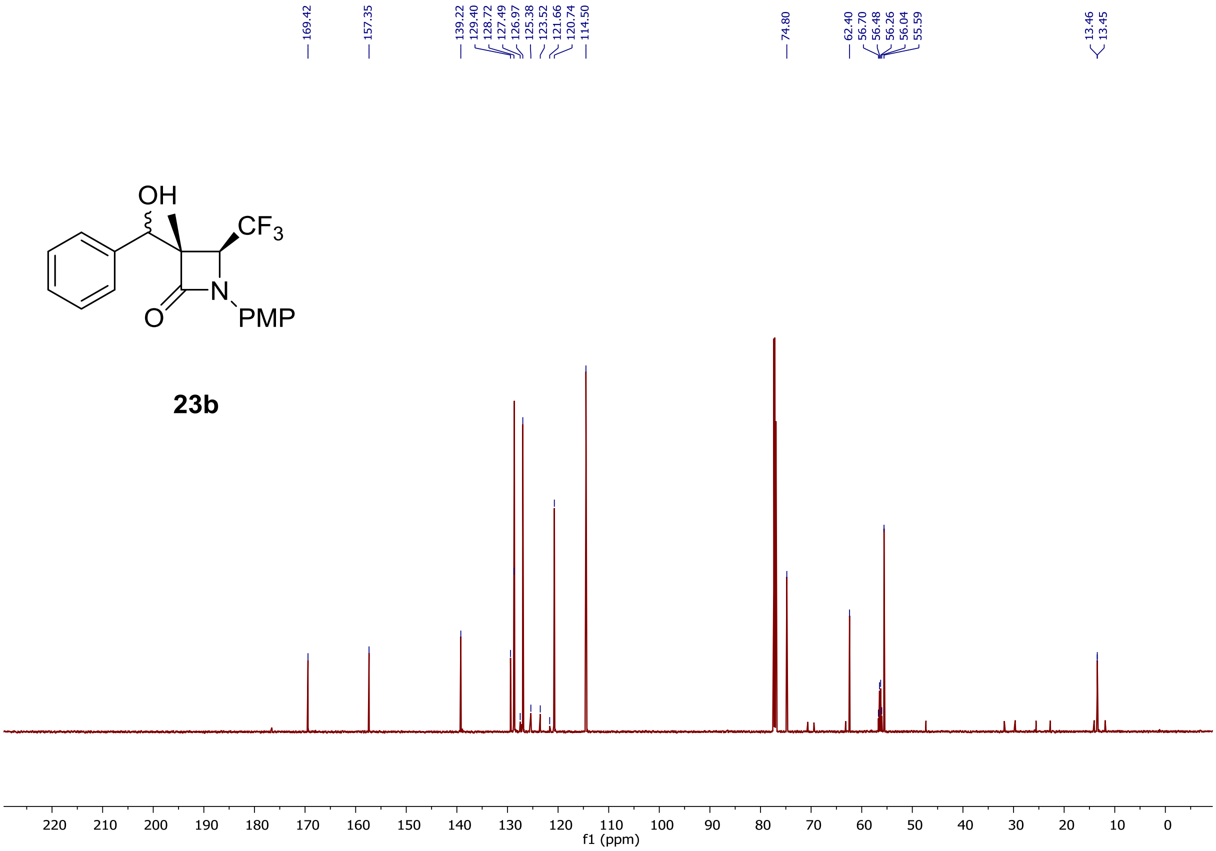

^{13}C NMR of 23b

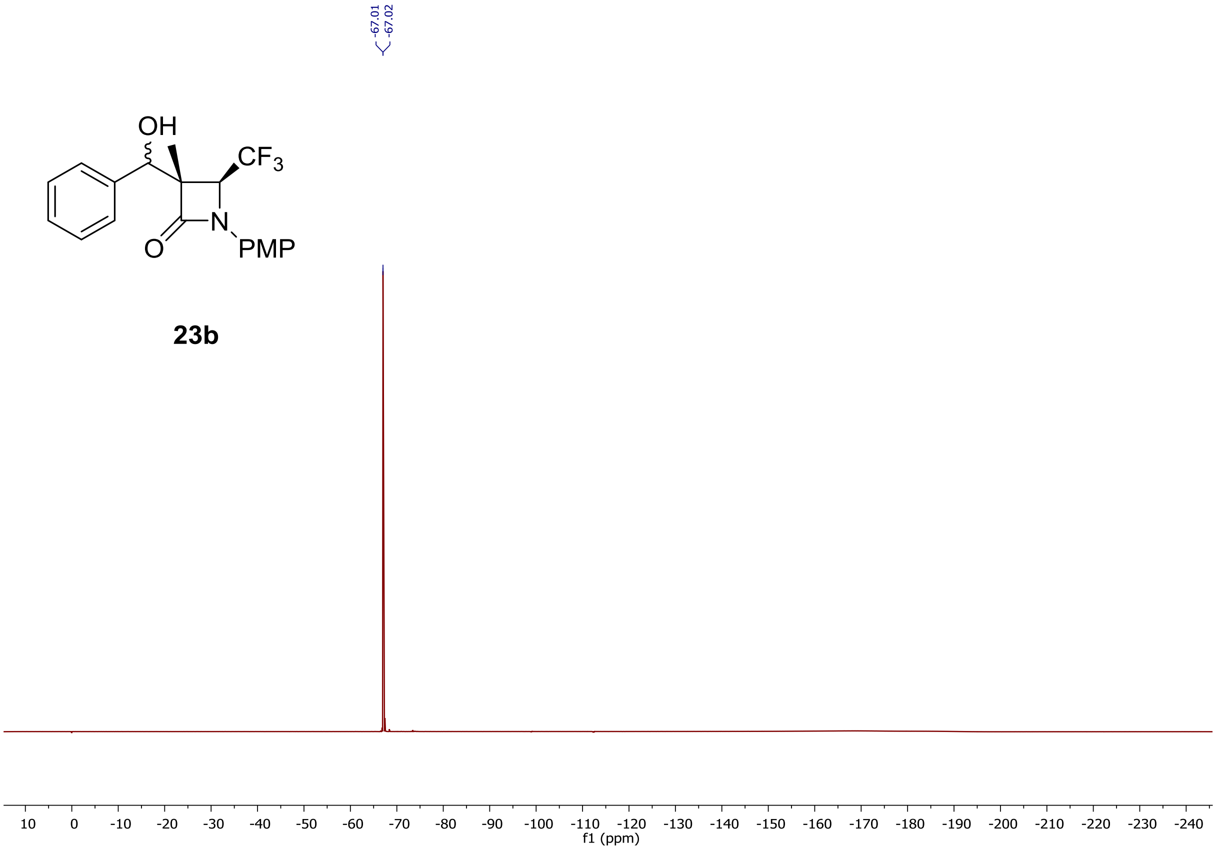

^{19}F NMR of 23b

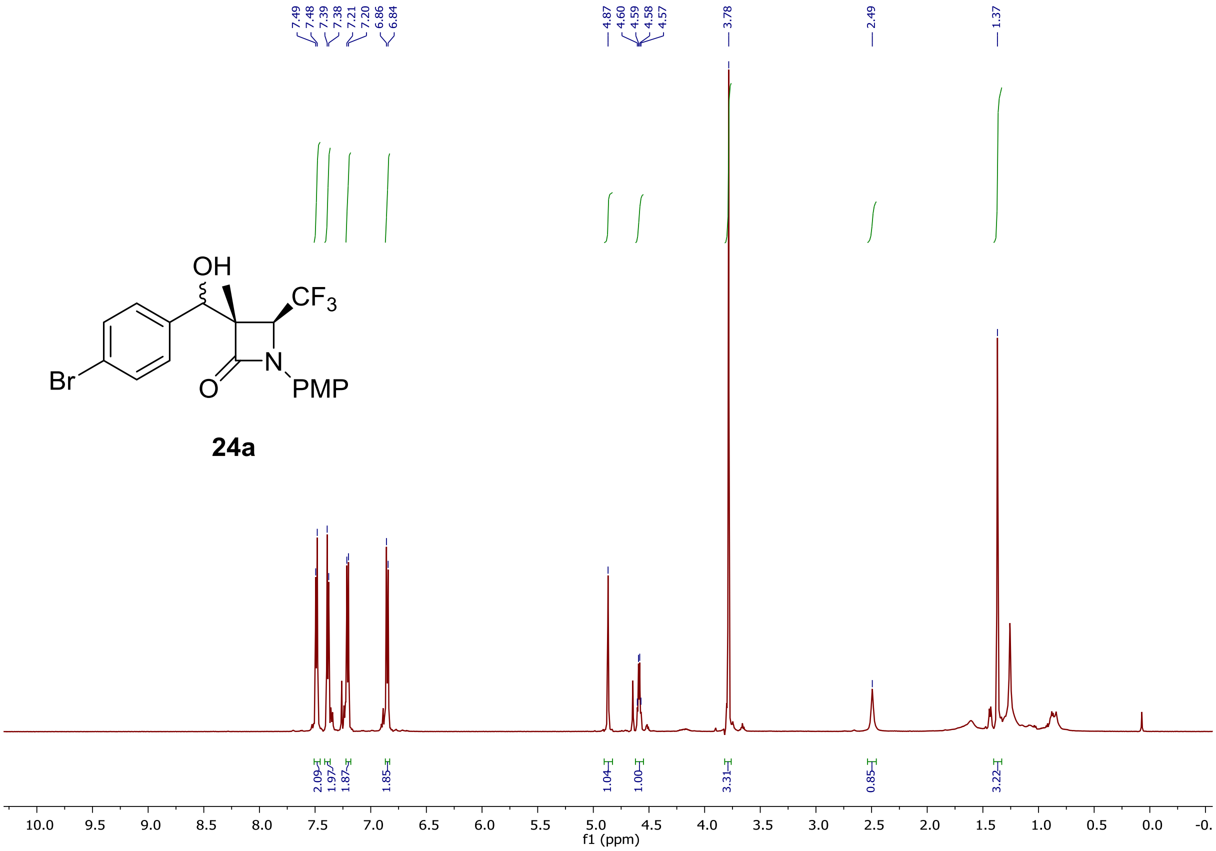

^{1}H NMR of 24a

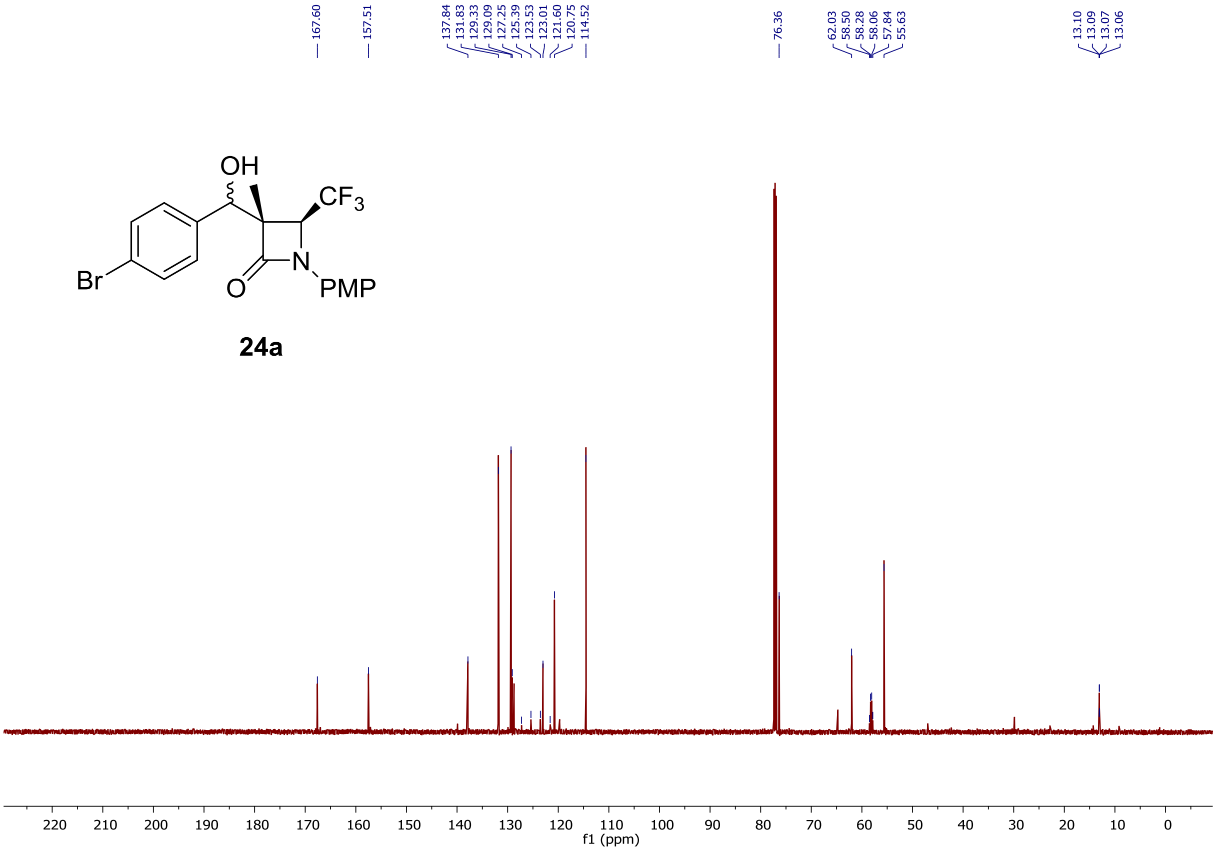

^{13}C NMR of 24a

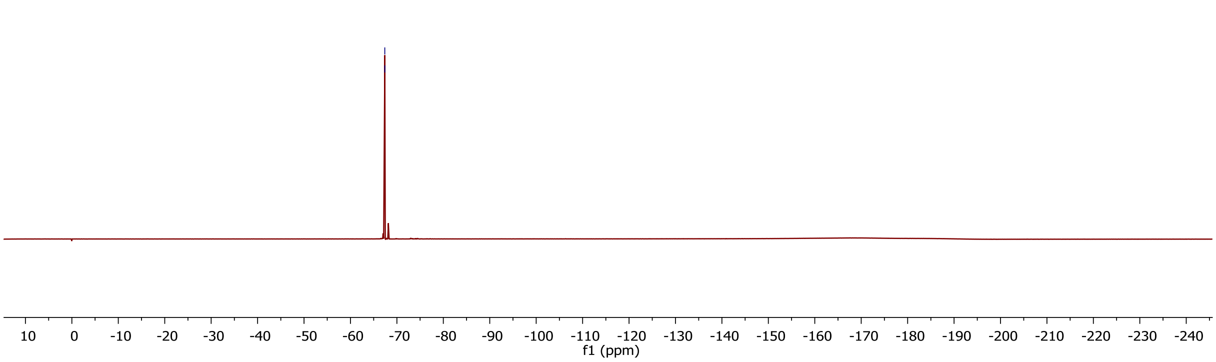

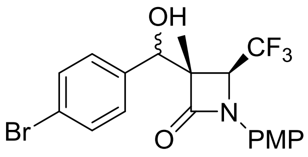

24a

^{19}F NMR of 24a

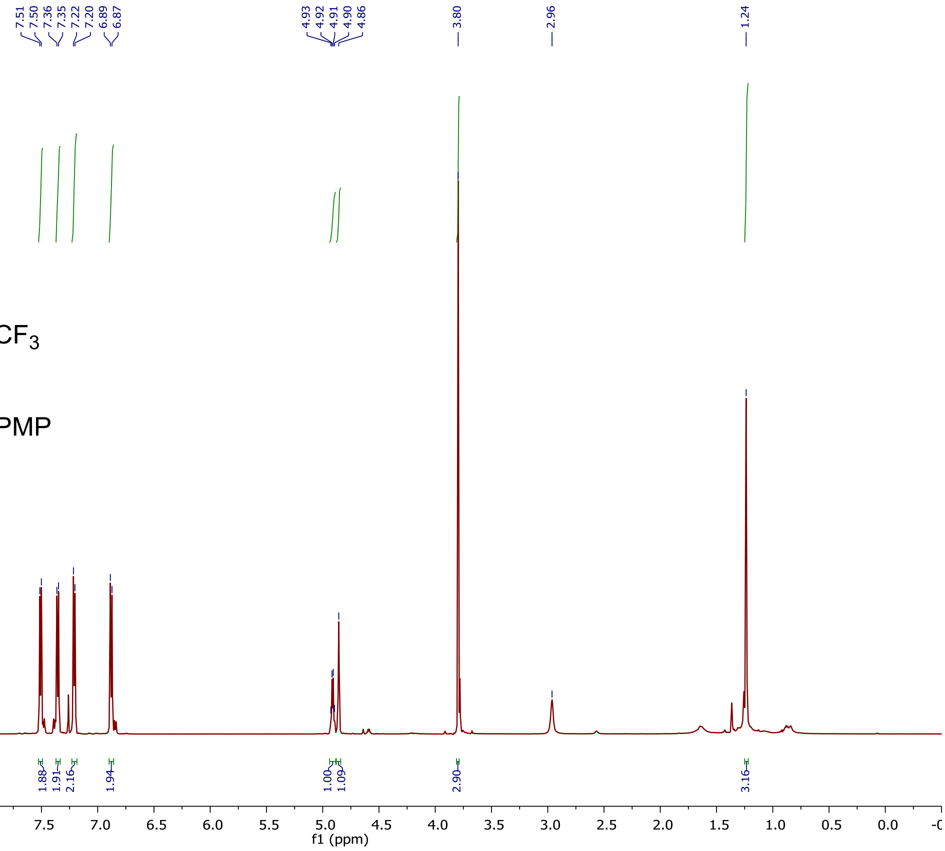

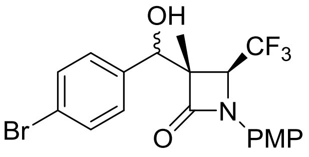

24b

^{1}H NMR of 24b

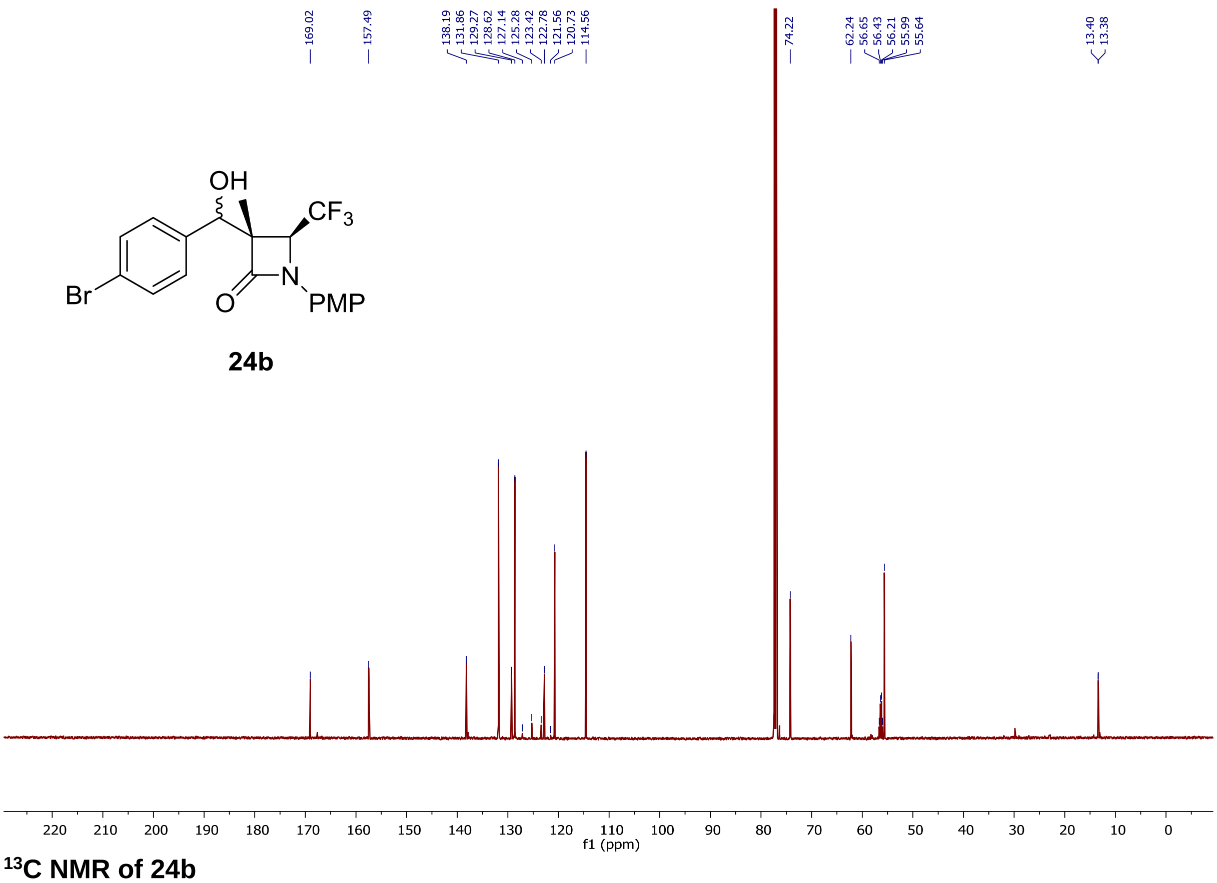

^{13}C NMR of 24b

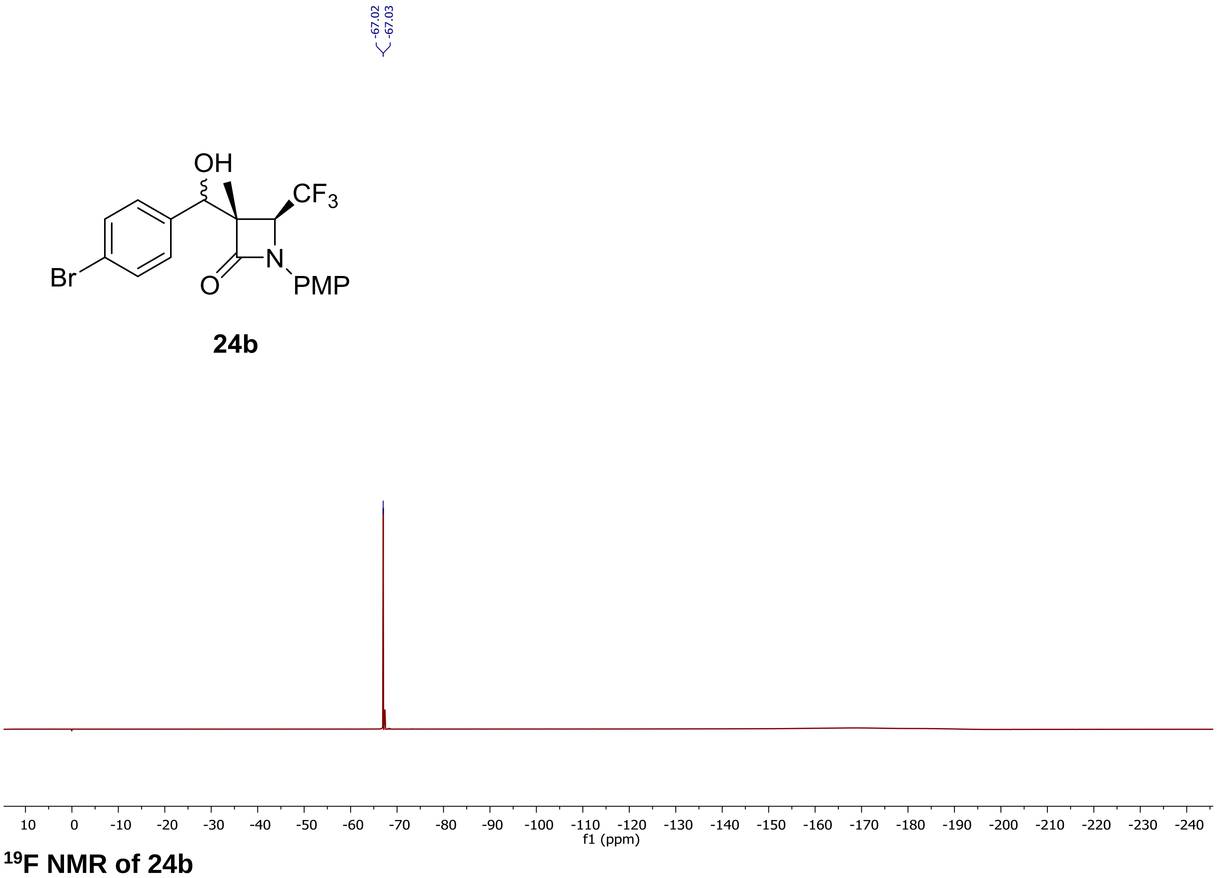

^{19}F NMR of 24b

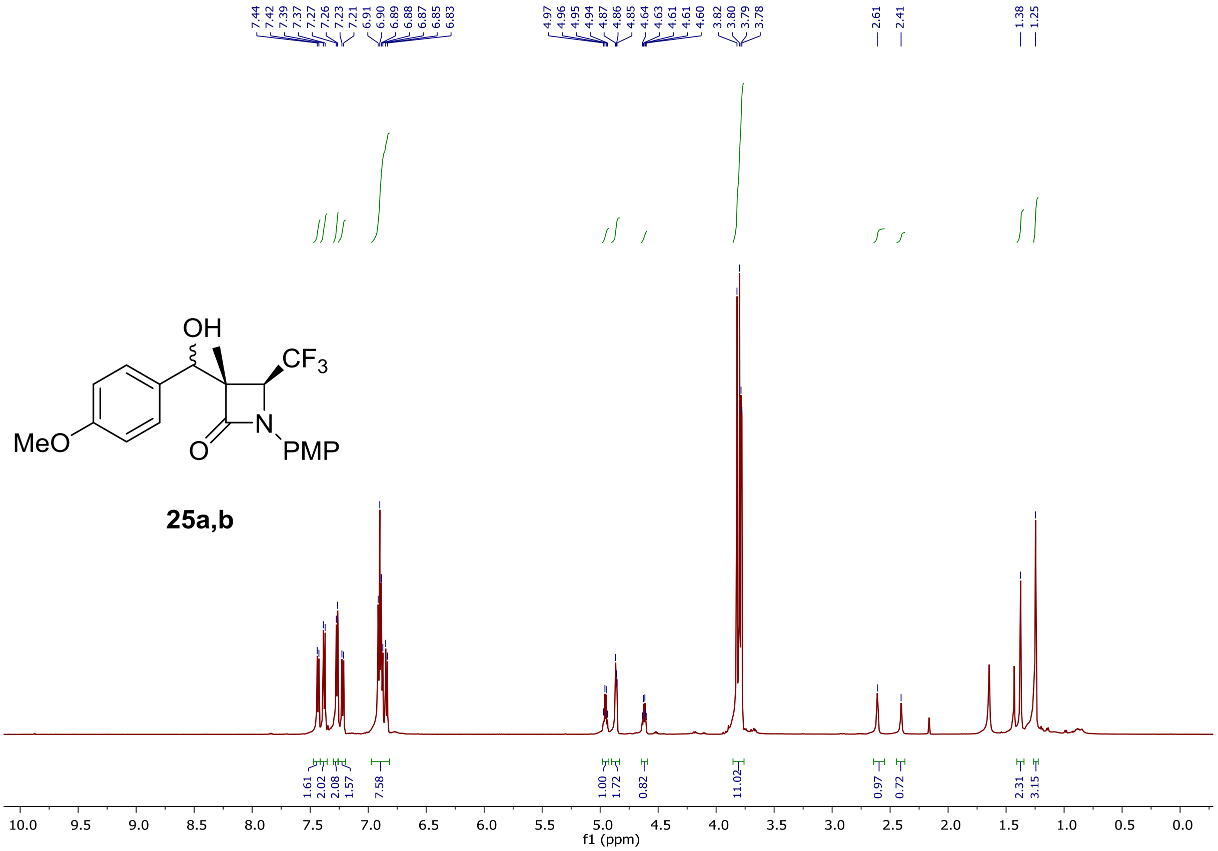

^{1}H NMR of 25a and 25b

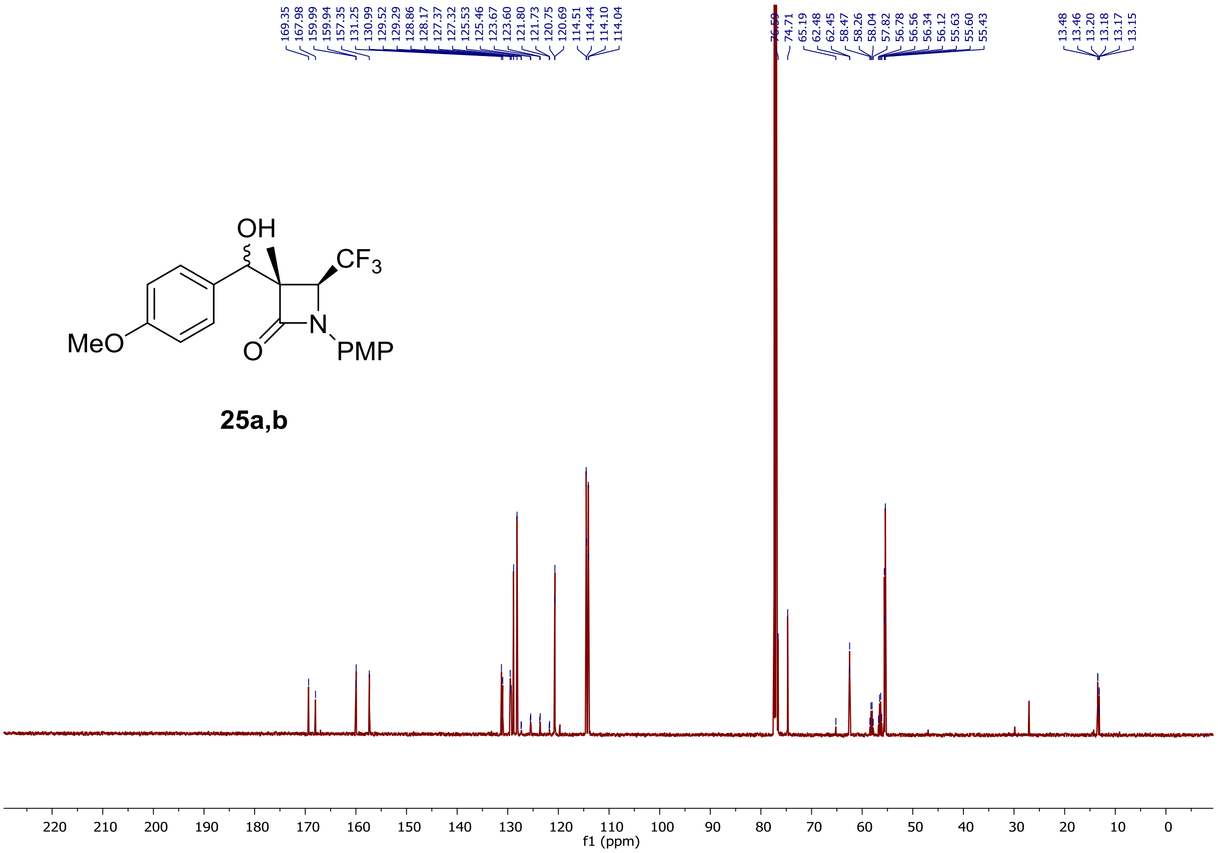

^{13}C NMR of 25a and 25b

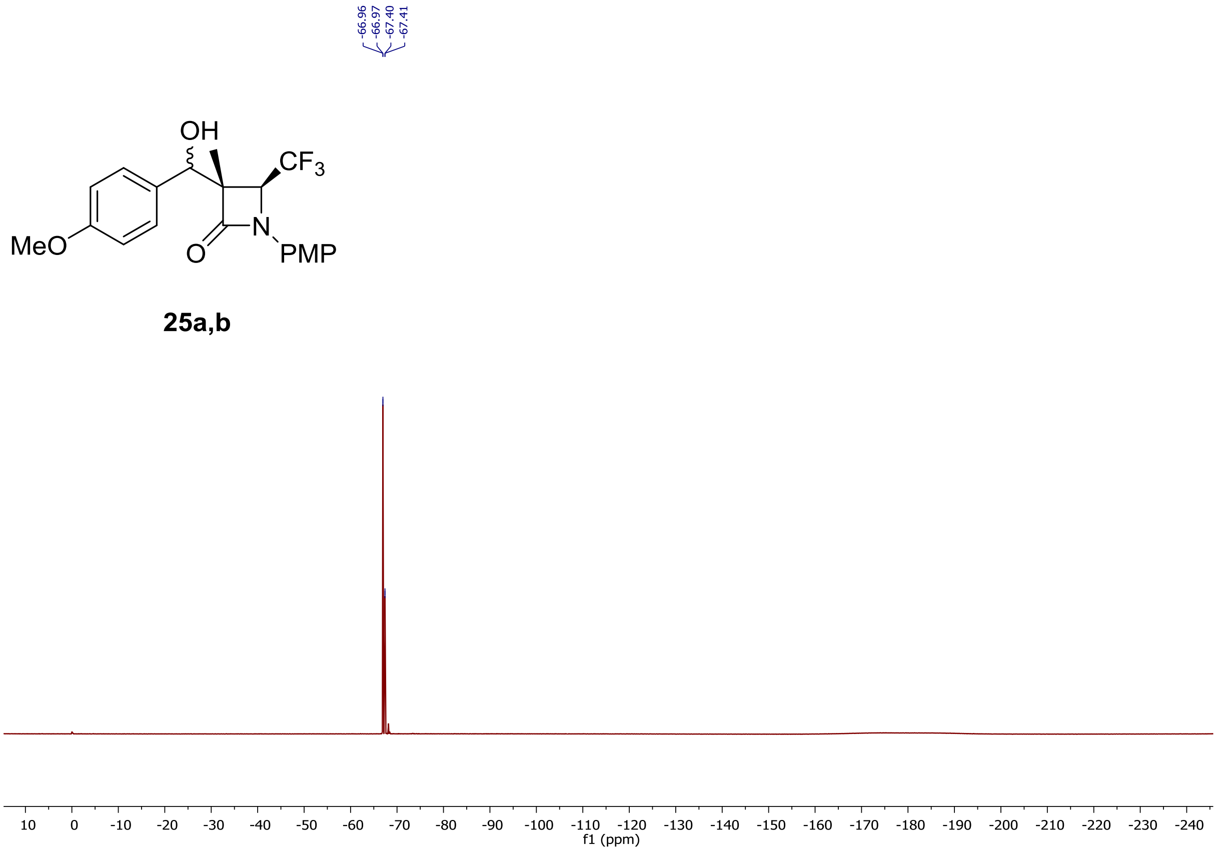

^{19}F NMR of 25a and 25b

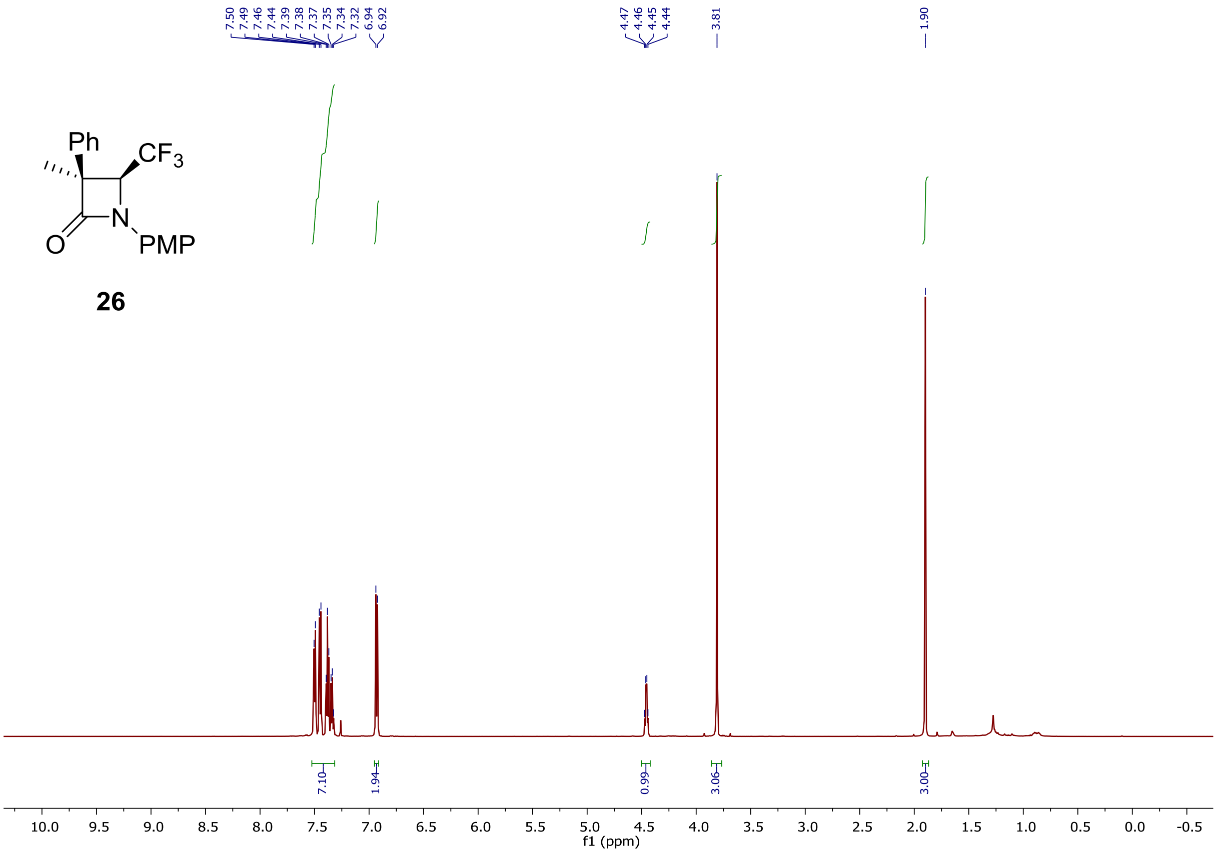

^{1}H NMR of 26

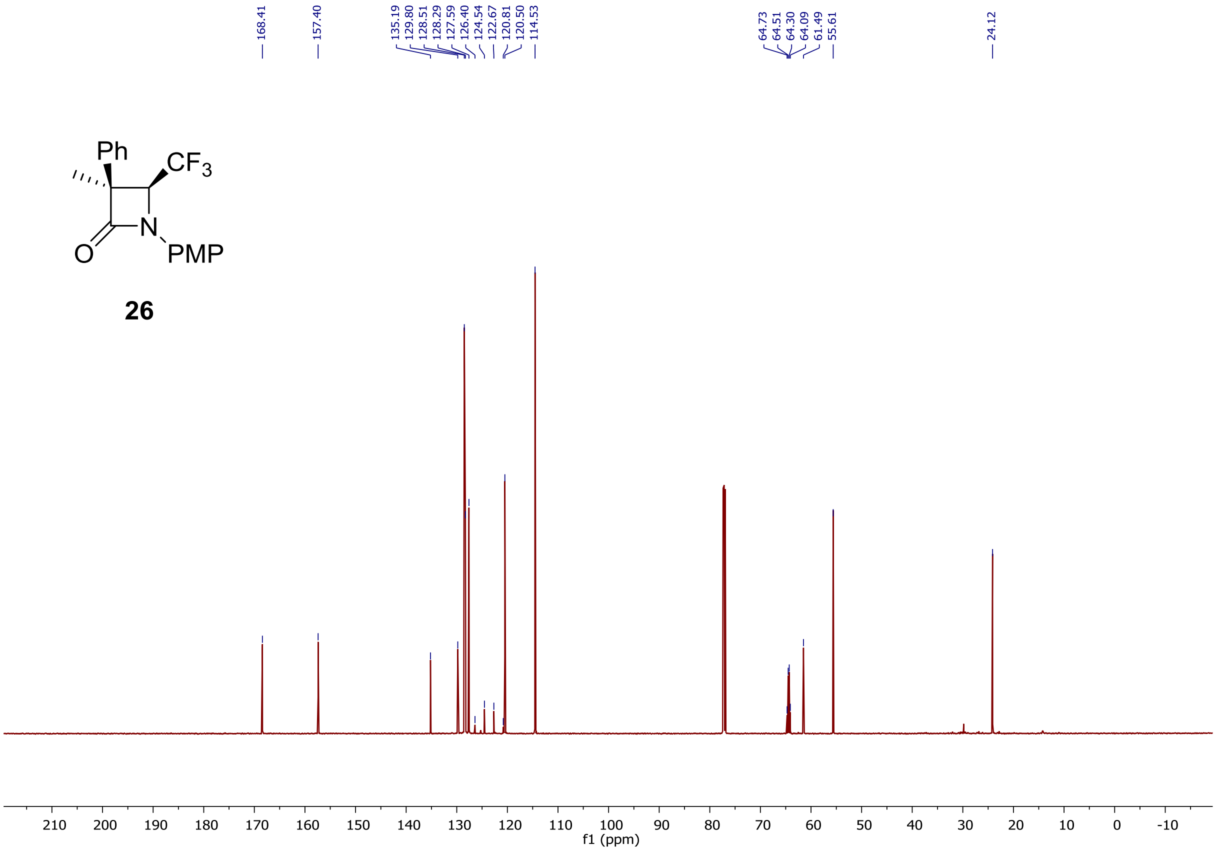

^{13}C NMR of 26

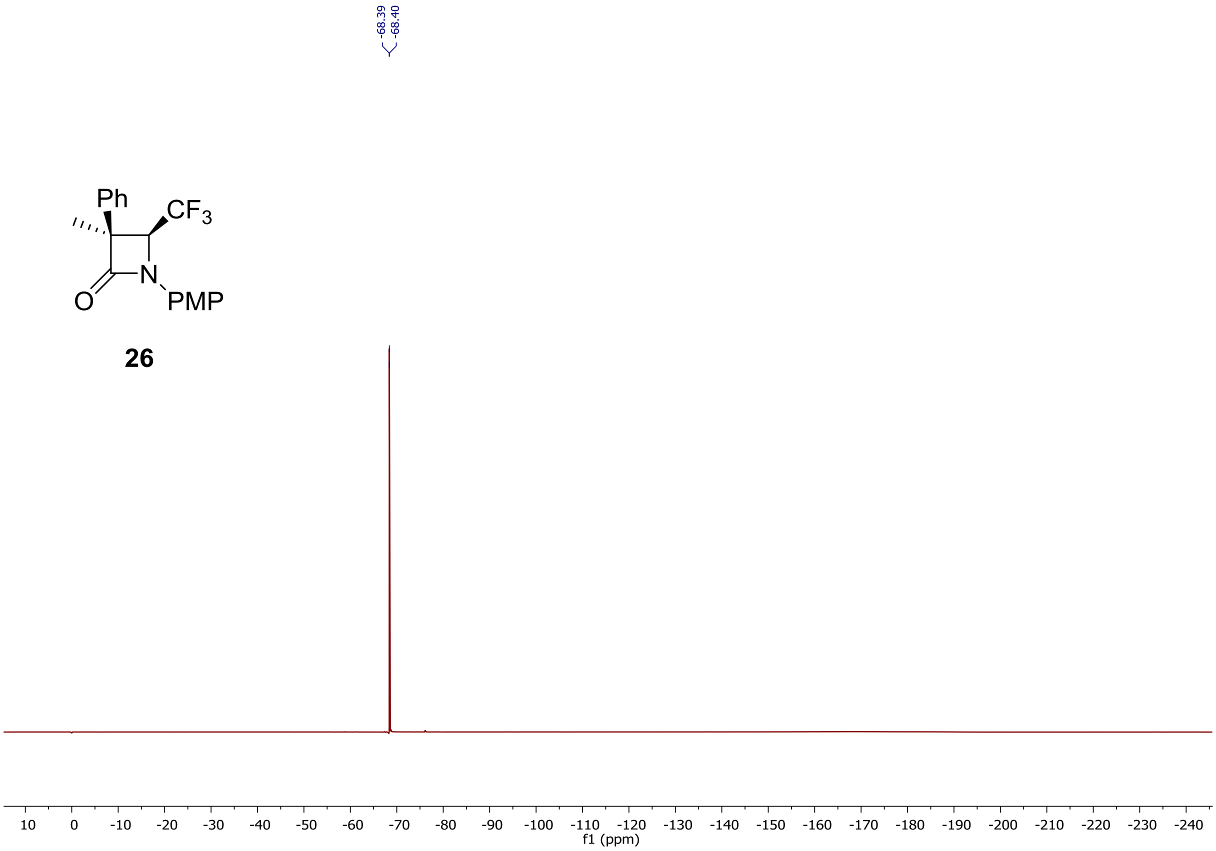

^{19}F NMR of 26

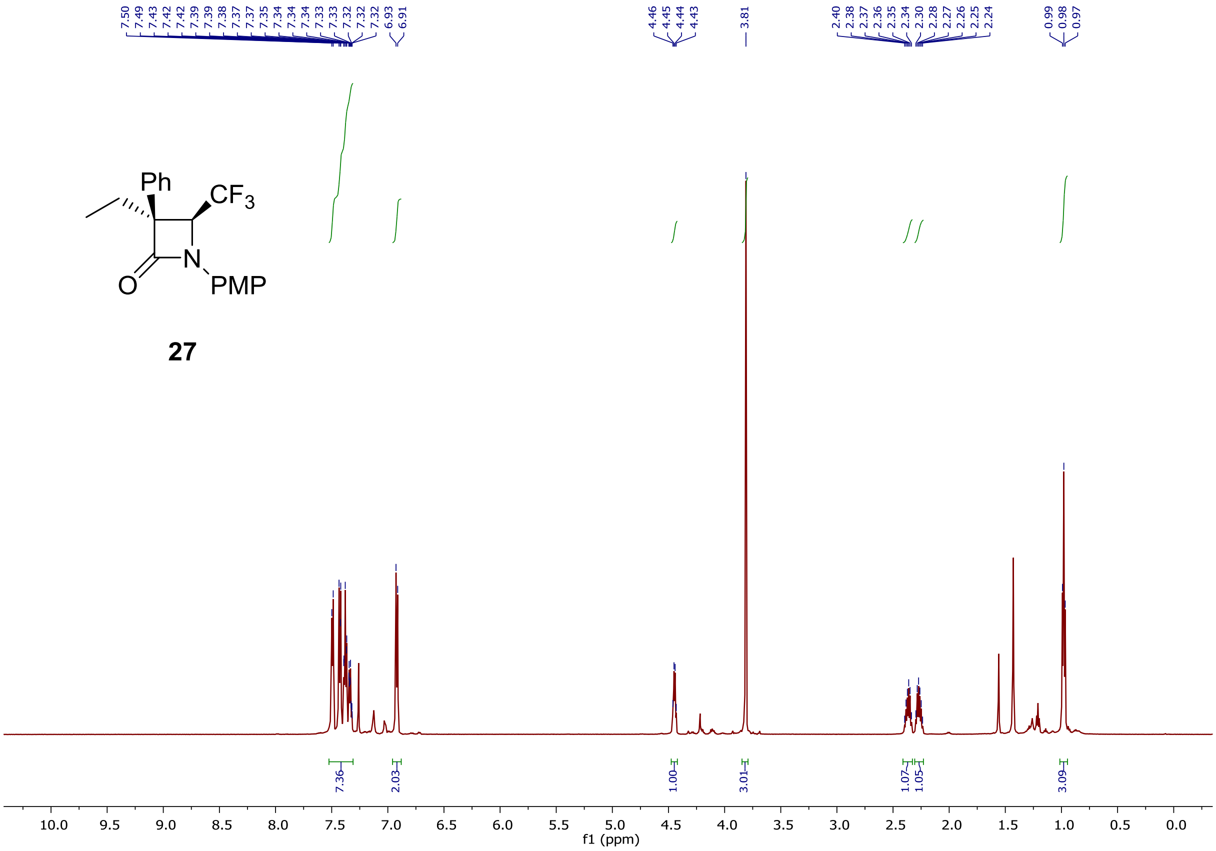

^{1}H NMR of 27

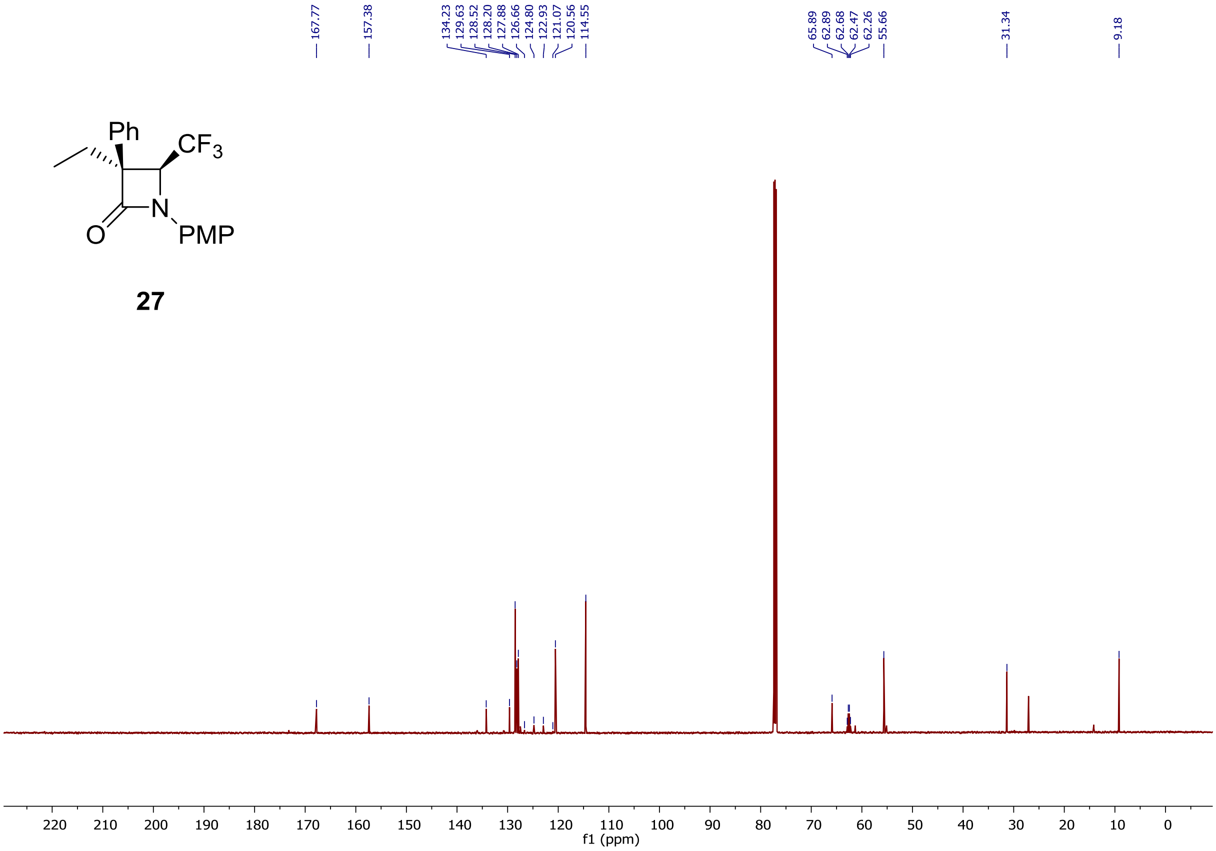

^{13}C NMR of 27

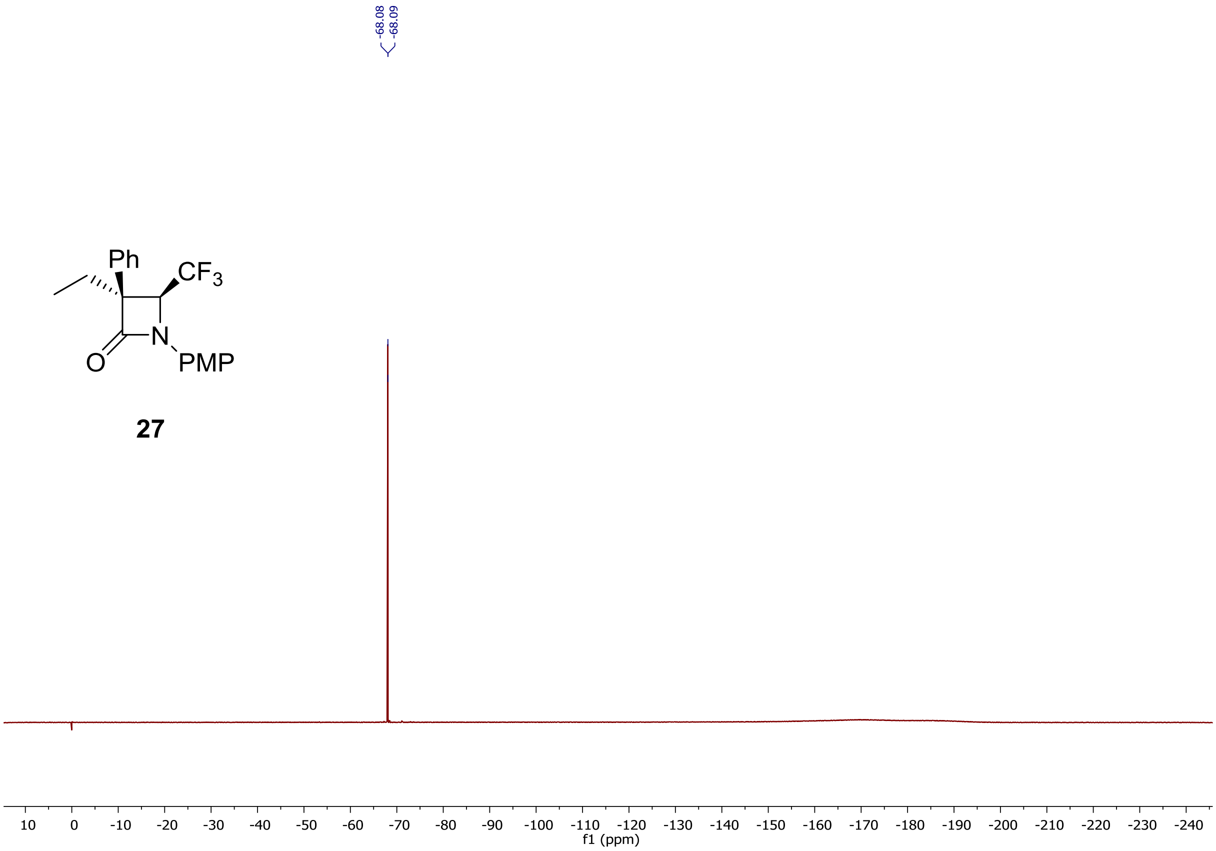

^{19}F NMR of 27

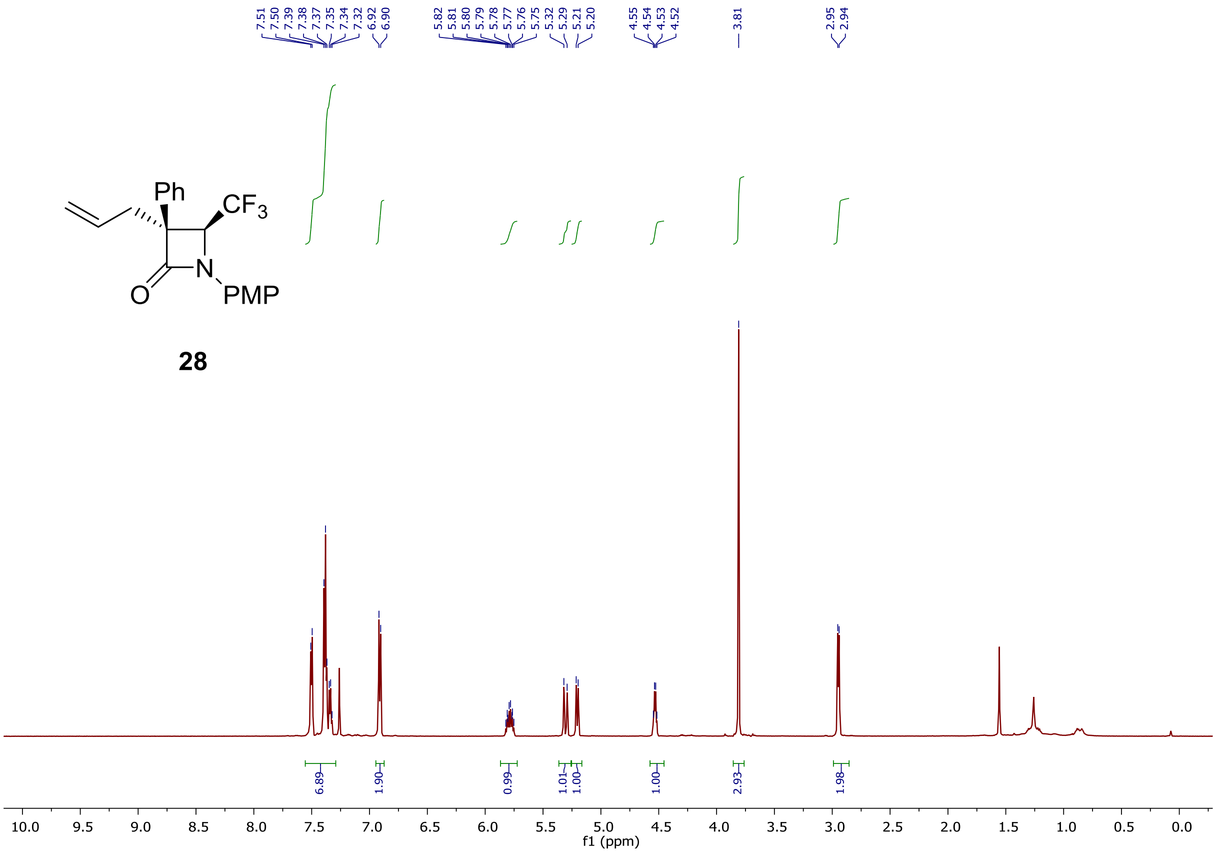

^{1}H NMR of 28

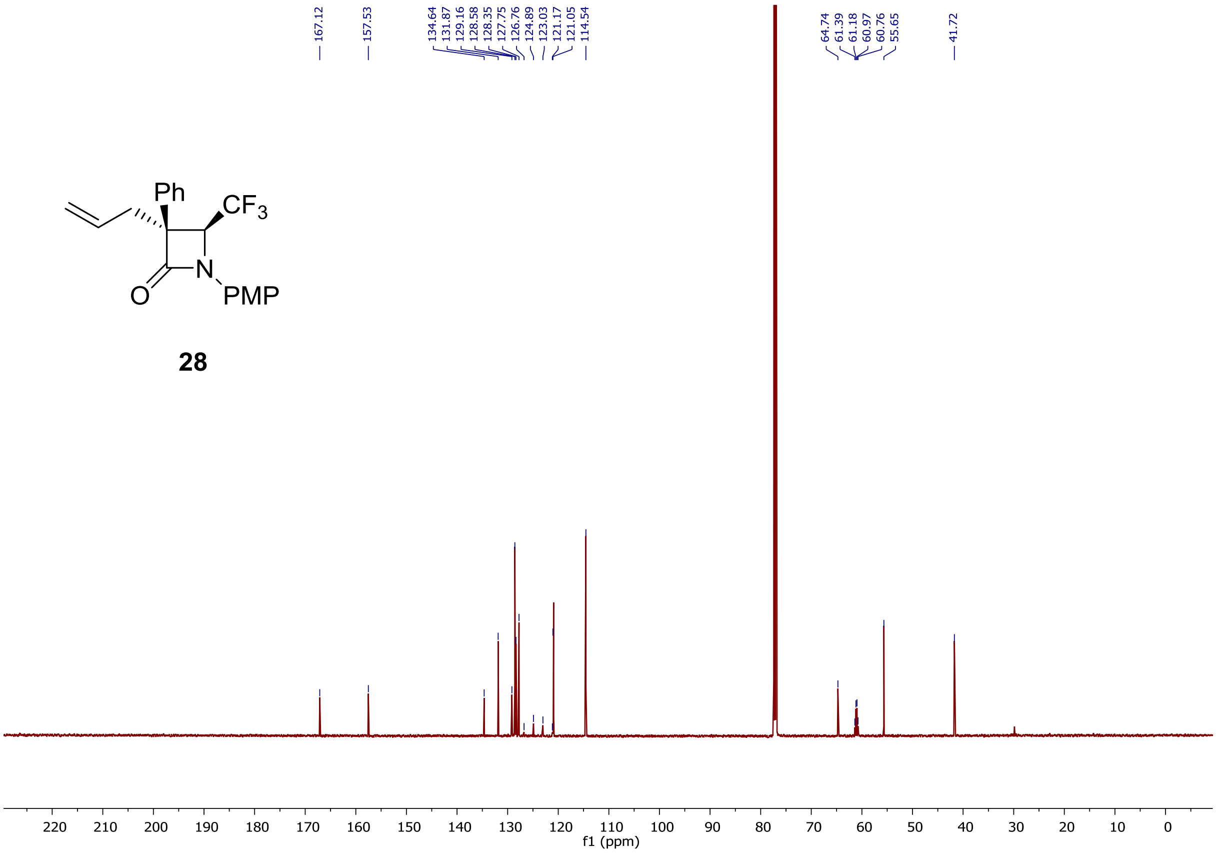

^{13}C NMR of 28

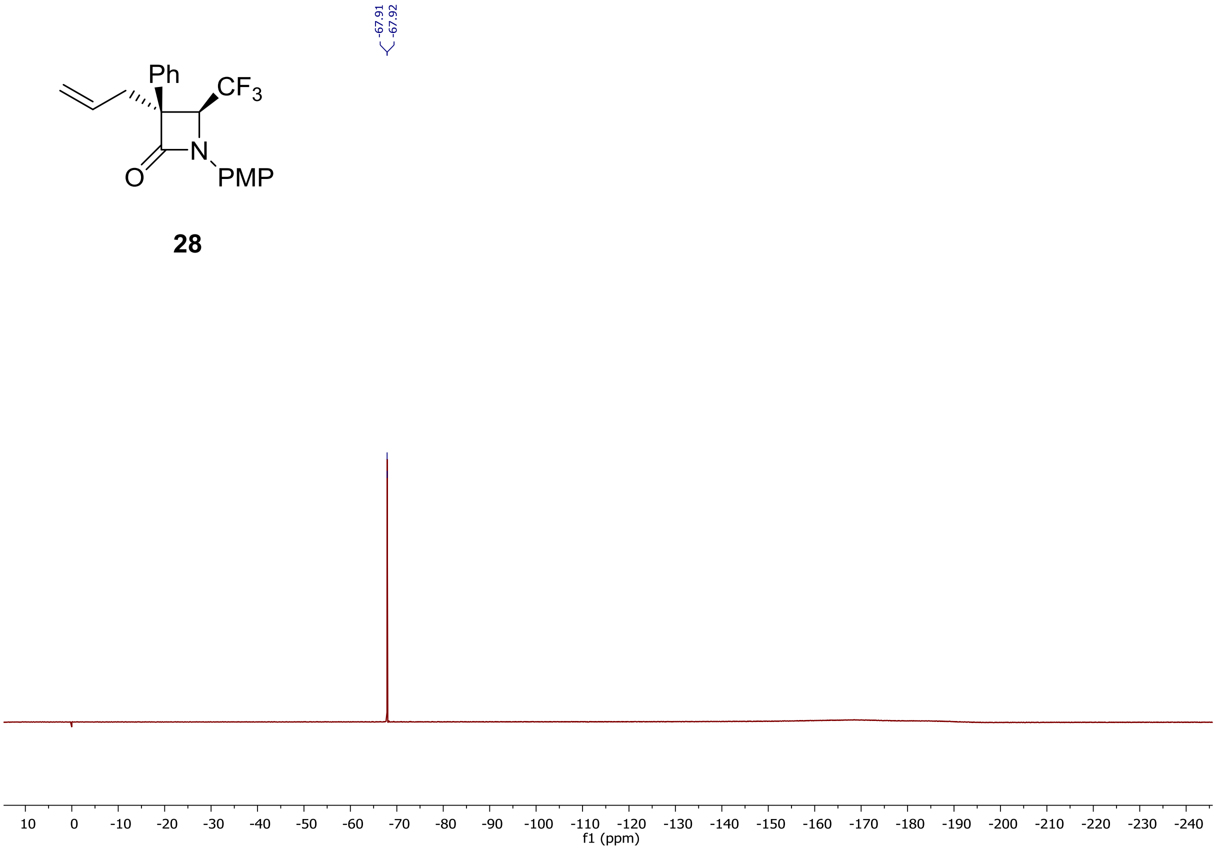

^{19}F NMR of 28

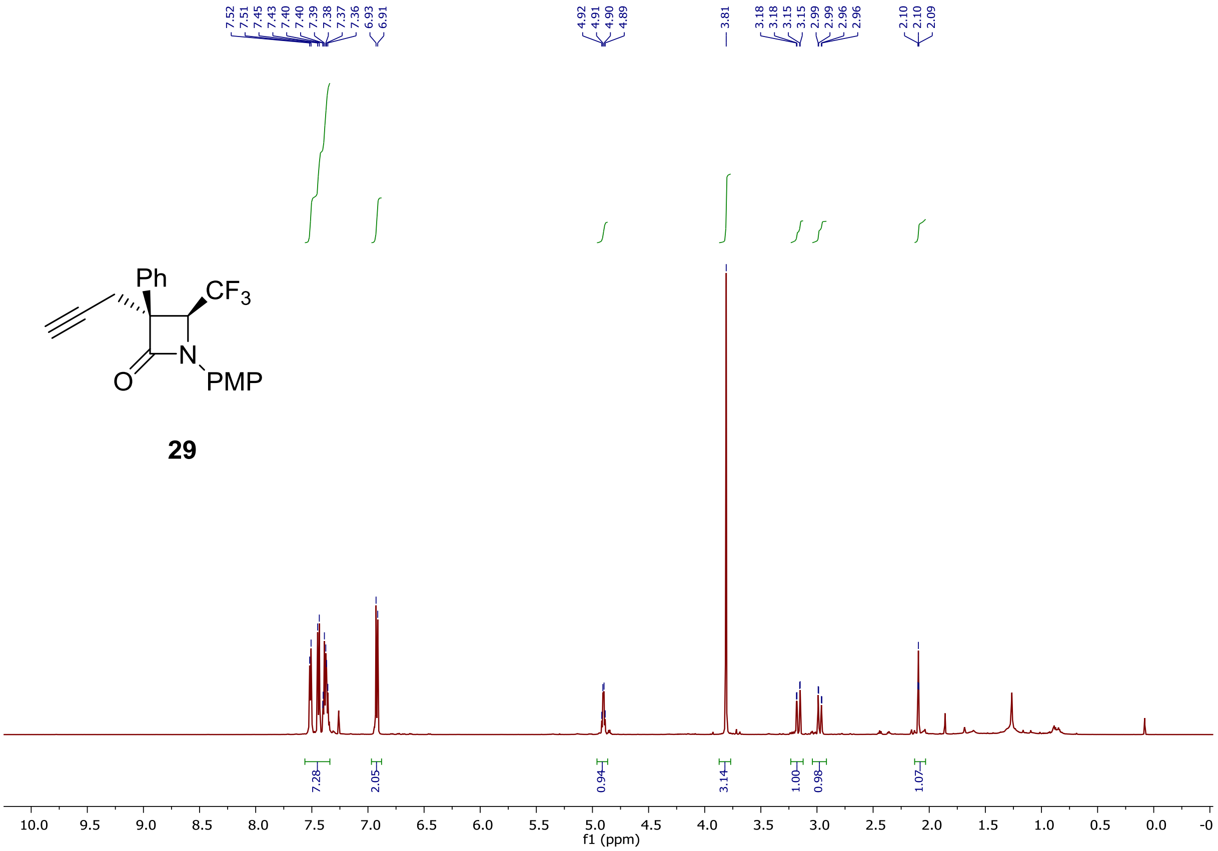

^{1}H NMR of 29

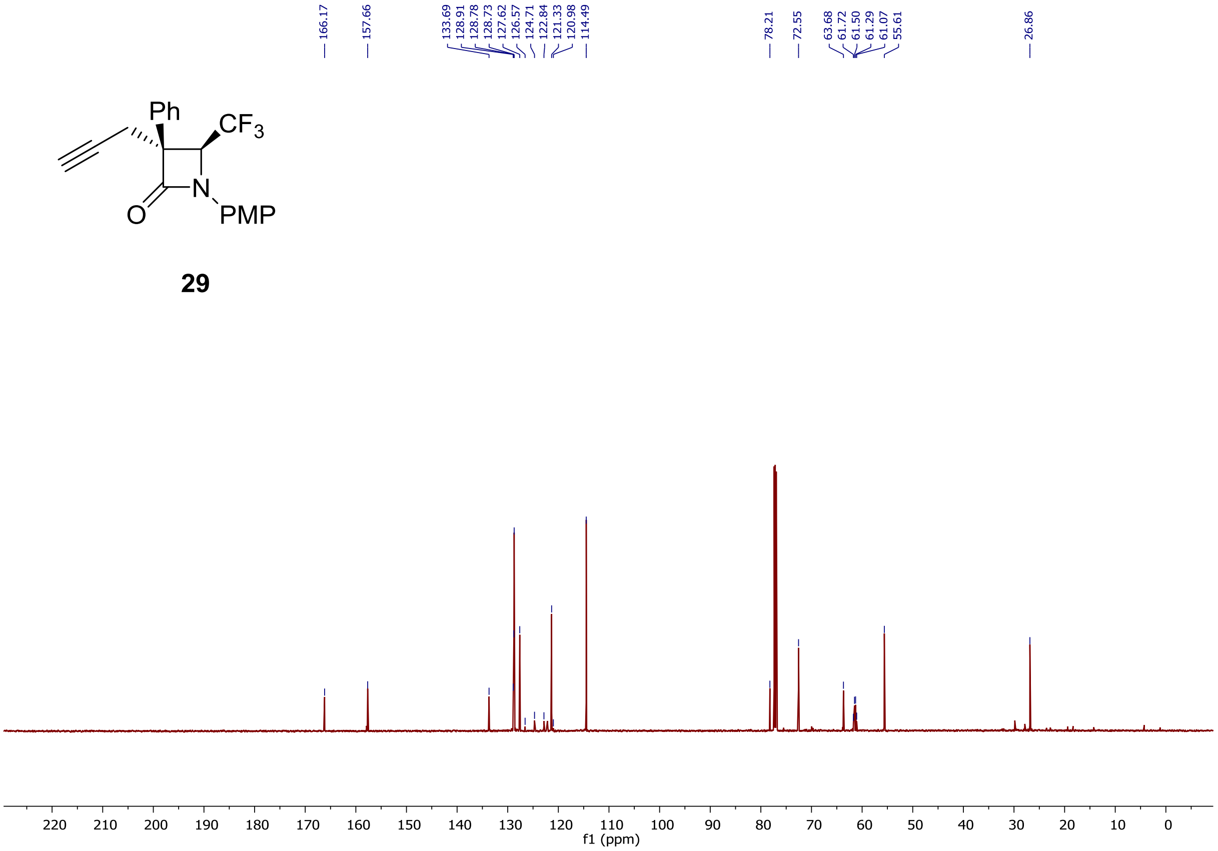

^{13}C NMR of 29

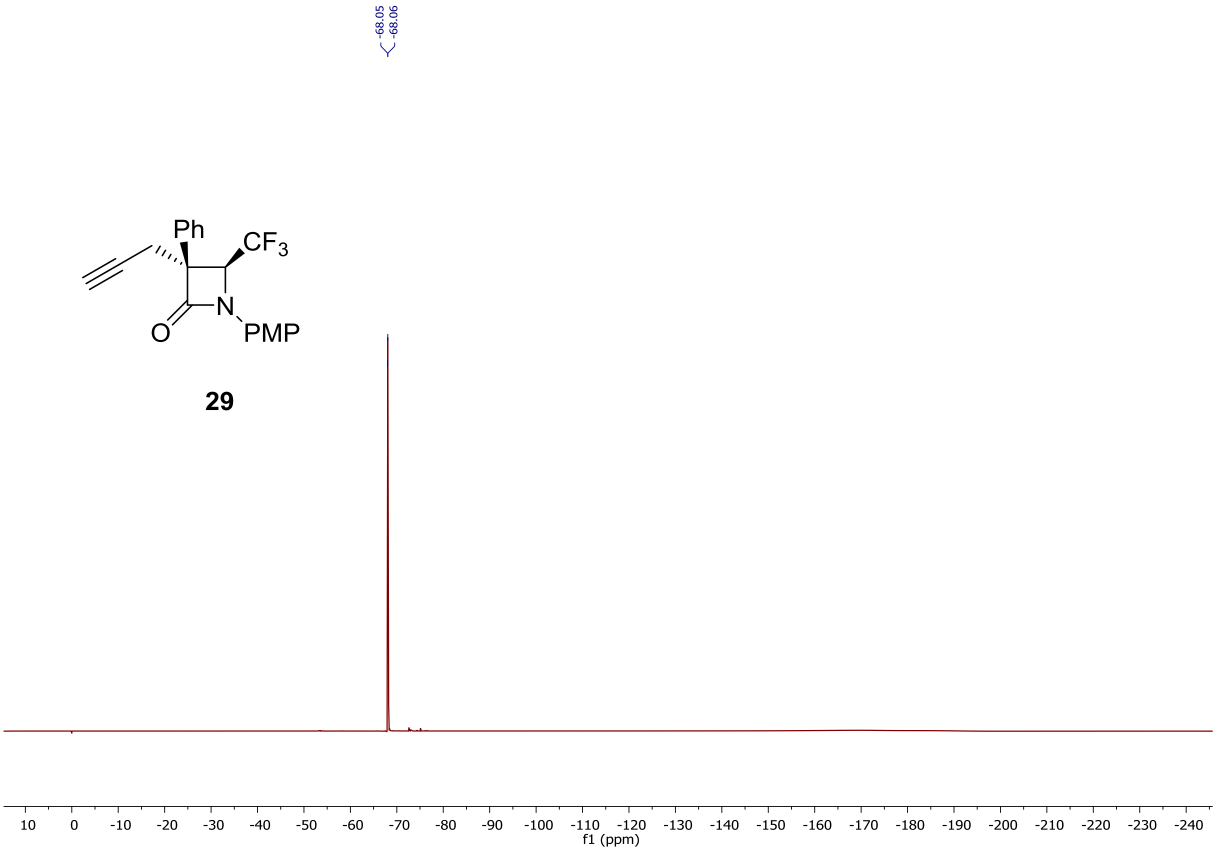

^{19}F NMR of 29

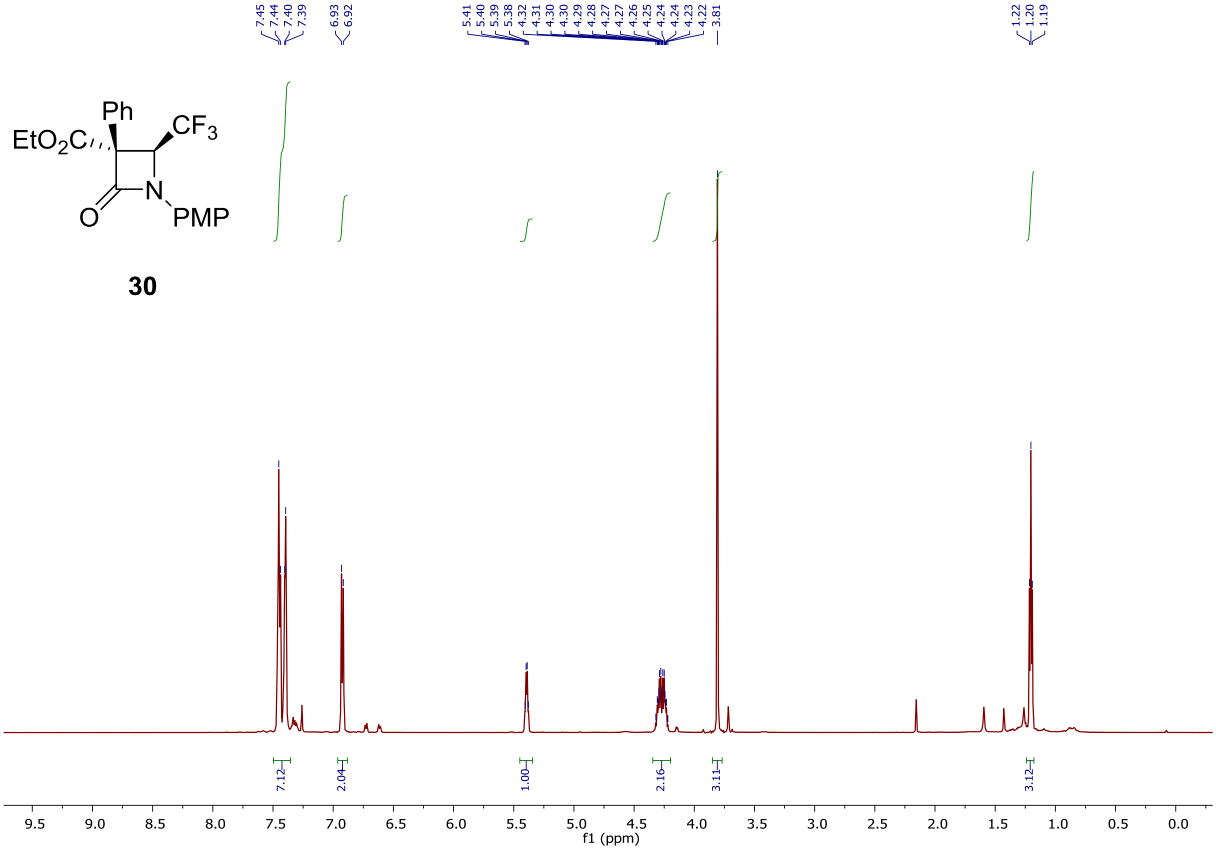

^{1}H NMR of 30

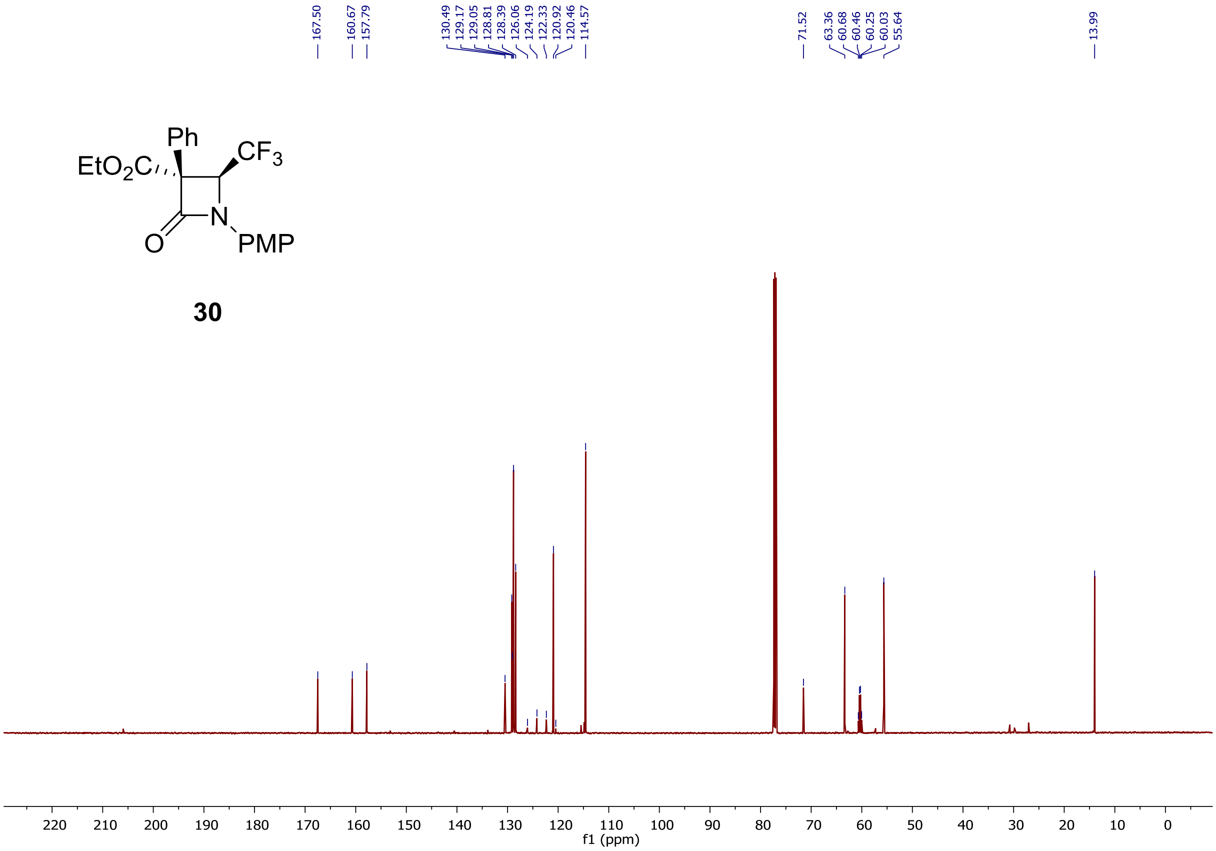

^{13}C NMR of 30

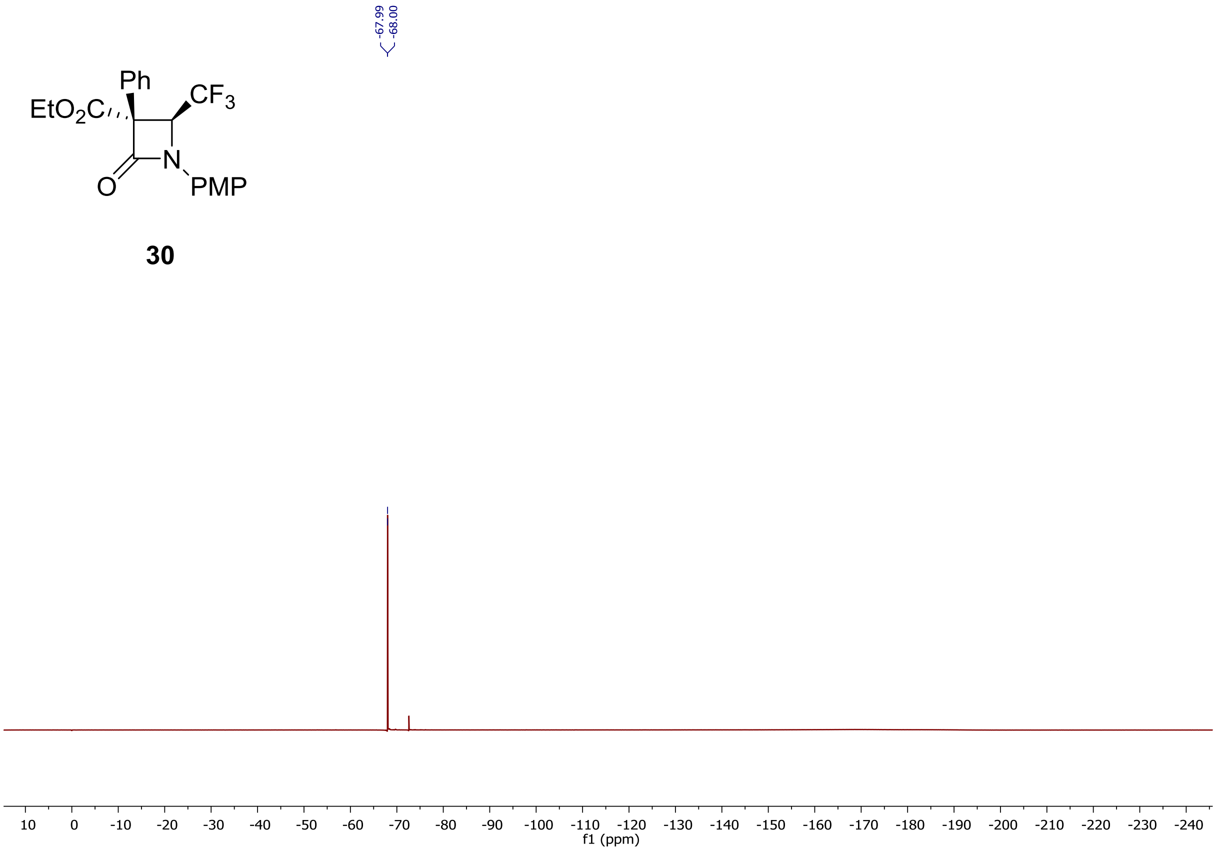

^{19}F NMR of 30

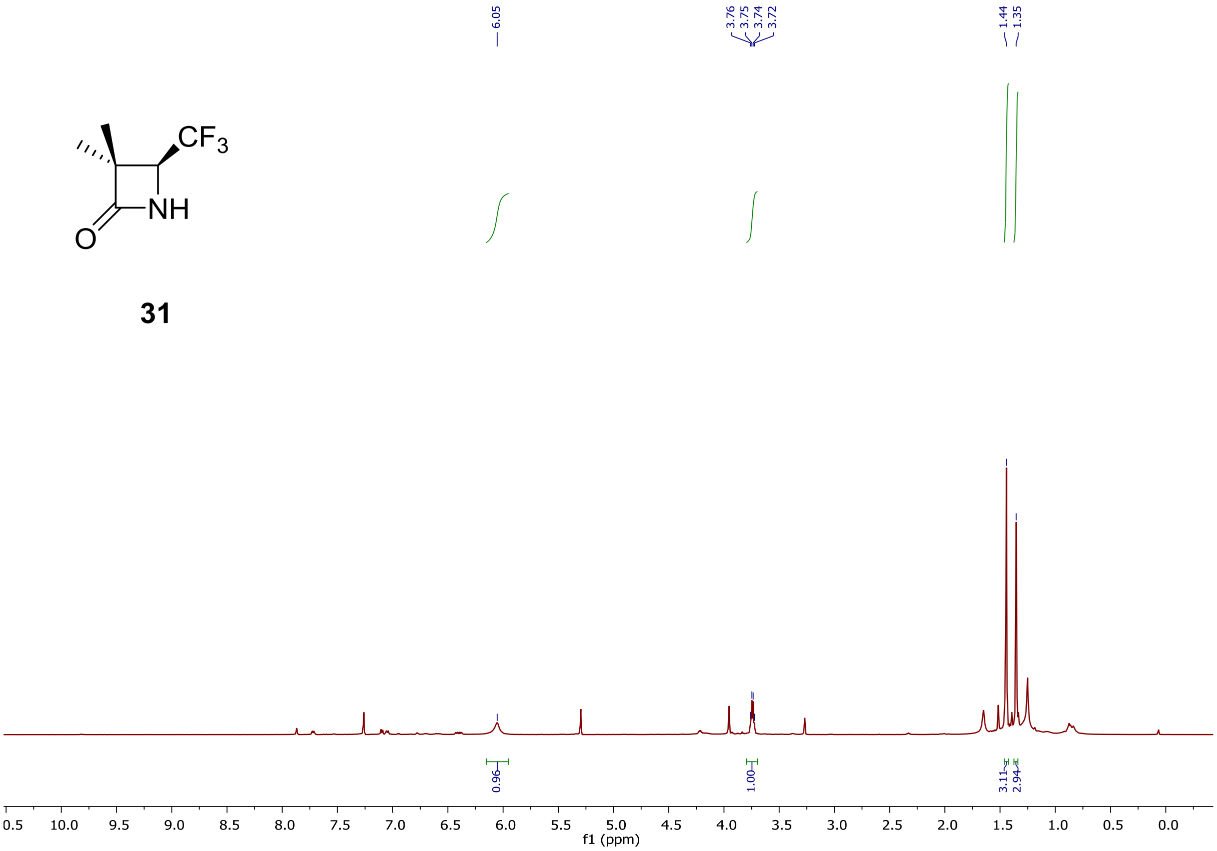

^{1}H NMR of 31

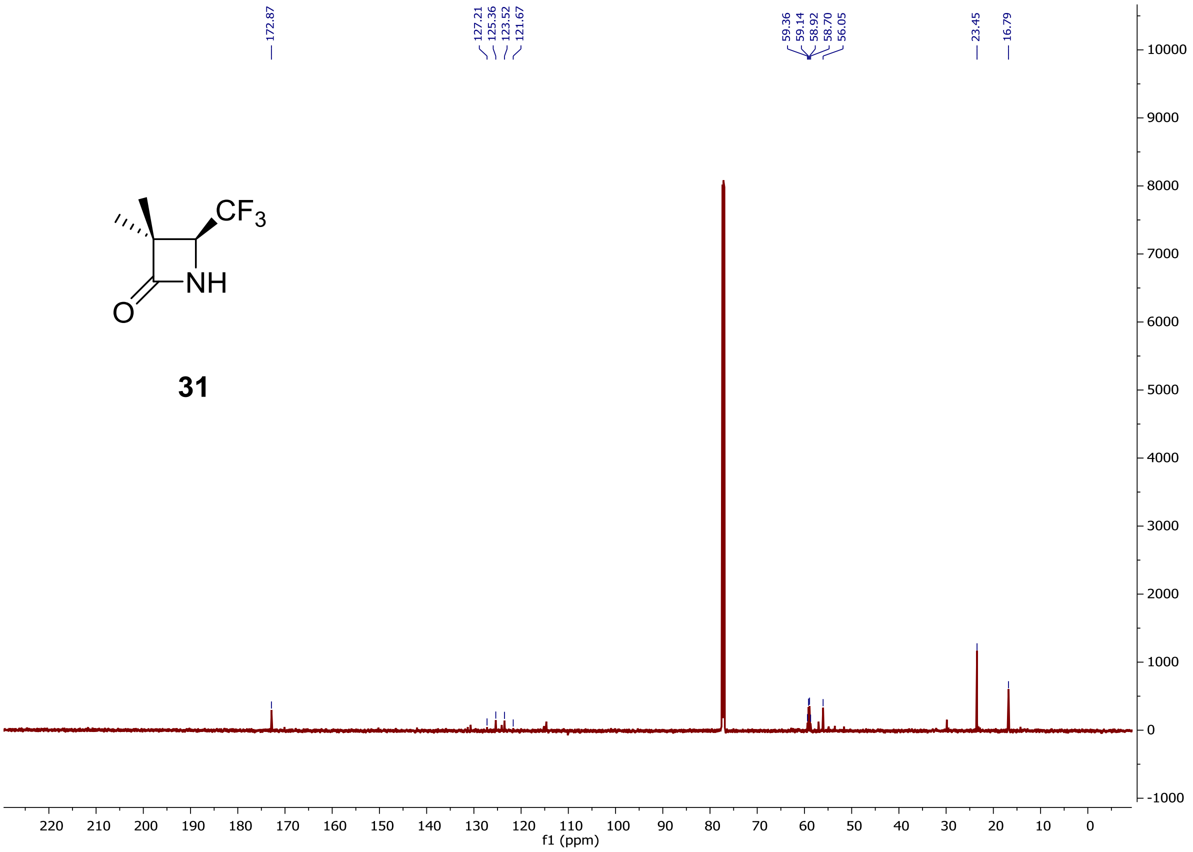

^{13}C NMR of 31

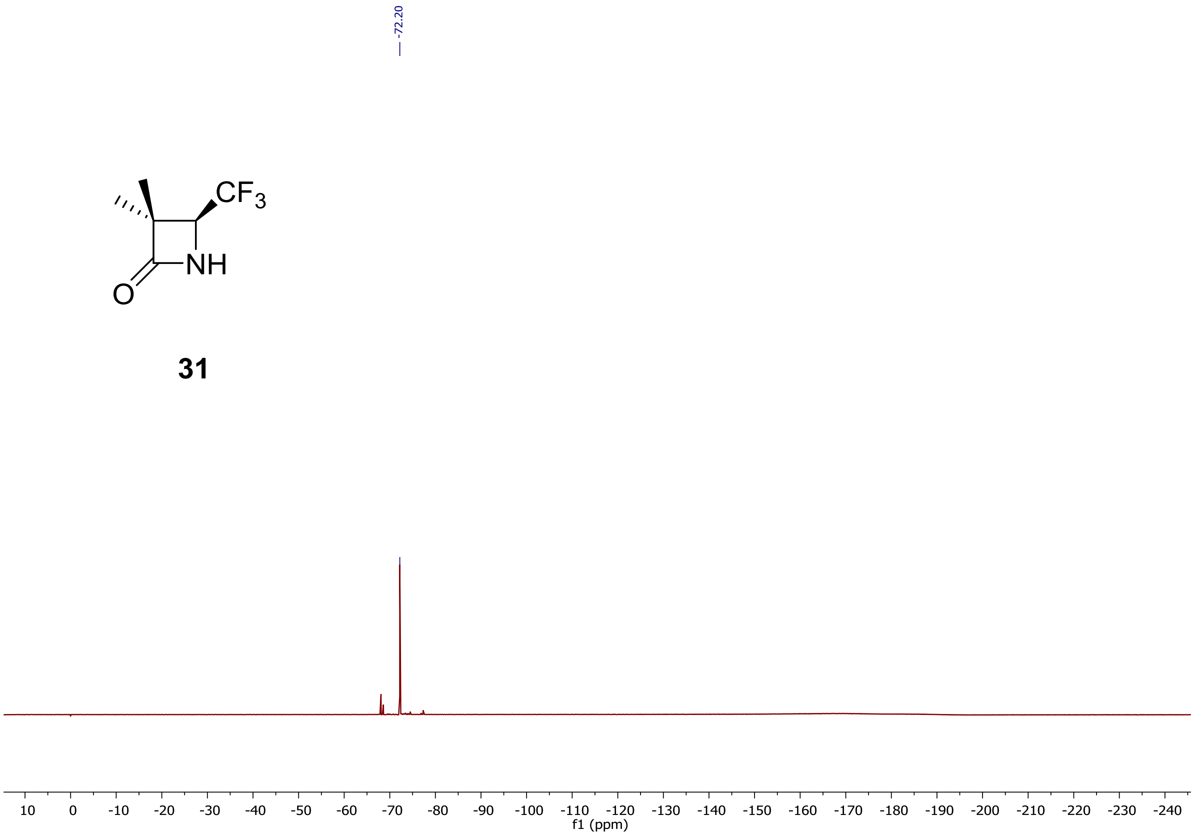

^{19}F NMR of 31

2. Example of 2D HOESY ^1H-^{19}F NMR spectrum

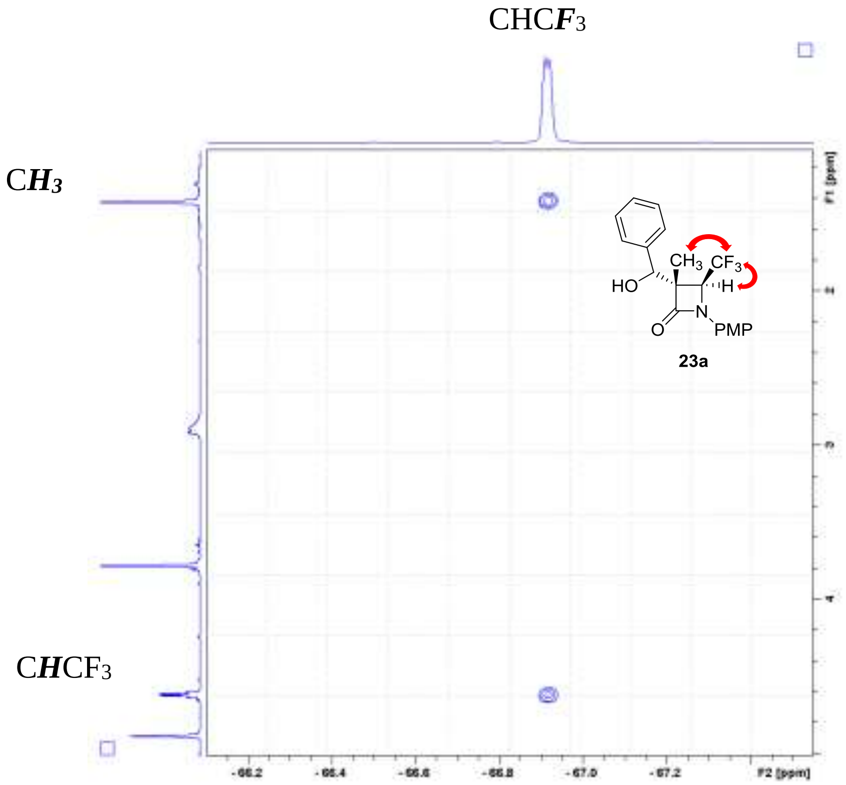

Figure1. 2D ^1H-^{19}F HOESY NMR spectrum of compound **23a**
